# Holocene life and microbiome profiling in ancient tropical Lake Chalco, Mexico

**DOI:** 10.1038/s41598-021-92981-8

**Published:** 2021-07-05

**Authors:** Bárbara Moguel, Liseth Pérez, Luis D. Alcaraz, Jazmín Blaz, Margarita Caballero, Israel Muñoz-Velasco, Arturo Becerra, Juan P. Laclette, Beatriz Ortega-Guerrero, Claudia S. Romero-Oliva, Luis Herrera-Estrella, Socorro Lozano-García

**Affiliations:** 1 Instituto de Geología, Universidad Nacional Autónoma de México, 04510 Mexico City, Mexico; 2 Laboratorio Internacional de Genoma Humano (LIIGH), Universidad Nacional Autónoma de México, 04510 Mexico City, Mexico; 3 Facultad de Ciencias, Universidad Nacional Autónoma de México, 04510 Mexico City, Mexico; 4 Instituto de Geofísica, Universidad Nacional Autónoma de México, 04510 Mexico City, Mexico; 5 Instituto de Investigaciones Biomédicas, Universidad Nacional Autónoma de México, 04510 Mexico City, Mexico; 6 Tecnologico de Monterrey, Escuela de Ingeniería y Ciencias, Centro de Bioingenieria, Av. Epigmenio González, No. 500, Fracc. San Pablo, 76130 Querétaro, Mexico; 7 Institut für Geosysteme und Bioindikation, Technische Universität Braunschweig, 38106 Braunschweig, Germany; 8 Centro de Estudios Atitlán, Universidad del Valle de Guatemala, 7001 Atitlán-Sololá, Guatemala; 9 Laboratorio Nacional de Genómica para la Biodiversidad (LANGEBIO), Centro de Investigación y de Estudios Avanzados del Instituto Politécnico Nacional (CINVESTAV), Km 9.6 Libramiento Norte Carretera Irapuato-León, 36821 Irapuato, Guanajuato Mexico; 10 Institute of Functional Genomics for Abiotic Stress, Texas Tech University, Lubbock, Texas 79410 USA

**Keywords:** Ecology, Environmental sciences, Limnology

## Abstract

Metagenomic and traditional paleolimnological approaches are suitable to infer past biological and environmental changes, however, they are often applied independently, especially in tropical regions. We combined both approaches to investigate Holocene Prokaryote and Eukaryote diversity and microbial metabolic pathways in ancient Lake Chalco, Mexico. Here, we report on diversity among a large number of lineages (36,722 OTUs) and functional diversity (27,636,243 non-clustered predicted proteins, and 6,144 annotated protein-family genes). The most abundant domain is Bacteria (81%), followed by Archaea (15%) and Eukarya (3%). We also determined the diversity of protein families and their relationship to metabolic pathways. The early Holocene (> 11,000 cal years BP) lake was characterized by cool, freshwater conditions, which later became warmer and hyposaline (11,000–6,000 cal years BP). We found high abundances of cyanobacteria, and fungi groups associated with mature forests in these sediments. Bacteria and Archaea include mainly anaerobes and extremophiles that are involved in the sulfur, nitrogen, and carbon cycles. We found evidence for early human impacts, including landscape modifications and lake eutrophication, which began ~ 6,000 cal years BP. Subsaline, temperate conditions were inferred for the past 5,000 years. Finally, we found nitrogen-fixing bacteria and protein-family genes that are linked to contaminated environments, as well as several fungal pathogens of crops in near-surface sediments.

## Introduction

**Figure 1:**
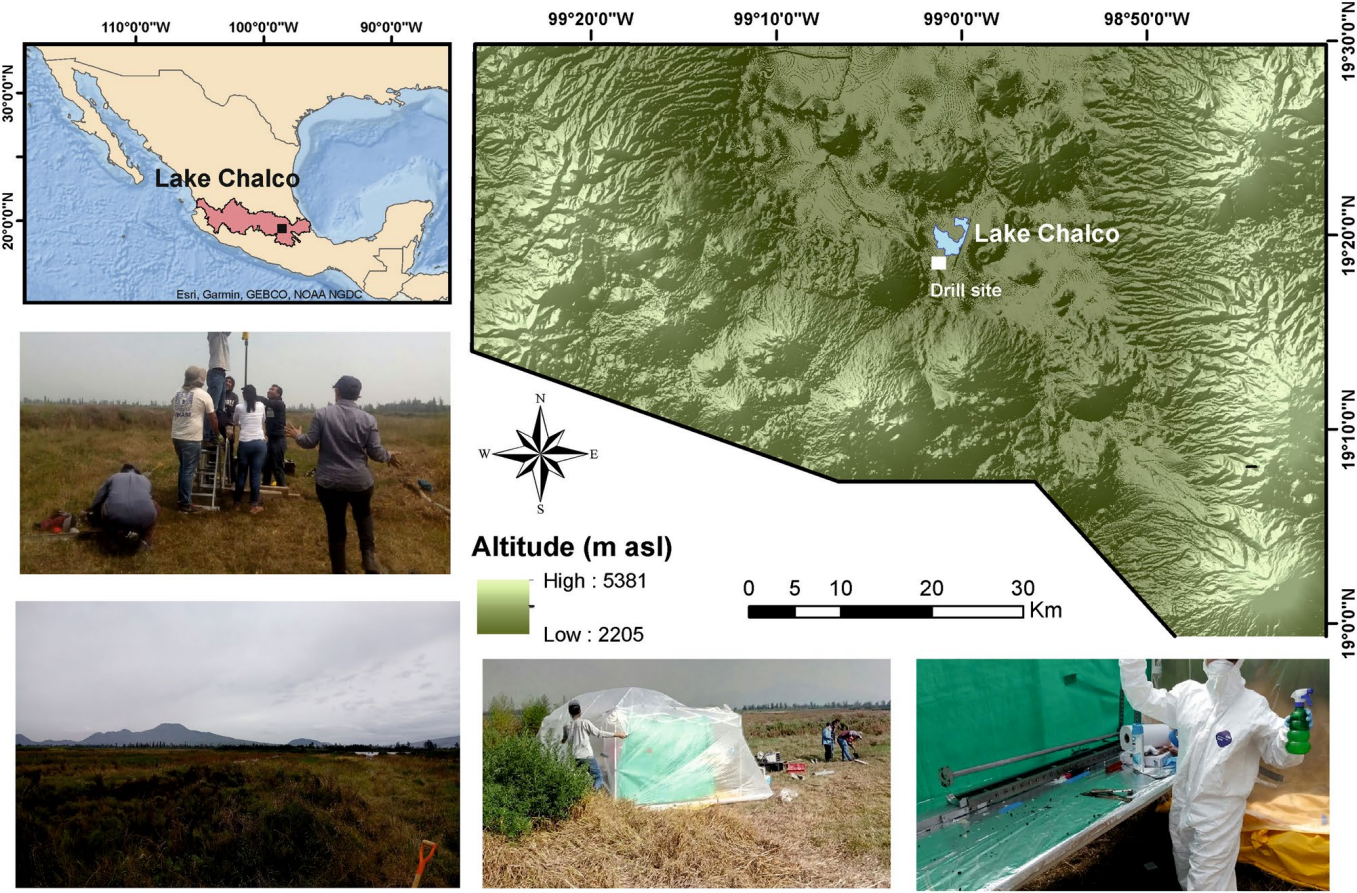
(Top left) Map of Mexico showing the Trans-Mexican Volcanic Belt (TMVB, red area) and the location of Lake Chalco (black square), southeast of Mexico City, and (Top right) the drill site (white square). Images illustrate the field laboratory and drill operations. The map was created with ArcGIS Desktop 10.6.1 (URL: http://www.esri.com) and CorelDRAW 2020 version 22.0.

The onset of the recent interglacial (11,700 cal years BP) was marked by substantial climate and environmental changes^1^. Temperature increases during the Early Holocene caused glaciers to retreat, modified landscapes, and reshaped plant and aquatic communities worldwide, even in the tropical latitudes of Mesoamerica^2–5^. Thermal regimes and hydrological budgets of lakes changed, and aquatic communities were impacted by these shifts^6,7^. Lake Chalco (Fig. 1) is a high-altitude (2,200 m asl) tropical water body in central Mexico and its sediments contain a high-resolution archive of late Quaternary climate and environmental change^8^.

To date, studies of past aquatic and terrestrial communities have focused on the analysis of fossil diatoms and pollen, respectively^8^. Such traditional paleolimnological methods are tremendously useful, but do not provide information about organisms that lack hard parts that can become fossilized^9,10^. Novel genomics approaches, such as barcoding (simple DNA sequencing), metabarcoding (mass DNA sequencing of multiple taxa in a single sample), metagenomics, metatranscriptomics and proteomics, enable the collection of information about a large number of lineages, and facilitate reconstruction of complete genomes and metabolic attributes of the biota. For instance, metagenomic analyses of lake sediments can reveal detailed information about diversity and community composition, providing insights into modern and past ecological dynamics^11–19^. Information on contemporary lake inhabitants and conditions is obtained through analysis of modern environmental DNA (eDNA)^20^, which in turn can be used to better understand past environmental conditions and biological communities, evaluated through ancient DNA (aDNA)^11–14,21^ analysis. Past environments, especially those subject to anthropogenic disturbances, are best understood using a combination of conventional micropaleontological techniques and metagenomic approaches, which reveal phylogenetic and biochemical traits^21–23^. Numerous preserved genes yield information about metabolic pathways used by organisms in past communities^24^, thereby providing insights into the development of adaptation strategies such as antibiotic resistance and ecotoxicological effects of pollutants^25^, as well as evolutionary processes and species replacement. Our study fits into the context of the new and rapidly advancing field in paleoecological research, using younger (sedDNA) and ancient (sedaDNA) lake sediment DNA, to track long-term changes in both terrestrial and aquatic biota^10,20,21,26^.

**Figure 2:**
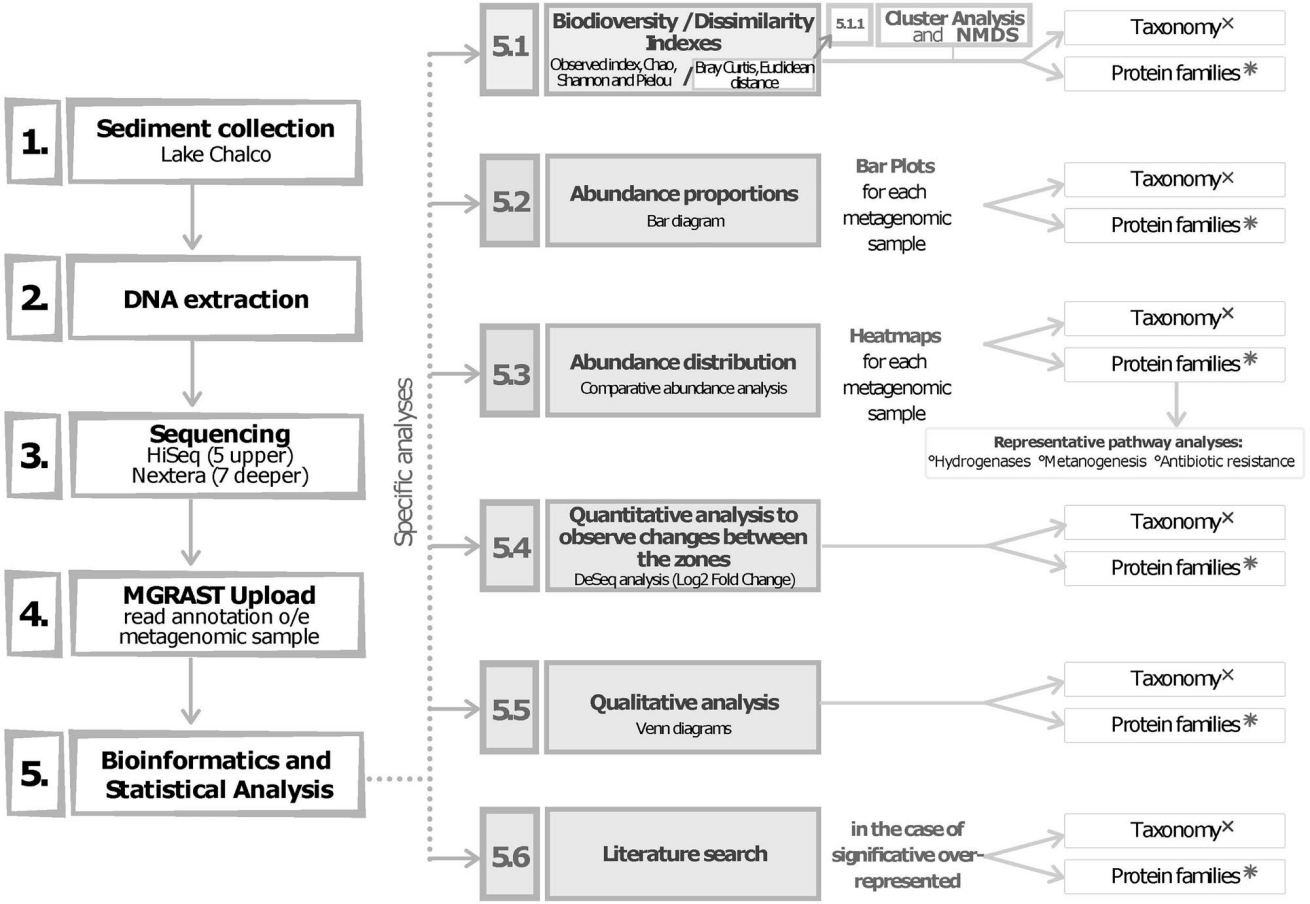
Schematic representation of the field methods, laboratory methods, and bioinformatic analyses associated with the retrieved Holocene sediment sequence from Lake Chalco, Mexico. Numbers 1 to 5 represent methodological phases and numbers 5.1 to 5.6 represent bioinformatics and statistical analyses run on the generated data. “x” represents taxonomy determined to genus level and “*” represents protein families (pfams) from identified genes. DNA—Deoxyribonucleic Acid; HiSeq—High-throughput Sequencing Illumina; Log2 Fold Change—based on DeSeq analysis of R packages; Nextera—sequencing on Illumina; MG RAST—Metagenomics open service analysis. Edited in CorelDRAW 2020 version 22.0.

The Basin of Mexico is of great paleontological importance. For instance, there are abundant reports of Pleistocene megafauna from the region^27,28^, whereas the Holocene was characterized by dense pre-Columbian human occupation^29^. Despite the potential of sed(a)DNA analysis to reveal past Prokaryote and Eukaryote diversity in the region, our study is the first to combine both metagenomics and traditional micropaleontology. Sediments in the basin of Lake Chalco contain a continuous, ~ 400-ka paleoclimate and paleoenvironment history, representing one of the longest such records from the transition between the Nearctic and Neotropical regions^3,8,30–36^. We retrieved a short, 270-cm-long sediment core from the dry lakebed, using a piston corer (Figs. 1 and 2, Supplementary Table S1), which was used to determine past changes in biotic diversity related to climate, environment and human impacts^29,37^. Our objective was to characterize Holocene environmental phases in Lake Chalco, using metagenomic, fossil diatom, and sediment geochemical analyses, well-dated volcanic (tephra) layers, and lithologic properties of the core^38^, as well as correlate our findings with inferences from other studies in the region^7,33,34,39^. Our aDNA, that is the fraction of eDNA buried in the sediment, which originated from organisms that are no longer physiologically active^21^, and micropaleontological and geochemical analyses were combined to assess present and past biodiversity. Furthermore, we identified and described protein families (pfams) that provide novel insights into the metabolic pathways in sediments of Lake Chalco (Fig. 2). Our results shed light on Holocene biodiversity^34,39^ and environmental conditions in the high-altitude American tropics.

## Results and discussion

### Evaluation of the lithological, geochemical, and fossil diatom evidence

We obtained a short, 270-cm sediment core (< 12,000 years BP) from high-altitude tropical Lake Chalco, central Mexico, which possesses a lacustrine sediment record of > 250 m^40^. Lake Chalco lies in the central region of the Trans-Mexican Volcanic Belt (Fig. 1). It is bounded to the north by the Sierra de Santa Catarina, to the east by the Sierra Nevada (Volcanoes Popocatepetl, Iztaccíhuatl, Tláloc and Telapón), and to the west, by Volcano Teuhtli, which is the closest volcano to the waterbody (~ 6.5 km)^41^. Lake sediments are composed mainly of clays that have been described as impermeable. The Lake Chalco Basin, however, is a highly complex system and seismically active. Therefore, the presence of active fractures during the Holocene is possible. Active fractures may result in inflow from the deep aquifer. Historical data indicate the existence of freshwater springs in upper parts of the aquifer, mainly at the eastern piedmont^42^. For instance, mineral and thermal waters at Peñón del los Baños, near the Mexico City airport (33 km from Lake Chalco), have been reported since Aztec times around AD 1,325. This spring is associated with a system of seismically active fractures. No thermal waters have been reported in modern Lake Chalco, however phreatomagmatic activity (> 100,000 years BP) from the Xico Volcano has been documented^43^. Recent studies showed that the water table position changes from the upper parts of the watershed to central Texcoco, 45 km from Lake Chalco. In this study four components of the flow system were identified, including waters of recent infiltration and local circulation, evidencing intermediate chemical evolution, and waters more chemically evolved of large flow trajectories and of deep circulation^44^.

**Figure 3:**
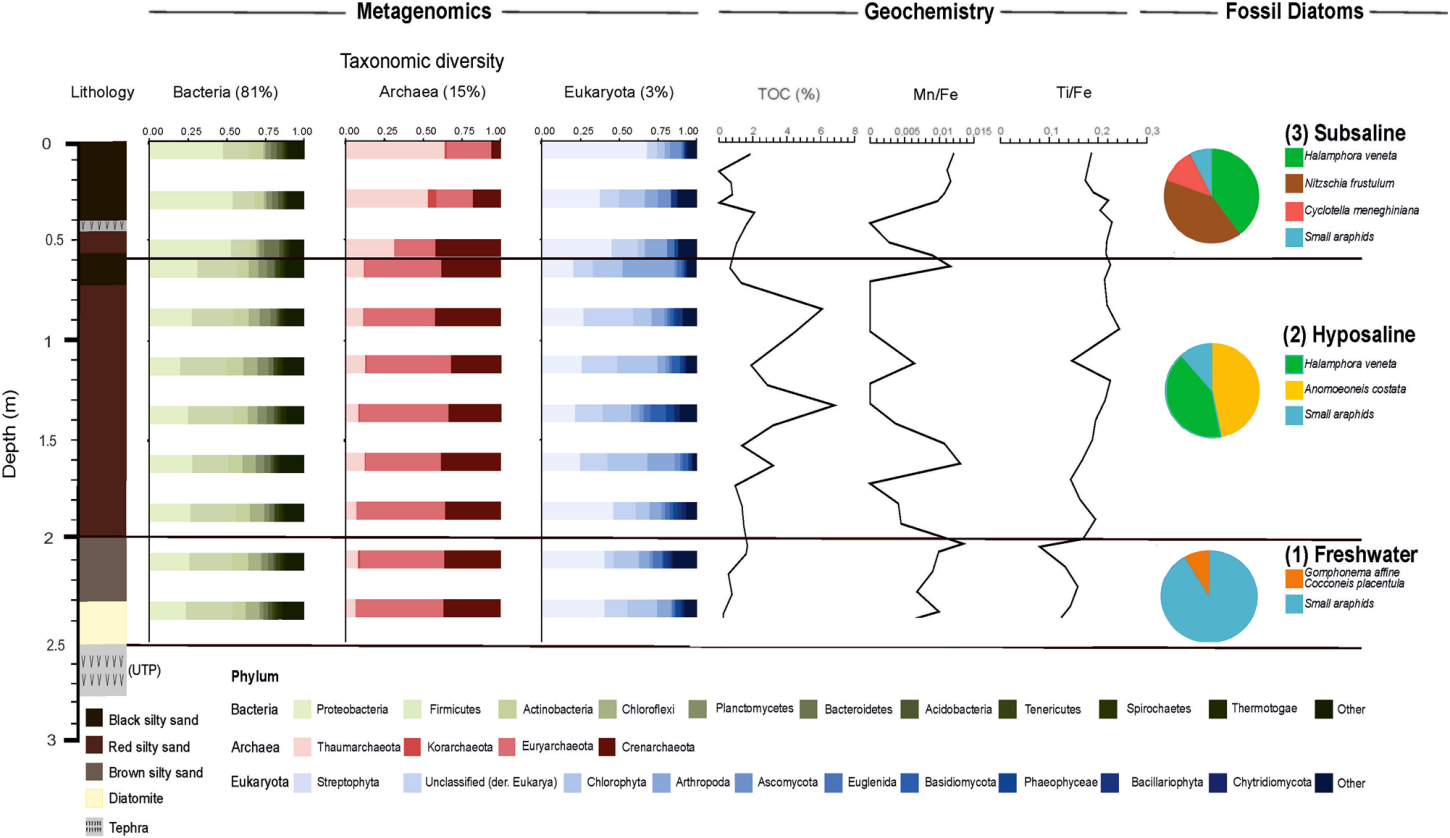
Taxonomic diversity revealed by metagenomic and fossil diatom analysis, and geochemical variables from the Lake Chalco Holocene sediment sequence. Each horizontal bar represents a collected sample, with the exception of the upper row, which shows the average of surface samples S1 (0 cm, i.e., modern) and S2 (0 cm, i.e., modern) (Supplementary Table S1). The lithology of the 270-cm sediment sequence is shown in the first column. The Upper Toluca Pumice (UTP) is represented as tephra underneath the first column (from left to right). Taxonomic diversity is depicted as the relative abundance of phyla Bacteria (green), Archaea (pink), and Eukarya (blue) (columns 2–4). Percent values correspond to the diversity of peptide sequences corresponding to each domain. Geochemical variables related to biological processes and past conditions are shown in columns 5–7. Results of fossil diatom analysis are shown in column 8. Dark horizontal lines show the boundaries for each delimited paleoenvironmental zone: (1) freshwater, (2) hyposaline and (3) subsaline. Edited in CorelDRAW 2020 version 22.0.

The lithology of the studied sediment sequence is as follows: (1) Sediments from 270 to 250 cm are characterized by lapilli, ash and black to light brown silty ashes (massive to stratified); (2) Sediments in the interval 250–235 cm are composed of diatomite (yellow); (3) From 235 to 200 cm, sediments are characterized by massive black to brown sandy silts (brown); (4) From 200 to 70 cm, sandy brown to reddish, banded laminated silts, with scattered or banded pumice fragments (red) are present; (5) Sediments from 70 to 60 cm are black silty sands with organic material; (6) From 60 to 50 cm sediments are sandy brown to reddish, laminated silt, with scattered or banded pumice fragments (red); (7) Sediments from 50 to 40 cm are characterized by lapilli, ash and black to light brown silty ashes, massive to stratified, and (8) uppermost sediments from 40 to 0 cm are black silty sands with organic material (Fig. 3)^36^.

Elemental geochemistry and fossil diatoms in sediment cores can be used as indicators of past wet and dry climate intervals. We measured geochemical indicators including element concentrations and ratios, and Total Organic Carbon (TOC) throughout the core (Supplementary Table S1). Total organic carbon is an important component of sediments and soils and can be used to assess the environmental status of terrestrial and aquatic ecosystems^45^. Maximum TOC values characterize the period of hyposaline conditions. The Mn/Fe ratio, often used to track past O_2_ content in bottom waters and changing redox conditions, was used as a proxy for water-column oxygen concentration^46^. The Mn/Fe ratios display highest values at depths of 200, 150 and 60 cm, suggesting periods of permanent anoxia during warmer conditions and excessive nutrient inputs^47^. The Fe/Ti ratio provides information about fluvial sediment sources. Changes in the abundance of iron oxides can be used to infer fluctuations in inputs of land‐derived detrital material^48^. We observed increasing Fe/Ti ratio values in superficial layers, and highest values at 100 cm. We performed cluster analysis based on Euclidean distance which revealed three groups of samples (Supplementary Fig. S1).

We identified three zones in the Lake Chalco Holocene sediment sequence, based on geochemical analysis and diatom assemblages, which reflect different paleoenvironmental conditions: (1) a cool, freshwater lake (235–210 cm), (2) a warm, hyposaline lake (185–60 cm), and (3) a temperate, subsaline lake (50–0 cm) (Figs. 3, 5, Supplementary Fig. S1, Table S1).

Fossil diatom assemblages provide information about past environmental changes and water quality^7,49^. Fossil diatom analysis, along with knowledge of species ecological preferences, enables inference of past limnological variables such as temperature, salinity, pH, electrical conductivity, and phosphorus concentration^7^. Inferences from our fossil diatom record concur with an earlier diatom-based paleoclimate study from Lake Chalco. Our studies revealed that during the last deglacial (~ 19,500–11,500 cal years BP), conditions were colder and much wetter than present. Assemblages are dominated by small araphid diatoms *Gomphonema affine* and *Cocconeis placentula* (Fig. 3). From 11,500 to 4,500 cal years BP, Lake Chalco was characterized by hyposaline conditions, with higher evaporation rates until ~ 6,500 cal years BP. Typical diatoms taxa include *Anomoeoneis costata*, *Halamphora veneta* and small araphids. After ~ 6,500 cal years BP, salinity in Lake Chalco declined, mean annual precipitation increased slightly, and summer insolation, seasonality, and evaporation decreased^7^. Assemblages are composed of *H. veneta*, *Nitzschia frustulum*, *Cyclotella meneghiniana* and small araphid diatoms.

### Meta-taxonomic analysis of Prokaryote and Eukaryote diversity

Our metagenomic analysis identified 36,722 OTUs (genera) in the sediments of Lake Chalco. Among those genera, 81% correspond to bacteria (29,818 ± 106 identified to genera [ig]), 15% to Archaea (5,710 ± 118 ig), 3% to Eukarya (1,147 ± 6 ig), and < 1% viruses (33 ± 8 ig) (Fig. 3). Some 206 uncertain DNA sequences belong to unclassified genera. With respect to biodiversity⁠, analysis based on large subunit rRNA (LSU) genes revealed high bacterial lineage diversity (26 phyla), with a high Shannon index (5.21 ± 0.22) (Supplementary Fig. S2). Archaea (4 phyla) displays a Shannon index of 2.92 ± 0.69, whereas the Shannon index value for Eukarya (24 phyla) is 3.65 ± 0.34. The most abundant bacterial phyla are Proteobacteria (31%), Firmicutes (26%), and Actinobacteria (9%) (Fig. 3). Deltaproteobacteria and Gammaproteobacteria are the most abundant classes (40% and 23.7%, respectively). Results related to the phyla Actinobacteria must be interpreted with caution, because several genera have been described as contaminants in the extraction DNA kit^50^, however, in this study we performed negative controls on the extraction and no DNA was amplified. Dominant Archaea phyla are Euryarchaeota (53%), followed by Crenarchaeota (36%), and their abundances decrease in the uppermost sediments. Finally, the Eukarya domain display high abundances of Streptophyta (plants: 37%), Chlorophyta (algae: 16%), Arthropoda (insects and others: 10%), and Ascomycota (fungi: 7.6%) (Fig. 3). A Decontam analysis was performed to identify external contamination in our metagenomic data, and showed that only two OTUs (genera), *Archaeoglobus* and an unclassified genus belonging to Actinobacteria, are significantly related to external contamination (Supplementary Fig. S3). They were therefore removed from subsequent analyses^51^. Additionally, we performed negative (mock) controls during the extraction procedure but did not have a control for the sequencing (Supplementary Fig. S3).

### Functional classification of the predicted proteins and enzymes associated with metabolic pathways

Analysis of coding gene fragments revealed 27,636,243 non-clustered predicted proteins in the 12 sediment core samples analyzed, of which 3,227,398 could be annotated against the National Center for Biotechnology Information (NCBI) non-redundant (NR) protein database. The high number of predicted proteins in our study is an indication of the enormous metabolic diversity within Lake Chalco sediments. This suggests that Chalco’s metagenomes have great potential for exploring biotic processes in detail, and that the lake deposits may possess biotechnology potential, e.g., for investigating oil recovery or biodegradation of pollutants and toxic compounds.

**Figure 4:**
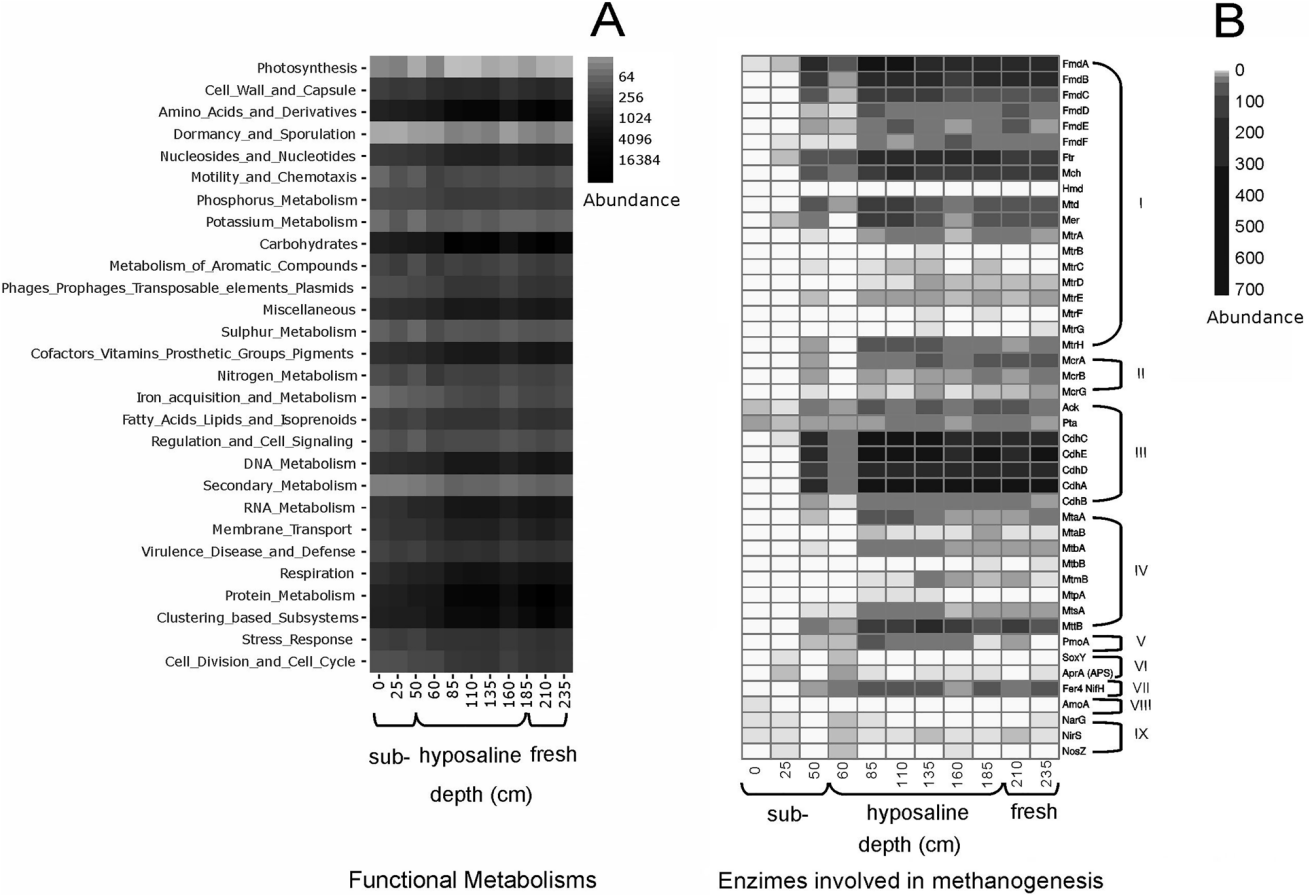
(**A**) Functional classification of the predicted proteins of the Lake Chalco sediment metagenomes, using SEED Subsystems. Samples are ordered from shallowest to deepest samples. Log2 normalization of abundance is shown. (**B**) Enzymes associated with different, presumably active metabolic pathways. Heatmap distribution of the enzymes (or subunits of the enzymes) of: (I) hydrogenotrophic methanogenesis (FmdA-MtrH), (II) shared key enzymes for all variants of methanogenesis (McrA-McrG), (III) acetoclastic methanogenesis (Ack-CdhB), (IV) methylotrophic methanogenesis (MtaA-MttB), (V) aerobic methane oxidation (PmoA), (VI) sulphur metabolism (SoxY-AprA), (VII) nitrogen fixation (NifH), (VIII) nitrification (AmoA) and (IX) denitrification NarG-NosZ), in the different sampled units. The numbers on the sidebar represent the abundance. Edited in CorelDRAW 2020 version 22.0.

Protein analysis revealed 6,144 annotated pfams in the SEED database, including conserved hypothetical proteins. The most abundant SEED hierarchy corresponds to amino acids and derivatives (42%), followed by carbohydrates (6.2%), cofactors, vitamins, prosthetic groups and pigments (5.5%), and DNA metabolism factors (5.0%) (Fig. 4A). The average protein diversity is 3,789 unique pfams per sample, which, according to the Chao1 index, is close to the expected average of 4,316 proteins per sample (Supplementary Fig. S2). The mean Shannon diversity (Hʹ) index for unique proteins is 7.05, i.e., higher than for the taxonomic value (Hʹ = 5.21) (Supplementary Fig. S2). Our multiproxy study revealed three paleoenvironmental zones (Fig. 3), referred to in the following text as: (1) freshwater, (2) hyposaline and (3) subsaline. The Shannon protein index (Hʹ < 7.0) is low in the freshwater zone (235–210 cm) but increases in the hyposaline zone (185–60 cm) (Hʹ = 7.0), and even more so in the subsaline zone (Hʹ = 7.1) (50–0 cm) (Supplementary Fig. S2).

Our results revealed that 3,818 pfams are shared by all three zones (core in the Venn diagram) and are indicative of basic metabolic functions such as DNA replication, recombination and repair, responses to environmental conditions such as heat and cold shock (a proxy for biological stress response)^52^, desiccation, acidity, oxidative stress, and other metabolic pathways such as phospholipid biosynthesis, siderophores, and protein secretion systems (Supplementary Fig. S4A). This variety of metabolic functions is a strong indicator of environmental change throughout the sediment sequence.

### Zonation of microbial metabolisms corresponds to Holocene environmental conditions

Microbial profiling was described against sediment depth because we did not conduct analysis to differentiate between living and dead cells, and we are aware that using the prokaryotic assemblage as a stratigraphic indicator is challenging and beyond the scope of this contribution. The following results, however, suggest a strong relationship with the three paleoenvironmental zones, based on lithological, geochemical, and fossil diatom evidence.

**Figure 5:**
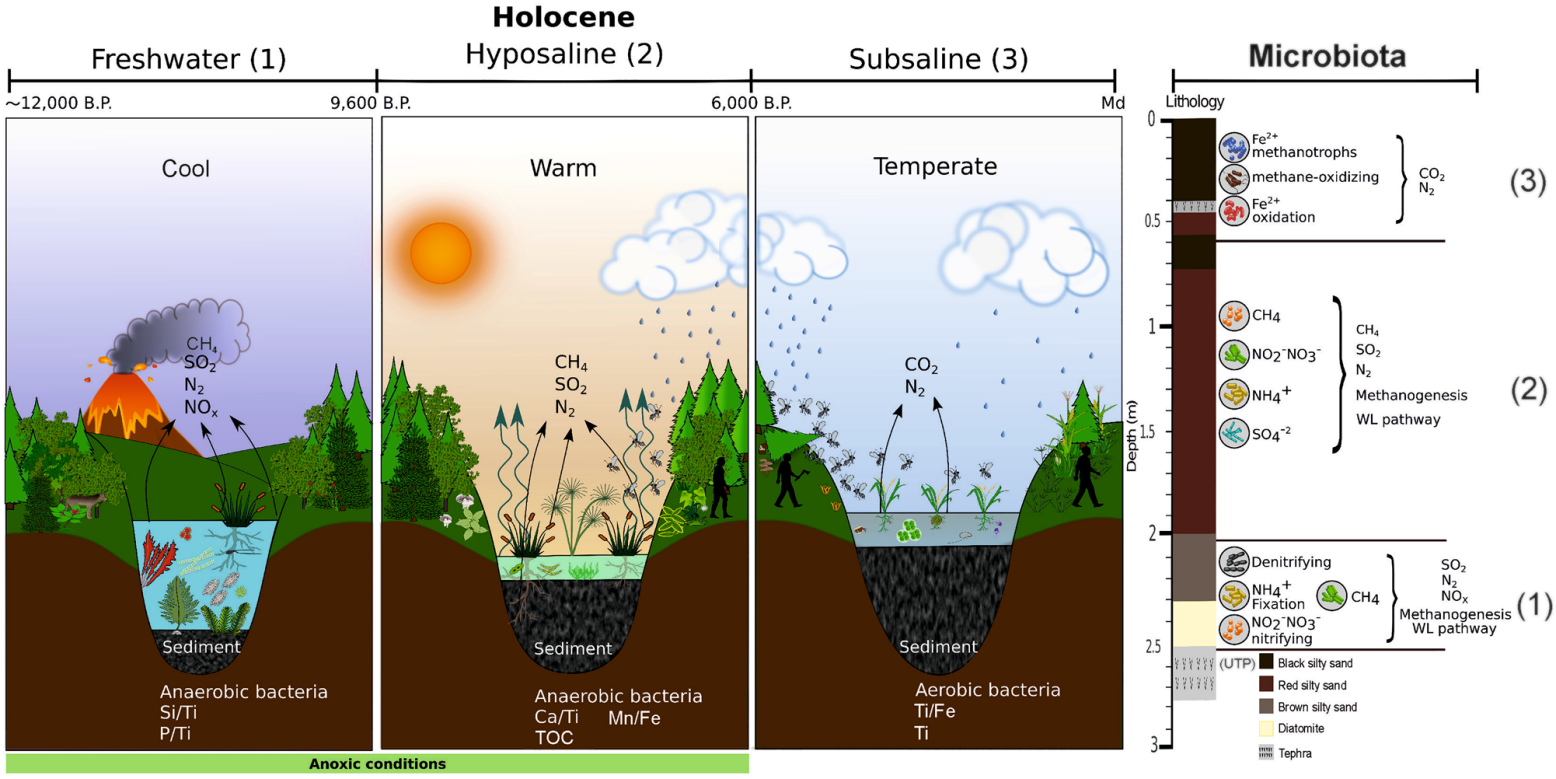
Climate and limnological inferences for the three Holocene zones in the Lake Chalco sediment core: (1) cool, freshwater lake, (2) warm, hyposaline lake, and (3) temperate, subsaline lake, determined using metagenomic, diatom, and geochemical analyses. The horizontal bar represents the time range of each zone, determined during this study and by previous studies cited in the text. The right bar shows the microbiota and enzyme pathways found in each paleoenvironmental zone. Si/Ti and P/Ti values in zone 1 (cool, freshwater lake) suggest high lacustrine productivity. The most abundant Eukarya families (algae) are associated with freshwater conditions. Values of Ca/Ti and TOC in zone 2 (warm, hyposaline lake) suggest periods of intense evaporation and high productivity. Highest TOC values suggest eutrophic conditions. Mn/Fe values in zones 1 and 2 suggest periods of anoxic bottom waters in the lake. Zone 3 (temperate, subsaline) is characterized by a slight increase in lake depth and a decrease in salinity. Geochemical variables Ti and Ti/Fe suggest greater rainfall and runoff. High diversity of fungal pathogens of crops, and highest abundance of Poaceae (grasses), suggest environmental perturbation, probably related to human settlement. Image was made in Inkscape 1.0.

The phylum Firmicutes (Fig. 3), including Gram-positive anaerobes and extremophiles, is dominant in deeper sediments of the core (235–210 cm, Fig. 5). Within Proteobacteria, the most abundant and diverse class is Deltaproteobacteria, followed by the Gamma-, Alpha-, Beta- and Epsilonproteobacteria. We found only 1 member of the Zetaproteobacteria. Deltaproteobacteria *Geobacter* and *Pelobacter* are the most abundant in our data set. They are important organisms of the sulfate-reducing community and are involved in redox of organic and metallic compounds^53^.

We found the highest abundances of Deltaproteobacteria in the freshwater zone (235–210 cm). High abundances of Deltaproteobacteria have been reported in algae-dominated lake sediments and indicate mesotrophic conditions. Consequently, Deltaproteobacteria could be an important bacterial class for nutrient release in sediments, thereby contributing to algae blooms^54^. Among Archaea, the most abundant phyla in the hyposaline zone are Crenarchaeota and Euryarchaeota (Fig. 3). We encountered high abundances of Methanosarcinales, Methanobacteriales, Methanococcales (Euryarchaeota), and Sulfolobales, Thermoproteales and Desulfococcales (Crenarcheota), all of them involved in methane production and oxidation, sulfur respiration or fermentation and N2 fixation. We believe that high Si/Ti values in this zone (Supplementary Table S1) are explained by previous accumulation of the Upper Toluca Pumice deposit (UTP, 12,300 years cal BP, rich in SiO_2_), which originated from the Nevado de Toluca Volcano^41^. Unique protein families found in the metagenomes from the freshwater zone account for 166 unique pfams (Supplementary Fig. S4B). They are associated with dormancy and sporulation, nitrogen metabolism, and hydrogenase genes, and seem to be the result of higher concentrations of hydrogen in successively deeper biogeochemical zones^55^. Hydrogen could serve as one the principal sources of energy. Representation of all identified metabolisms in the pfam results highlights the ecological complexity of the system, as suggested by antibiotic-resistance genes that indicate interactions among plants, fungi, and bacteria^56^. Finally, identification of genes from the entire methanogenesis pathway, and its coenzymes, provides evidence of possible biological methanogenesis and high productivity (Fig. 4B, Supplementary Fig. S5).

From 185 to 60 cm depth (hyposaline), the most abundant bacteria phyla are Firmicutes and Chloroflexi (Fig. 3). The phylum Chloroflexi is one of the most frequently detected phyla in deeper sediments and plays an important role in dissimilatory dehalorespiration^57^. Our data from these lacustrine sediments suggest a high abundance of *Dehalococcoidetes* (Supplementary Fig. S6), which are predicted (homo)acetogens, with the potential to degrade complex organic compounds (Supplementary Fig. S5B)^53^. We also identified anaerobic Gram-positive bacteria and *Ammonifex* that are associated with the nitrogen cycle (Supplementary Table S2), and specifically with N_2_ fixation, ammonification, nitrification, and denitrification^14^. Gammaproteobacteria are highly abundant in deeper and anoxic environments^52^. High S and TOC concentrations in sediments (Fig. 5, Supplementary Fig. S1) support the inference for hyposaline conditions and periods of water column anoxia, with high sulfur, carbon, and nitrogen, which favored nitrogen fixation and sulfur-reducing bacteria such as *Desulfatibacillum*, *Desulfococcus* and *Desulfovibrio,* the latter of which could be related to clastic contribution to the sediments. Although Archaea represents only 15% of the total of the recovered sequences, we found the maximum richness and diversity in this zone (Supplementary Fig. S2), indicating that environmental conditions were optimal for Archaea and extremophile bacteria, which tolerate elevated temperatures, high salinities and anaerobiosis. Higher summer insolation during the early Holocene could have favored the presence of a high-salinity hotspot that was not detected in previous paleoenvironmental studies at this site^32^. Furthermore, 13 genera associated with methane production, belonging to the phylum Euryarchaeota (Supplementary Table S2), were identified. In these sediments, we also encountered hyper-thermophilic Archaea, belonging to the phylum Crenarchaeota (families Fervidicoccaceae and Pyrodictiaceae) (Fig. 3; and Supplementary Table S2). Similar observations were associated with early diagenesis in a sediment sequence from a lake in Argentina that was subject to high evaporation rates^58,59^. The high abundance of genes indicating a “stress response” suggests that microbes in these sediments could have been altered during sampling and storage^36^. We observed the presence of genes encoding enzymes of the Wood-Ljungdahl pathway (WLP) for Archaea (Fig. 4B, I–IV) and Bacteria (Supplementary Fig. S5B), which is an important metabolic pathway in subsurface microbes^60^. The WLP can be used catabolically to achieve redox balance and regenerate NAD+ and oxidized ferredoxin, to increase anaerobic metabolic efficiency^53^. This is the only autotrophic route that fixes carbon and generates energy through formation of ATP simultaneously. The formate hydrogen lyase complex combines NiFe hydrogenase and soluble formate dehydrogenase to couple formate and/or CO-dependent hydrogen production to the generation of the sodium-motive force to generate ATP^53^ (Supplementary Fig. S7).

Changes in water level, as well as higher temperatures and salinity in the hyposaline zone, favored blooms of cyanobacteria^61,62^. In addition, high TOC concentrations in lake sediments suggest higher trophic state^45^. Bacteria and Archaea communities in the freshwater and hyposaline zones suggest periods of anoxia in the bottom waters of Lake Chalco (Fig. 5; Supplementary Table S1). Even though the results of our study did not differentiate between modern and ancient microbial DNA, results from the deepest sediments of the studied core suggest the presence of ancient genetic material, by virtue of the low presence of strictly aerobic bacteria such as *Sphaerobacter*, photosynthetic bacteria *Chlorobium*, and the photosynthetic-chemoautotroph *Roseiflexus*. Presence of aDNA is also suggested by abundant aerobic, photosynthetic, nitrogen-fixing cyanobacteria in the deepest sediments of the freshwater zone (25%) and from the hyposaline zone (56%) (Supplementary Fig. S6, Table S3). Results similar to those in our hyposaline zone were observed in a study of the sediments from ancient Lake Ohrid^49^. They found Gammaproteobacteria co-occurring with cyanobacterial sequences, especially during dry glacial intervals^63^. In addition, we found the highest abundance of Thermoplasmata in sediments from the hyposaline and freshwater zones. Vuillemin et al. reported similar results in Holocene sediments from lake Potrok Aike, Argentina^59^. Additionally, we evaluated the photosynthetic genes along the sediment core (Supplementary Fig. S8), using the Blastp against MetaProt database, which is a manually curated proteomics meta-classification^64^. We observed high abundances of genes encoding for enzymes involved in the photosynthesis pathway in the hyposaline zone (63%), followed by the freshwater zone with 32%, and finally topmost sediments of the subsaline zone, with 5%. Those genes are probably related to anoxygenic photosynthesis or to microbial aDNA. Our geochemical data indicate periods of anoxia during the freshwater and hyposaline zones, suggesting that anoxygenic photosynthesis could have taken place. In that process, H_2_S serves as the primary energy source, whereas phototrophic organisms use light and chemotrophic organisms use chemical compounds as energy resources, the latter being prevalent in dark environments^65^. We also identified fermentative bacteria in these sediments. Substrate-level phosphorylation is most significant in anaerobes and ATP yields are low. Consequently, biomass production is low and intermediate fermentation products for downstream metabolism, used by other microbes, are high^65^. Some metabolic pathways were revealed by pfam abundance. Nitrogen-metabolism genes display high abundance in the hyposaline zone, as does the archaeosine gene, a derivative of 7-deazaguanosine synthesis found in transfer RNAs, which is exclusive to the archaeal phylogenetic lineage^66^ (Supplementary Table S4). This is consistent with the high abundance of Archaea obtained from the taxonomic data. Additional sequences analyzed in this zone indicate the presence of other metabolic pathways, such as aerobic methane oxidation and nitrogen metabolism, specifically nitrogen fixation (Fig. 4, Supplementary Fig. S5). Furthermore, genes encoding antibiotic resistance reach highest abundance in these same zones (freshwater, hyposaline), before decreasing in the overlying, uppermost zone (subsaline) (Supplementary Fig. S9). The high genetic diversity of beta-lactamase genes in deeper sediments supports the idea that beta-lactam antibiotic production is common in microbes and occurred even before human settlement^67^.

In the uppermost sediments (subsaline conditions), the most abundant bacterial phylum is Proteobacteria, with highly abundant (29) genera, including several genera that could be pathogenic to humans, other mammals, and plants (Supplementary Table S2). We observed high abundances of Alpha- and Beta-Proteobacteria in the surficial sediments, with values of 20% and 11%, respectively. Nevertheless, the data must be interpreted with caution because several genera of Proteobacteria could have originated from contamination. Furthermore, we found the Archaea phylum Thaumarchaeota (39%), which plays an important role in global nitrification^68^ and it has been related to human-contaminated environments^69^. We found abundant Bacteria and Archaea that are involved in the nitrogen cycle and could be related to pesticide use in agriculture. The microbes found in the subsurface biosphere seem to represent descendants of surface communities that were buried in the past''^70^.

Finally, we determined the highest abundance and diversity of microbial communities in the hyposaline zone. This could be associated with warmer paleoenvironmental conditions, sediment geochemistry, organic substrates, and surface sediments. Such observations are similar to a previous study in Laguna Potrok Aike, Argentina^59^. The authors of that study proposed that variations in climate conditions and related changes in the depositional environment, led to the development of different microbial communities in the corresponding sediment intervals^59^. Therefore, we suggest that our microbial composition also reflects aspects of burial and early diagenesis.

### Biological characteristics and environmental conditions of the three Holocene zones in Lake Chalco

The three identified zones, based on geochemical and diatom data in the core, match with the biodiversity metagenomics analysis (Supplementary Fig. S1). For instance, analysis of taxonomic and pfam data, using Bray–Curtis dissimilarity, clustered three groups that represent the paleoenvironmental phases, i.e., freshwater, hyposaline and subsaline. In addition, we performed a non-metric multidimensional scaling (NMDS) analysis to evaluate associations between samples and geochemical patterns. The NMDS also revealed three groups that corresponded to the previously defined zones. This association was observed whether we considered (1) all three domains, (2) only Bacteria and Archaea, or (3) Eukarya alone (Supplementary Fig. S1). There is a strong association between Ti/Fe and sediment samples from the subsaline zone, whereas TOC is strongly related to samples from the hyposaline zone. Mn/Fe is most strongly related to deeper sediments from the freshwater zone and with the 185-cm (deepest) sample of the hyposaline zone (Supplementary Fig. S1). Similar groups of samples were observed in the cluster and NMDS analyses (Supplementary Fig. S1). We did, however, encounter two exceptions. The sample from a depth of 60 cm displays a stronger association with the subsaline zone than to the hyposaline, and the sample from 185 cm shows greater affinity with the freshwater zone than with the hyposaline zone. Both samples may reflect transitional periods.

Metagenomic and traditional paleolimnological evidence suggest that during zone 1 (freshwater) Lake Chalco was deep, cool, fresh, and mesotrophic, with anoxic bottom waters (Fig. 5). The early Holocene in Central Mexico was characterized by gradually decreasing runoff^32^, which probably promoted the high biological productivity in zone 1, which was dominated by Bacteria (Supplementary Fig. S1). High biodiversity and mean abundances of cyanobacteria within this zone indicate mesotrophic conditions (Supplementary Fig. S10 and Table S3) and may be evidence of aDNA. We found highest abundances of cyanobacteria in the hyposaline zone, in contrast to a previous study that reported lower abundances with greater sediment depths^71^. Our results might have captured evidence of intense algae blooms in the water column during warmer conditions. We identified the presence of *Synechococcus* in this zone, which is associated with the Gamma-glutamyl phosphate reductase gene (Supplementary Table S3). Other studies have reported this genus in deep sediments as well^71^. We found high biodiversity, but low representation of taxa from Archaea and Eukarya. Analysis of smear slides revealed the presence of an araphid fossil diatom assemblage, indicating cool, fresh waters. Nutrient recycling and moderate lake primary productivity are indicated by high P/Ti and Si/Ti ratios^72^. Among Eukarya, Poaceae (grasses) were the most prominent taxonomic group around the lake. The algae families Zygnemataceae and Pyrenomonadaceae dominate among aquatic eukaryotes (Supplementary Fig. S10). Zygnemataceae spores were reported previously from Late Pleistocene (~ 14,500–10,000 cal years BP) sediments in Lake Chalco^34^. That study inferred cooler temperatures and greater moisture than today, which coincided with intense volcanic activity. Late Pleistocene cool conditions are supported by the presence of Pyrenomonadaceae. Blooms of some algal species from this family can cause red color in the chemocline of cool, meromictic lakes^73^. The Blastp against MetaProt analysis of cyanobacteria revealed that diversity in this zone is second only to that in the hyposaline zone. Surprisingly, the gen Alpha-glucoside transport system permease protein AglG exhibits highest abundance (115 reads) in this freshwater zone. This transporter system could be related with the cellulose polysaccharide, given the forest cover, or with fermentation processes (Supplementary Table S3)^74^. Additionally, we found the presence of mycorrhizal fungal genera (*Arthroderma*, *Rozella*, *Hygrocybe*, *Laccaria*, *Hyaloraphidium*, and *Physarum*), which are associated with mature forests that are characterized by shaded conditions, high humidity, and abundant leaf litter (Supplementary Fig. S10). Metagenomic analysis revealed 12 unique Eukarya genera, including the algal genus *Trachelomonas*, and the primitive tubeworm *Tubulanus* (Supplementary Table S5). Within the Eukarya results, we obtained evidence to support inferences for past environmental conditions in Lake Chalco during the early Holocene. The alpha glucoside transport could be associated with degradation of cellulose from plants or with a fermentation process^74^. The aDNA capture method, directed at target species, may be a good approach to enrich specific aDNA in these sediments^75^.

During zone 2 (hyposaline), Lake Chalco was shallow, warm, hyposaline, and eutrophic, with anoxic bottom waters. High biological productivity in Lake Chalco continued, and perhaps increased in this zone dominated by Bacteria. Here we encountered the most abundant cyanobacteria reads, indicating eutrophic lake conditions (Supplementary Fig. S6 and Table S3). We also detected the presence of *Hyaloraphidium,* a planktonic fungus that is related to eutrophic freshwater. Geochemical variables S and TOC correlate (Supplementary Fig. S1), suggesting a higher trophic state during the warm hyposaline zone. Warm, hyposaline conditions are suggested by the presence of fossil diatoms *Anomoeoneis costata*, *Craticula elkab,* and *Halamphora veneta*^76^. We identified 32 unique Eukarya genera, among them 16 Streptophyta, 5 Chlorophyta, and some fungi and nematodes (Supplementary Table S5). The most abundant families are Dictyosteliida (protists, 33%), Culicidae (mosquitoes, 27%), and Poaceae (22%) (Supplementary Fig. S10). In addition, we observed a high diversity of fungal genera related to tropical regions, such as *Agricus*, *Moelleriella,* and to mature forests, such as *Laccaria*, *Tulasnella*, *Thricholoma*, *Dacrymyces.* We also identified *Furculomyces,* a symbiont that lives in the gut of an aquatic Chironomidae (midge), and *Smittium and Arthroderma*, fungal pathogens of mosquitoes and keratin, respectively (Supplementary Fig. S10). We infer that a mature forest existed around Lake Chalco during this period. Presence of aDNA in sediments is also suggested by abundant aerobic, photosynthetic, nitrogen-fixing cyanobacteria that probably lived in surface waters. We observed aDNA abundances of 30% and 60% in the freshwater and hyposaline zones, respectively (Supplementary Table S3). A total of 527 unique pfams are found in this zone, displaying subsystems like cell signaling, virulence, defense, iron acquisition, and “phages, plasmids and transposable elements” subsystems, and nitrogen metabolism genes related with anoxic and extreme environmental conditions.

The shift from the hyposaline to the subsaline zone occurred ∼ 6,000 cal years BP (60 cm depth). The dry climate around Lake Chalco^7^ turned wet, with greater precipitation and increased runoff, the latter inferred from Ti and Fe^32^, leading to lower lake salinity. High abundances of Eukarya families Poaceae and Culicidae (Supplementary Fig. S10) during this transitional period indicate modification of the surrounding landscape by humans, which increased cultural eutrophication of Lake Chalco (Fig. 5, Supplementary Fig. S10). Our results show that early human activities had a large and rapid impact on aquatic and terrestrial ecosystems in Mesoamerica. Chalco changed to a shallow, temperate, subsaline, eutrophic lake after ca. 6,000 cal years BP (subsaline, Fig. 5). Subsaline conditions were revealed by the presence of fossil diatoms *Cocconeis placentula* and *Halamphora veneta*. The NMDS of geochemical variables shows an association with the ratio Ti/Fe (Supplementary Fig. S1). These elements are related to inputs of clastic sediments, mineral particles, and/or rock fragments that can be transported to the lake by wind or fluvial processes. Previous studies suggested profound changes in Lake Chalco since ~ 5,000 cal years BP^76^. Iron is considered a potentially harmful element (PHE), which may be indicative of human-mediated contamination. For instance, high Fe concentrations in surficial sediments could be related to inputs of clastic sediments, and often reflect agricultural activities^77^. We determined 13 plant genera, five belonging to the family Poaceae (58%), including the genus *Zea* (corn), known to have been cultivated and consumed by early settlers^78^, and *Oryza*, a fast-growing weed that is indicative of human-mediated habitat disturbance. Twelve microscopic fungal genera were observed, including taxa that are pathogenic on wheat and rice (*Gibberella* and *Cladochytrium*), plants, keratin, and flies (Supplementary Fig. S10)^79^, and a protozoan that is pathogenic in humans (*Giardia*). We found high abundances of the family Culicidae (18%) (Supplementary Fig. S10) during the period of human occupation. The subsaline zone displays 290 unique pfams and the highest number of representatives of potassium metabolism throughout the entire Holocene (Supplementary Fig. S4B). The abundance of genes related to Cyanobacteria in this zone is much lower (9%), in contrast with findings from the Blastp against MetaProt database of the freshwater (30%) and hyposaline zones (60%) (Supplementary Fig. S10, Table S3).

Our findings suggest that the biota in and around Lake Chalco during the Holocene responded mainly to changes in temperature, salinity, and trophic state, reflecting climate and human impacts over the last 6,000 years. This implies landscape modifications, agricultural activities and accelerated lake eutrophication. Furthermore, prokaryotic assemblages revealed gradual deposition of microbial communities capable of anaerobic fermentation of organic material and methanogenesis, as well as evidence for volcanic activity, inferred from the metabolic potential for sulfur cycling in the deeper zones (hyposaline and freshwater). This study generated information on Neotropical Prokaryote and Eukaryote diversity and microbial metabolic pathways during the Holocene. Nevertheless, we recommend that future studies focus on detailed characterization of microbial substrates and constrain post-depositional processes. Such studies should also consider selection processes that result from gradual depletion of substrates during burial^52,59,63^. Finally, we highlight the importance of including additional geochemical measures, such as porewater chemistry^80^. and sedimentology^81^ in future studies and measuring biomass by qPCR assay.

## Conclusions

Metagenomics is a powerful complement to traditional paleolimnological approaches and contributes to better understanding the historical ecology of aquatic and terrestrial ecosystems, and biotic processes in the Neotropical-Nearctic transition zone. Microbes found in the subsurface biosphere represent descendents of surface communities that were buried in the past. Metagenomics proved suitable to describe Holocene biological diversity, environmental conditions (salinity, temperature, and trophic state and human activities) in and around high-altitude, tropical Lake Chalco. Challenges involved with using this novel metagenomic approach for study of lacustrine ecosystems include acquisition of good-quality DNA from sed(a)DNA, and taxonomic gaps in available genomic databases, especially from high-diversity aquatic ecosystems in the tropics, like Lake Chalco. Among paleoenvironmental proxies, Fungi was one of the most sensitive bioindicators of past vegetation, i.e. of mature tropical forest, and later, crops, in near-surface sediments. Our study illustrates how new genomics techniques can shed light on the history of biota in and around aquatic ecosystems, especially in the poorly studied Neotropics.

## Methods

We present the workflow schematically (Fig. 2).

### Sampling the sediment sequence from Lake Chalco, Mexico

A field laboratory was constructed, and all implements, and surfaces were washed with 1:1 water-hypochlorite solution and Lysol to sterilize. Personnel working in the laboratory used sterilized gloves, protection overalls and mouth covers. A 270-cm-long sediment core was retrieved from the former lacustrine area of Lake Chalco, using a piston corer. Sediment was recovered in 1-m-long stainless-steel tubes that were washed with cleaning solution before being brought into the laboratory. A piston system was used to extrude the sediment, which was transferred into PVC half-tubes that had been cut lengthwise and washed with a cleaning solution. Sediment samples were collected from the center of the core, using sterile and nuclease-free Falcon tubes (50 ml), starting at the top and continuing downward. Twelve samples were collected at depths along the core length (Supplementary Table S1). The two topmost samples are considered modern. After collection, samples were frozen in the field and stored in liquid nitrogen at − 80 °C. A second 250-cm-long sediment sequence was collected from the same lake location for geochemical and diatom analysis. Sediment stratigraphy was identical in both cores, with the Upper Toluca Pumice, which dates to 12,500 cal years BP, present at the base of both sequences.

### Diatom and geochemical analyses

Smear slides from 24 depths were prepared for diatom identification and mounted using Norland Optical Adhesive^82^. Slides were observed using an Olympus BX50 microscope under 600× magnification; 50 diatom valves were counted and identified to species level using standard literature^83,84^⁠. Identified species were compared to the diatom assemblages reported previously^33^⁠ and the assemblage in each sample was classified as freshwater, halophilous (hyposaline) or alkaliphilus (subsaline). The same environmental succession reported by Caballero and Ortega (1998), was identified in our sediment sequence, based on diatom assemblages. The base of the core dates to about 12,500 cal years BP, as indicated by the presence of the UTP (Upper Toluca Pumice), and sediments overlying this stratigraphic marker are rich in freshwater taxa that characterized the cooler deglacial at the end of the Pleistocene.

For geochemistry, the same 24 samples were dried, homogenized using an Agate mortar, and analyzed for C and N content using a Niton XL3. The same samples were also analyzed by X-Ray diffraction using a Niton Handheld XRF analyzer (Niton 950 FXL). Data used were averages of three measurements. Ca/Ti profiles in this core closely matched profiles reported for cores taken previously from Lake Chalco. High Ca/Ti values characterize the Early Holocene and have been interpreted as indicative of authigenic carbonate precipitation during a warmer, saline phase^32^⁠. Ti is associated with allochthonous material. To remove the influence of allochthonous material on changing stratigraphic concentrations of phosphorus, calcium, and silica, the relation between P, Ca and Si, and Ti was calculated. In addition, Fe/Mn, Fe/Ti and TOC were measured and used for comparison with our metagenomic results. Principal Components Analysis (PCA) was used to identify co-variables (data and plots not shown) and we selected the three most informative variables in the sediments related to proxies for productivity (TOC), redox condition (Mn/Fe) and erosion/transport (Ti/Fe).

### Metagenomic DNA extraction

DNA was isolated from the 12 Holocene sediment samples taken from the Lake Chalco core. Each sample was sequenced using two NGS methods (Supplementary Fig. S2). To obtain the amount of DNA required for sequencing, each sediment sample was processed in quadruplicate, with the exception of the four deepest sediment samples that were sampled twelve folds. In each extraction round, we included a negative control (mock) and amplification directed at the 16S rRNA gene to ensure reliable controls. The metagenomic DNA was isolated using the Powersoil DNA Isolation Kit (Mo Bio Laboratories, Inc.). DNA purity and quantity were estimated using a NanoDrop spectrophotometer. DNA integrity was checked in 0.8% agarose gels, and to check DNA quality, a PCR reaction for the 16S rRNA gene (v3-v4) was conducted.

### Metagenome next generation sequencing

Libraries were constructed and sequenced at the Genome Services Laboratory of the Unidad de Genómica Avanzada (UGA, formerly LANGEBIO/CINVESTAV), Irapuato, Mexico. Sample concentration was determined using a Qubit 4 Fluorometer (Thermo Fisher). The first five samples had concentrations (~ 64 ng/µl) suitable for the Illumina TruSeq nano platform, according to the manufacturer's instructions (Illumina, San Diego, CA). The seven deeper samples had lower DNA concentrations (~ 10 ng/µl) and were sequenced using the Illumina Nextera XT platform, according to the manufacturer's instructions (Illumina, San Diego, CA). Most raw reads displayed high quality (Q > 30, 98% of the sequences), and low-quality reads were discarded. The assemblage of metagenomes was carried out by Velvet (Zerbino DR, 2008), using 21- and 31-kmer thresholds, and a minimum coverage of 2×. Metagenomic assembly of the shotgun sequence data of sediment samples showed a total of 1,421,823,631 sequencing reads in 106.6 Gb. For bioinformatic analysis, assembled sequences were submitted for gene coding annotation and taxonomic classification to the MG-RAST server^85^ (Supplementary Fig. S2, Table S1).

### Taxonomic annotation

The assembled metagenomes were annotated (99%) through the metagenomics MG-RAST server^6^. For taxonomic assignment, we used large ribosomal subunit (LSU) databases for reference^86,87^⁠. The positive alignments were selected using BLAST (e-value 1e−10; 90% identity; minimum alignment length of 15 bp; choosing the best hit).

### External contamination detection

In addition to including negative controls during the DNA extraction, we performed a decontamination analysis to identify and remove contaminant sequences from our resultant sequences. Analysis was carried out using the DECONTAM R package^88^. We identified reads that had inverse correlation with the total DNA concentration, which was measured in the NanoDrop. The frequency approach was considered^89^.

### Metagenomic coding genes annotation

Predicted coding genes from the assembly were annotated using the MG-RAST server^5^⁠ and the SEED ontology^90^. Positive alignments were selected using BLASTp (cut-off values used were an e-value of 1e−10, a minimum identity of 90%, and a minimum alignment length of 15 amino acids, and the best hit was chosen). After 90% identity clustering and annotation, it was possible to annotate an average of only 32% of the predicted proteins per sample using the M5NR (novel non-redundant protein) database^91^.

### Biodiversity analysis

Multiple biodiversity indexes (Alpha diversity: Observed, Chao1, Shannon, and Simpson and Beta diversity) were determined for taxonomic and protein family genes (pfams) (Supplementary Fig. S2). We associated metagenomic analysis with environmental conditions using Bray–Curtis and Euclidean dissimilarity analysis to identify changes in biological communities. Ordination plots and heatmaps, developed using cluster and abundance analyses, enabled a better understanding of the distribution of taxonomic groups and genes in the sediments. Finally, quantitative DeSeq2 and qualitative VENN diagrams were used for comparative analysis, and an exhaustive literature search was conducted (Fig. 2).

### Paleoenvironmental inferences

Environmental zones were defined using the major changes in metagenomic (taxonomic diversity, protein families and microbial metabolism), fossil diatom assemblages, and geochemical variables (TOC, Mn/Fe and Ti/Fe ratios) (Fig. 5, Supplementary Table S1), and application of statistical analyses (Cluster Analysis, and non-metric multidimensional scaling (NMDS) analysis) (Supplementary Fig. S1).

### Methanogenesis, other methane production and homoacetogenesis enzymes analysis

A local database was constructed with the metagenomes of the different sample units from this work. An exhaustive homology search was conducted for each enzyme (and for each subunit of multimeric enzymes) of the hydrogenotrophic, acetoclastic and methylotrophic methanogenesis pathways, using HMM profiles that were constructed with HMMER^92^⁠. These profiles were used to scan the local database, following the approach of Muñoz-Velasco et al.^93^. Using this strategy, we also searched the enzymes of the biosynthetic pathways of the main coenzymes involved in biological methane production and the enzymes present in the Wood-Ljungdahl pathway (homoacetogenesis). Cutoff values used in the searches were an identity value > 35%, query coverage of 85% and an e-value < 1 × 10^−6^. Sequences that passed the cutoff were aligned with MUSCLE v3.9.31, using default parameters.

### MetaProt analysis

Our metagenomes were compared by rapid BLASTp against the MetaProt database using DIAMOND^94^ (https://github.com/bbuchfink/diamond) and a frequency table was generated. The frequency table included the sequence name of the MetaProt database, the gene and species, and finally, the count. In total we found 291,539 proteins, with 60% of minimal identity, *p*-value < 1 × e−15 and alignment coverage of 50%. We obtained approximately 234,891 hits in total (https://github.com/williamorsi/MetaProt-database/blob/master/blast_to_gene_matrix.py). After the MetaProt Blast analysis, we built a photosynthesis gene database based on Photosynthesis Reference pathway Kegg (https://www.genome.jp/kegg-bin/show_pathway?map00195)^95^. We searched for photosynthesis- and cyanobacteria-related genes, comparing our results against MetaProt results^96^ (https://data.ub.uni-muenchen.de/183/). Results were analyzed and visualized with R v.3.6.3.

### Statistical analysis

All statistical analyses were conducted using R packages (version 3.4.0, with an X86_64 architecture). Plots were constructed with R’s ggplot2 (v3.1.0)^97^, RcolorBrewer (v1.1-2)^98^. Multivariate analyses of community diversity and its significance were assessed with the PHYLOSEQ (v1.27.2)^99^, VEGAN (v2.5-4) and LIMMA (v3.39.12)^100^⁠ R studio packages (v3.4.0). The Shannon index accounts for the number of individuals and number of taxa, which varies from 0 for communities with only a single taxon, to high values for communities with many taxa, each possessing few individuals^101^. Differential abundance analysis between zones with different salinity conditions was performed using the DeSeq2 package^102^, which estimates log2 fold changes based on the adjustment of a negative binomial distribution, using the Wald test and Bonferroni method for *p*-value calculation and adjustment, respectively. Only taxa with *p*-adjusted values (*p*-adj; corrected for multiple testing) < 0.01 on the Wald test were considered to be different in abundance for taxonomic comparisons and *p*-adj < 1e−3. For protein functional analysis comparisons, we used *p*-adj < 1e−4.

## Supplementary Information

Supplementary Information.

## Data Availability

Sequences from this project are available in the Bioproject accession PRJNA522291, available in the NCBI database. SRA NCBI access: Metagenome: 200_4 (Access number: SRX5450430);200_3 (Access number: SRX5450429);200_2 (Access number: SRX5450428);200_1 (Access number: SRX5450427);100_4 (Access number: SRX5450426);100_3 (Access number: SRX5450425);100_2 (Access number: SRX5450424);100_1 (Access number: SRX5450423);50_2 (Access number: SRX5450422);50_1 (Access number: SRX5450421);S_2 (Access number: SRX5450420);S_1 (Access number: SRX5450419).
