# Supplementary material for: Holocene life and microbiome profiling in ancient tropical Lake Chalco, Mexico
Source: Sci Rep. 2021 Jul 5;11:13848. doi: 10.1038/s41598-021-92981-8 (PMC8257590; doi:10.1038/s41598-021-92981-8)
Supplement: Supplementary file 1 — Supplementary Information. [file 41598_2021_92981_MOESM1_ESM.pdf]

Supplementary material

**Holocene life and microbiome profiling in ancient tropical Lake Chalco, Mexico**

Bárbara Moguel[1,2,6], Liseth Pérez[7], Luis D. Alcaraz[3], Jazmín Blaz[3], Margarita Caballero[4], Israel Muñoz-Velasco[3], Arturo Becerra[3], Juan P. Laclette[5], Beatriz Ortega-Guerrero[4], Claudia S. Romero-Oliva[8], Luis Herrera-Estrella[9,10*], Socorro Lozano-García[1*].

*Corresponding authors:
**Luis Herrera-Estrella** (luis.herrera-estrella@ttu.edu)
Texas Tech University, Lubbock Texas, and Centro de Investigación y de Estudios Avanzados del Instituto Politécnico Nacional (CINVESTAV), Km 9.6 Libramiento Norte Carretera Irapuato-León, C.P. 36821 Irapuato, Guanajuato, México.
**Socorro Lozano-García** (mslozano@unam.mx)
Instituto de Geología, Universidad Nacional Autónoma de México, 04510, Ciudad de México, México.

**Figure S1**. Geochemical and taxonomic analysis of sediment samples based on Euclidean and Bray-Curtis dissimilarities. (A) (Geochemical variables). Top: Cluster Analysis for Euclidean distance obtained from six geochemical variables analyzed for each sample, Bottom: Non-metric multidimensional scaling (NMDS) analysis for all three domains, based on Bray-Curtis dissimilarities. (B) (Taxa). Top: Cluster Analysis for Bray-Curtis dissimilarities from the taxa in each sample, Bottom: Non-metric multidimensional scaling, for Bacteria and Archaea domains analysis, (NMDS) based on the Bray-Curtis dissimilarity. (C) (Protein families): Top: Cluster Analysis for Bray-Curtis dissimilarities from protein families of each sample, Bottom: Non-metric multidimensional scaling (NMDS) for Eukarya analysis based on the Bray-Curtis dissimilarity. Color pattern was based on the age of each sample (see the scale on the left side) and geometric forms represent each identified paleoenvironmental zone (1-3): circles correspond to zone 1 (freshwater); triangles to zone 2 (hyposaline) and squares to zone 3 (subsaline). Note that Cluster Analysis (top panels) grouped some samples (cm) with other zones (e.g., samples from 185, 60 and 50 cm). We attribute this to variable environmental conditions during transitional conditions.

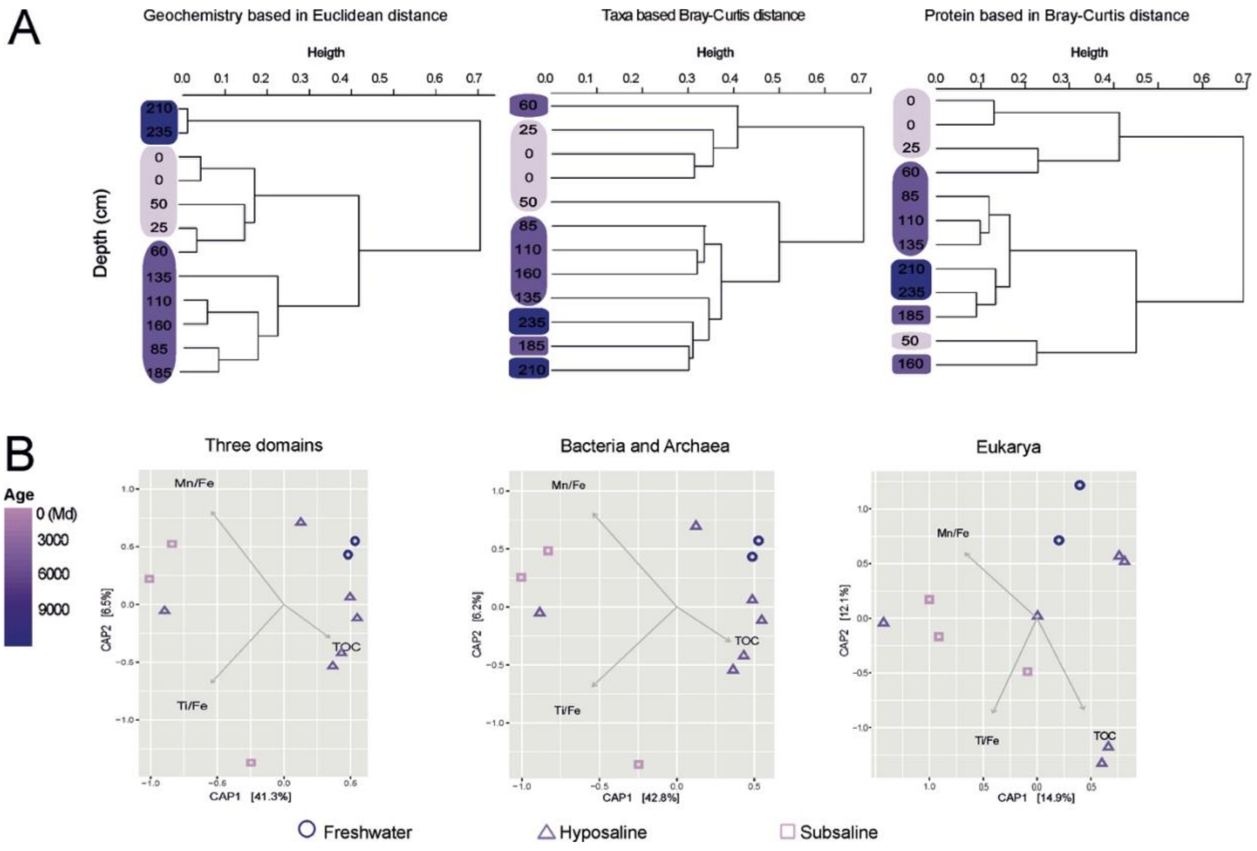

**Figure S2**. Alpha diversity measures of richness and diversity for the annotated taxonomic and predicted proteins of the metagenomes.

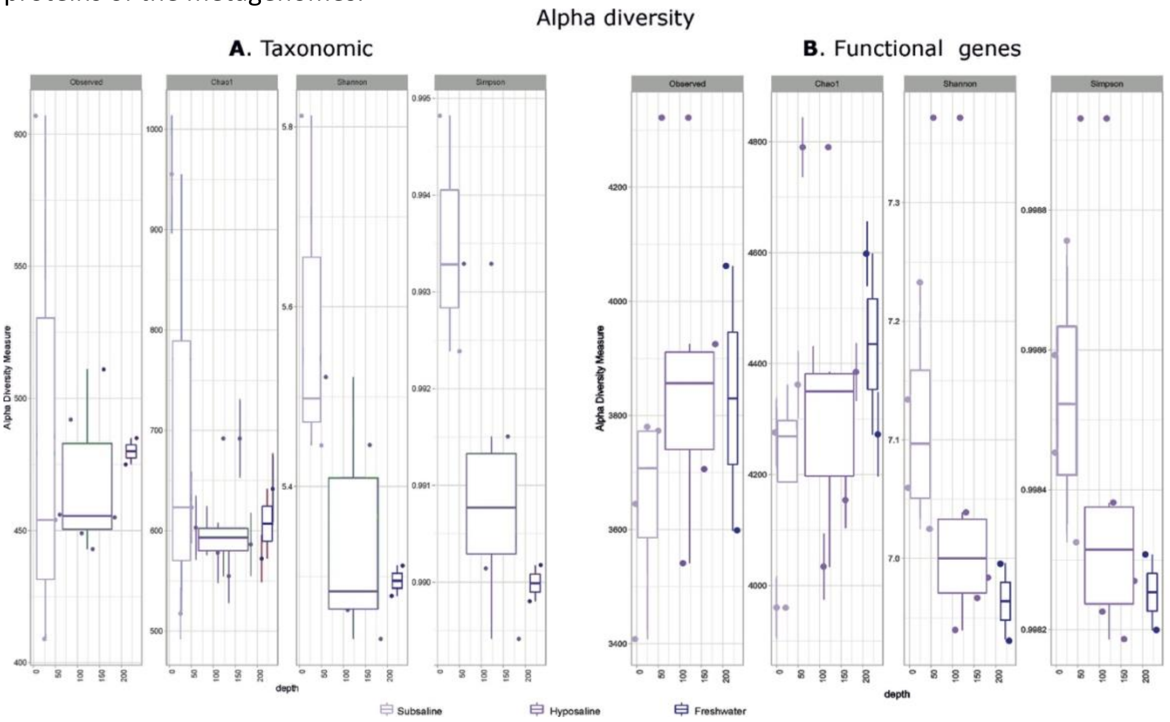


**Figure S3.** Control of external contamination by (A) negative controls during extraction aDNA and (B) decontam analysis during the sequence analysis.

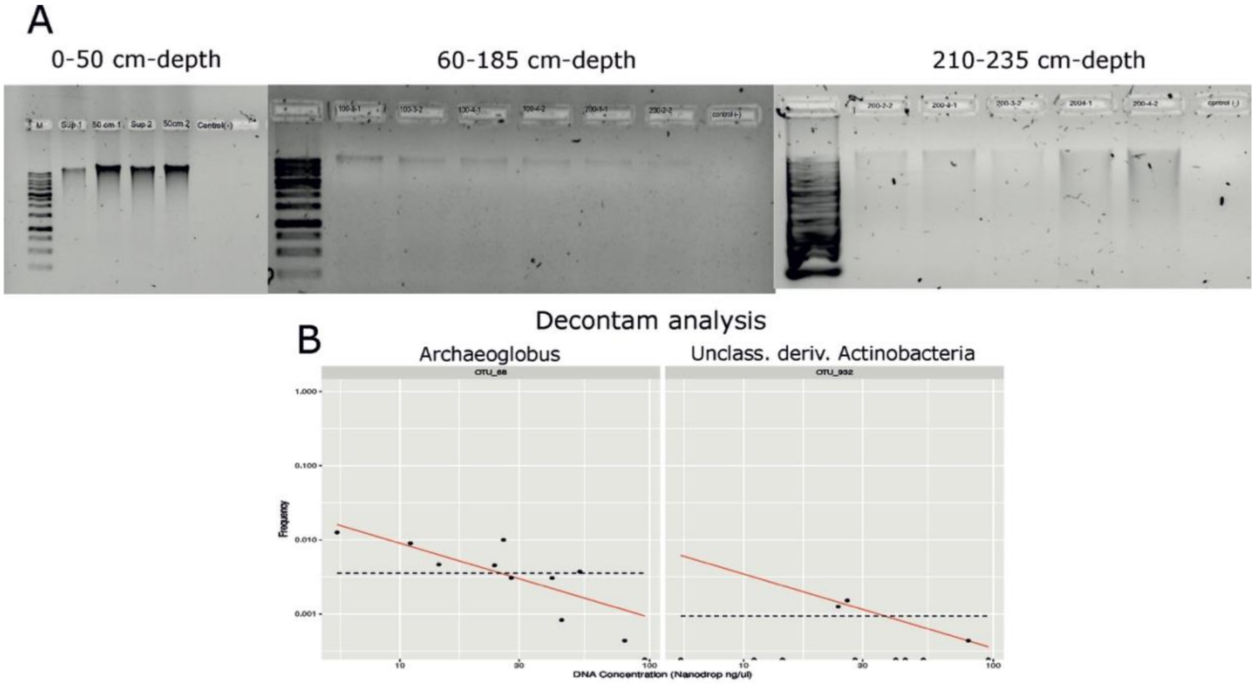

**Figure S4**. Heatmap of the pfams shared among all zones, showing the core of the predicted based on L1 classification (A) and unique pfams for only one or another zone, based on L1 classification (B) of the Chalco sediments based on the Venn diagram analysis

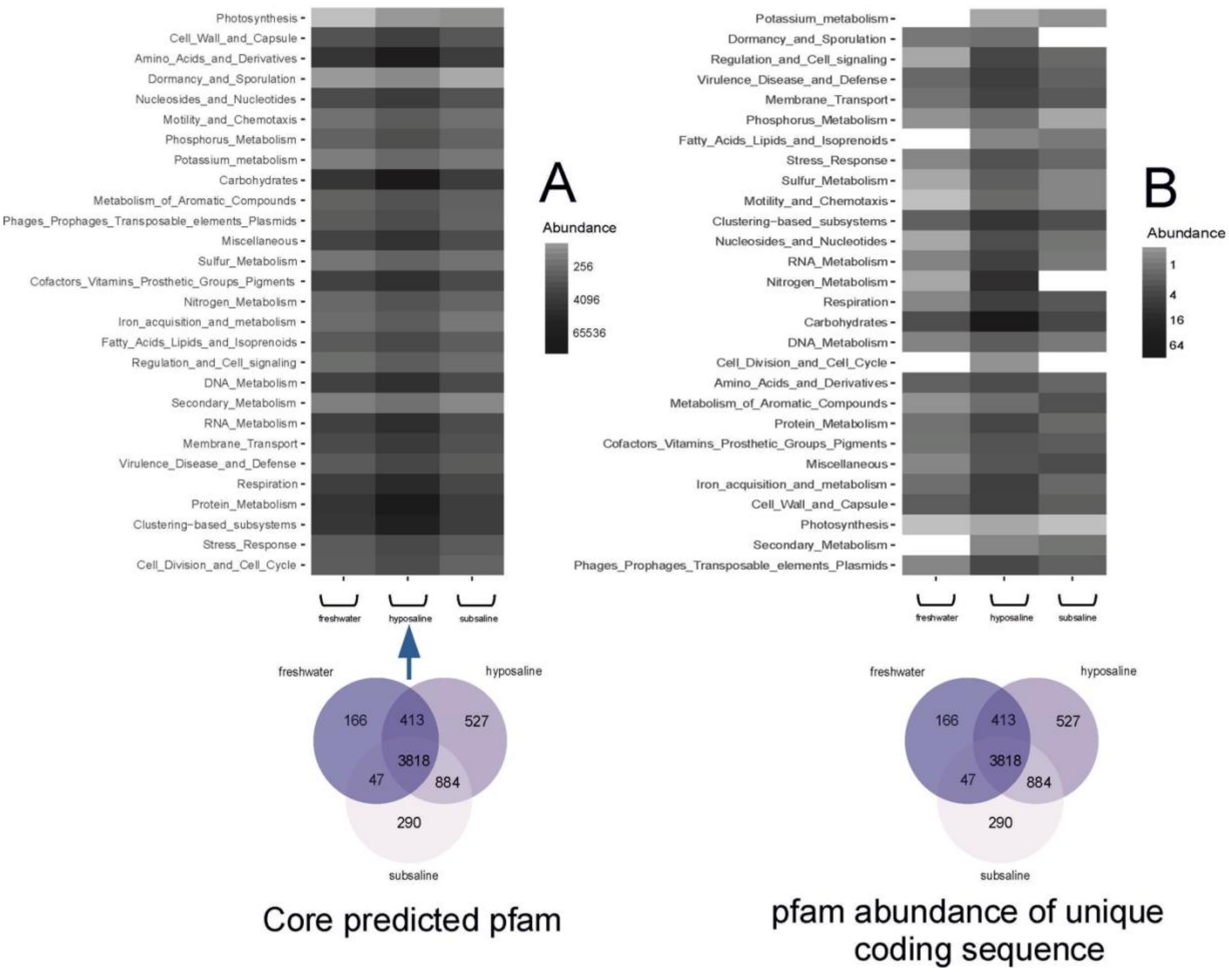

**Figure S5**. Heatmaps show A) Main coenzymes of methanogenesis and B) Enzymes involved in the assimilation of carbon by homoacetogenesis.

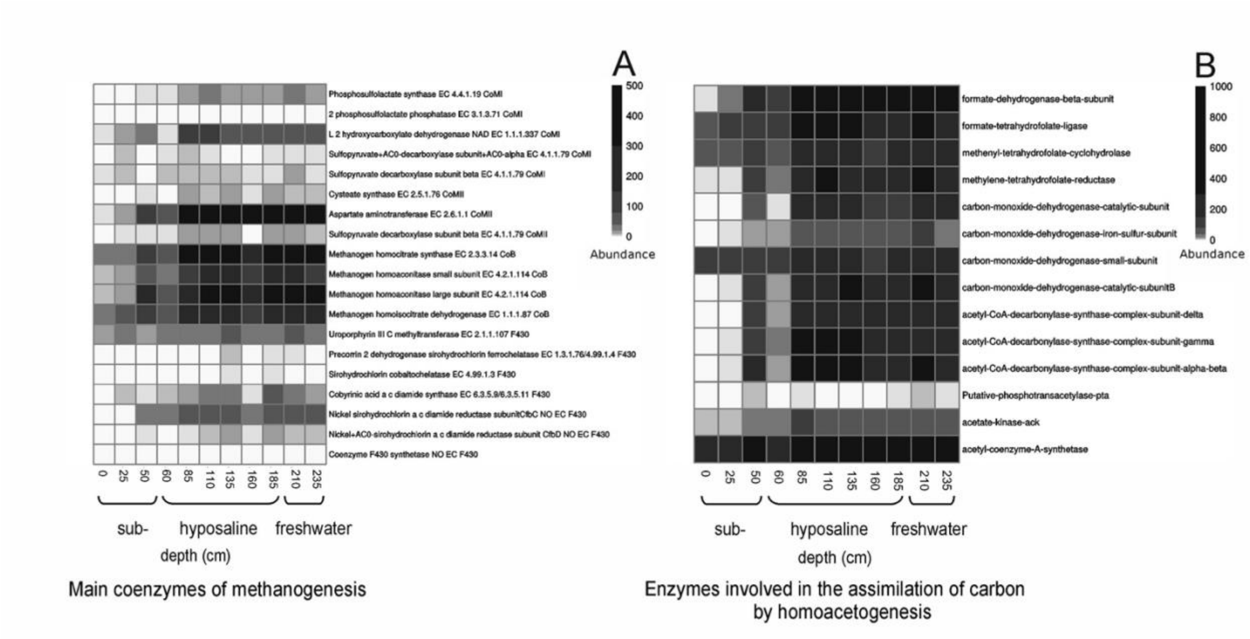

**Figure S6**. (Upper panel) Bar chart shows the top 10 most abundant families withing Bacteria domain, (Bottom panel) the vertical bar chart shows the abundance of Cyanobacteria per Order by each zone and the horizontal bar chart shows the abundance and distribution of the genus of Cyanobacteria across the sedimentary core.

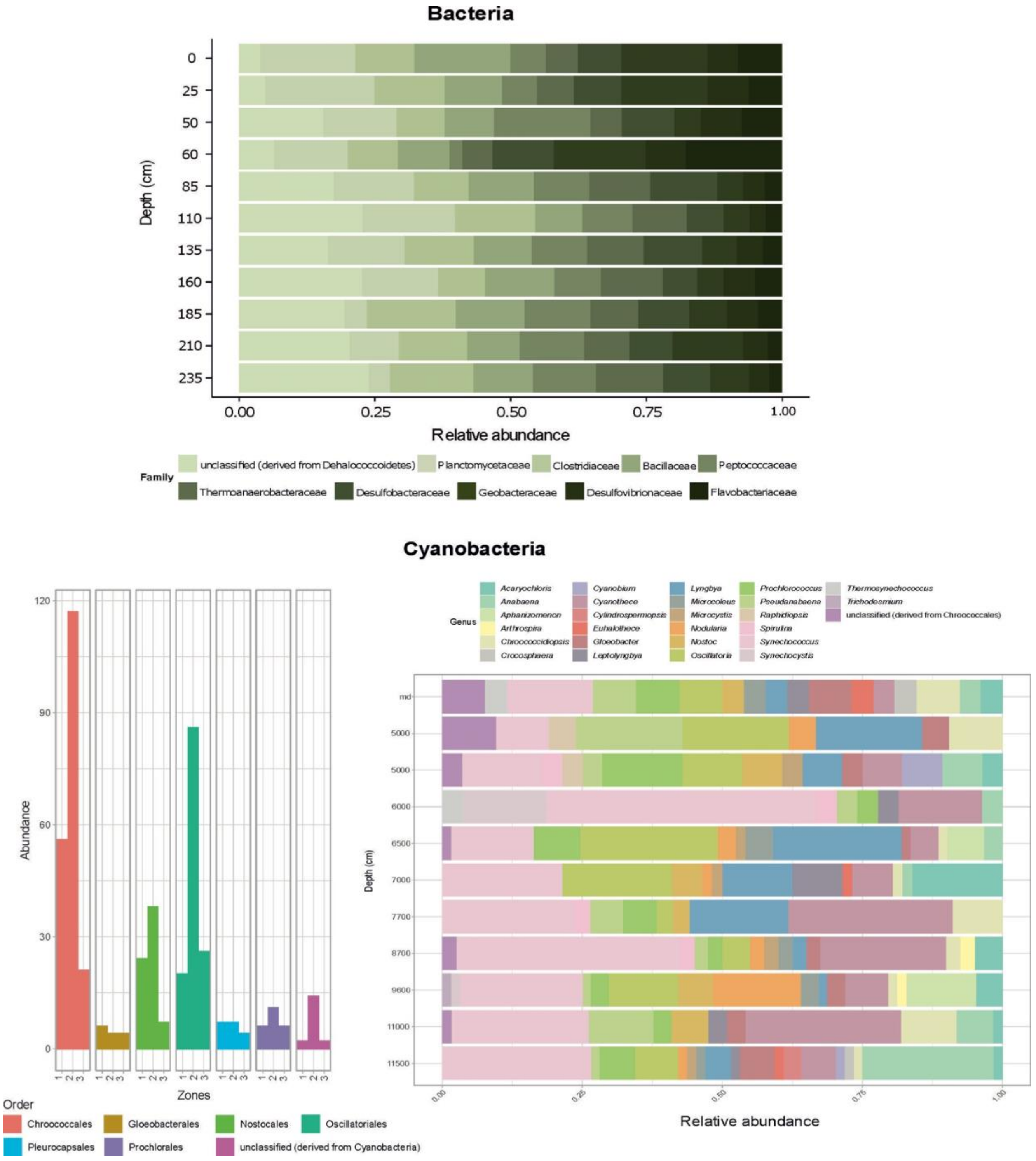

**Figure S7**. Heatmap analysis of the hydrogenases identified throughout the sediment sequence.

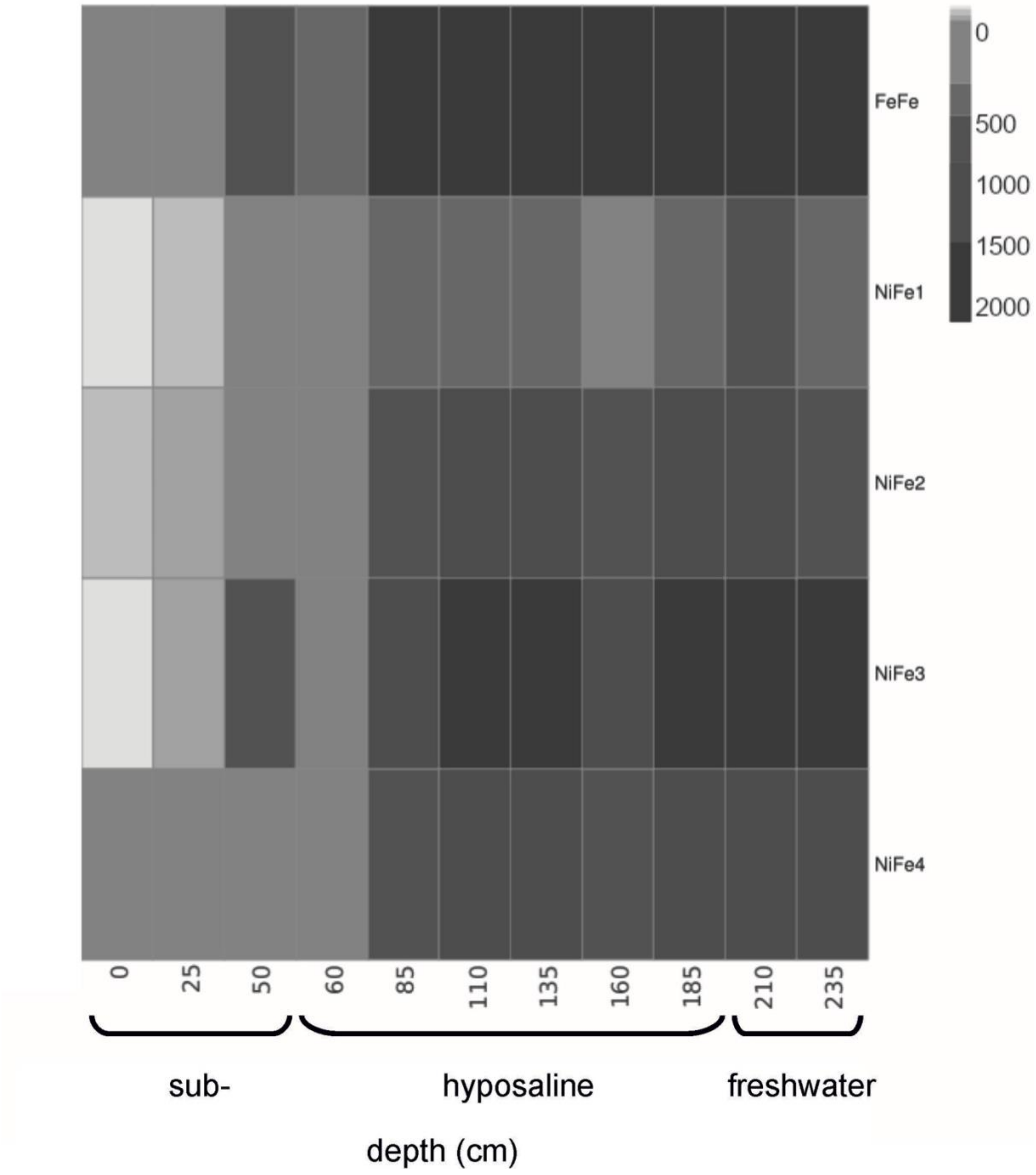

**Figure S8**. Heatmap analysis of the photosynthetic pathway enzymes throughout the sediment sequence, based on the Kegg photosynthesis pathway (Kanehisa, M., 2019).

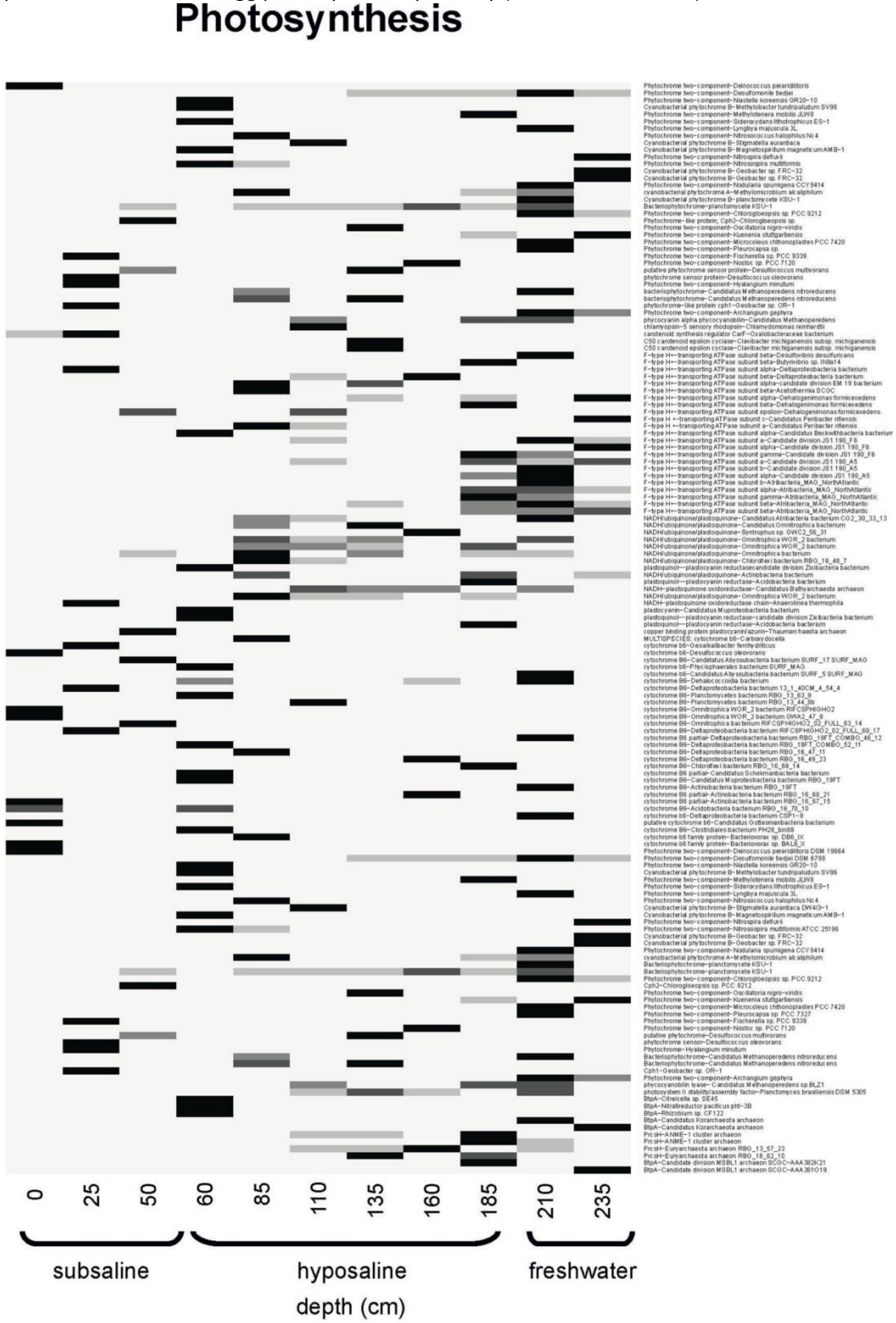

**Figure S9**. Heatmap analysis of the resistance antibiotics identified throughout the sediment sequence.

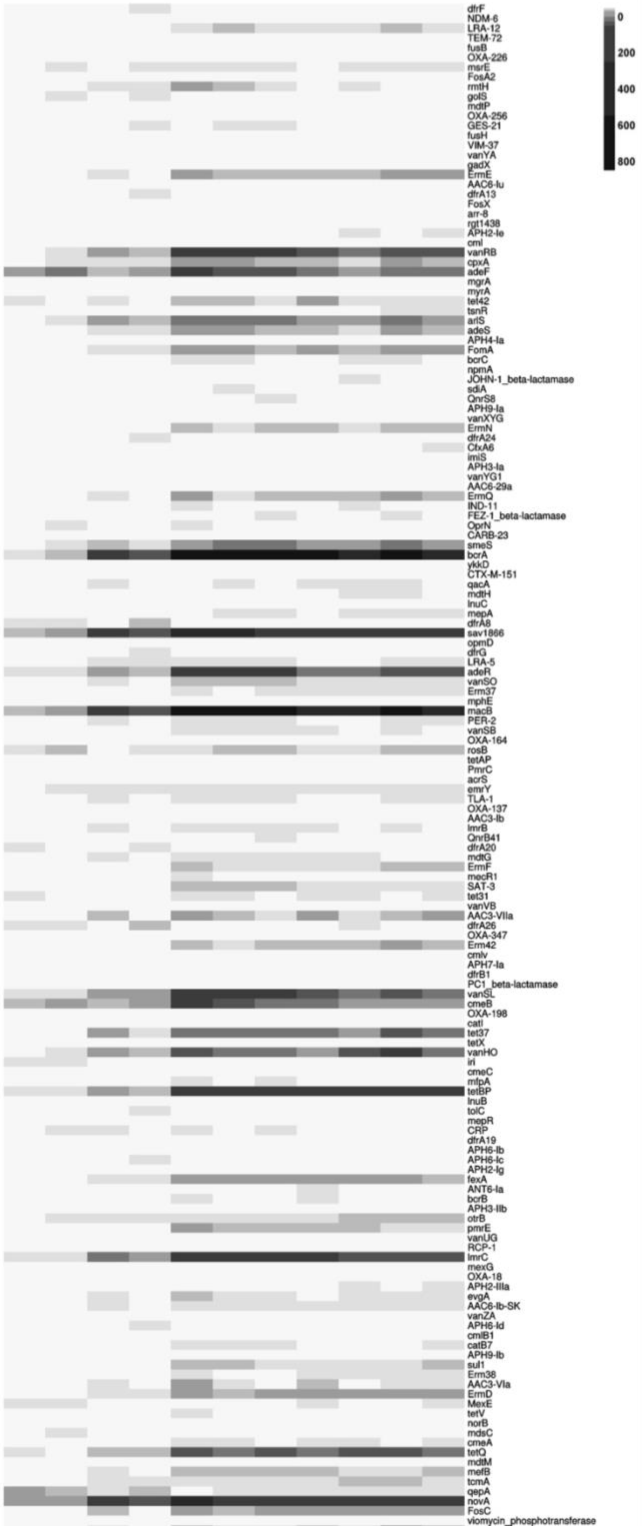

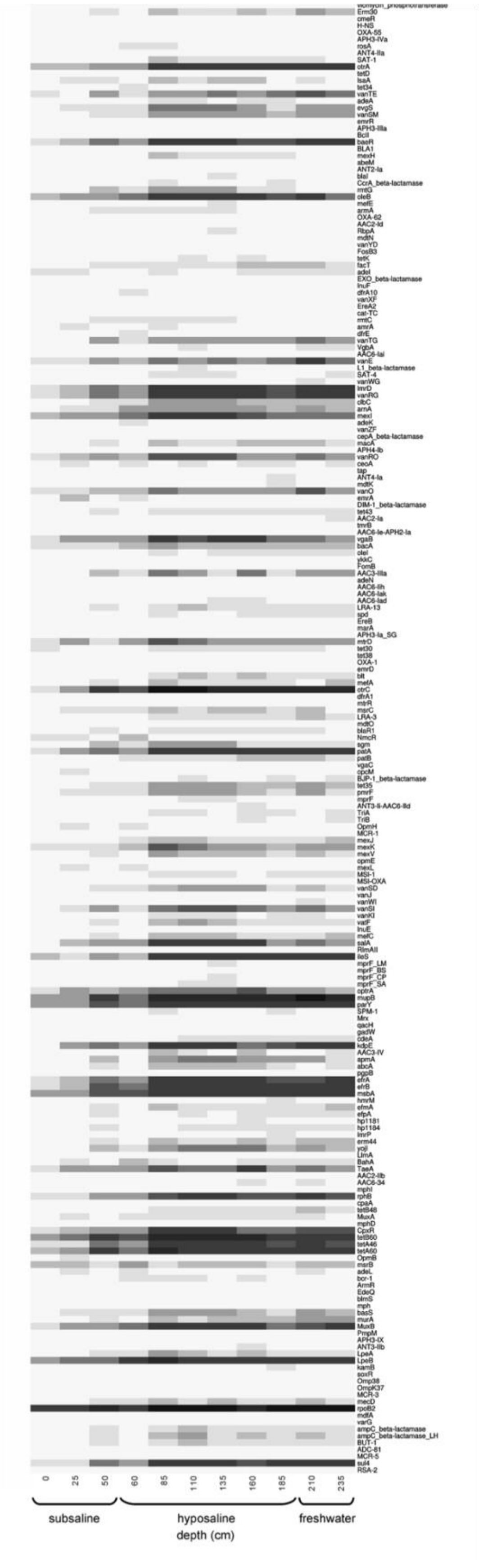
0
25
50
60
85
110
135
160
185
210
235
subsaline
hyposaline
freshwater
depth (cm)

**Figure S10.** (Top) Bar graph of the ten most abundant families of Eukarya, (bottom left) Histogram of the abundances of Fungi orders across three zones, (bottom right) Bar Graph of the Fungi genera in the 12 samples in the Holocene sediment sequence from Lake Chalco, Mexico.

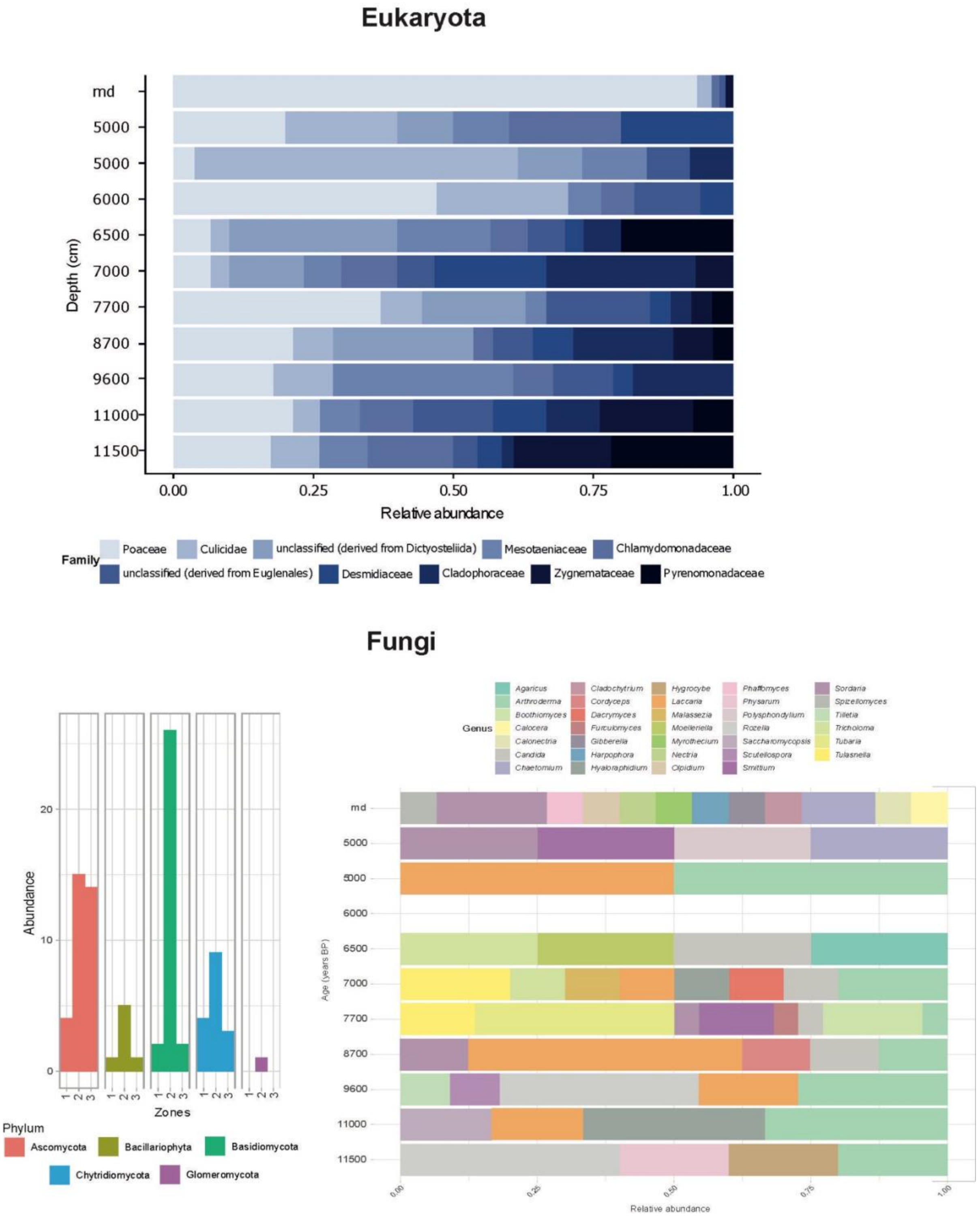

Table S1[1]. Metadata obtained from the twelve samples from a sedimentary sequence of the Lake Chalco

| MG-RAST ID | Sample name | Depth cm | Age | Temp | Salinity | Geochemical variable | | | | | | | | | | | |
|---|---|---|---|---|---|---|---|---|---|---|---|---|---|---|---|---|---|
| | | | | | | Mn/Fe | Ti/Fe | TOC | Si/Ti | Ca/Ti | P/Ti | C/N | Si | IC | P | Ca | Ti |
| mgm4736471.3 | S1 | 0 | Md | | | 0.012 | 0.187 | 1.844 | 85.70 | 25.23 | 0.69 | 5.15 | 24.09 | 6.52 | 0.20 | 7.09 | 0.28 |
| mgm4736462.3 | S2 | 0 | Md | Temperate | Subsaline | 0.012 | 0.187 | 1.844 | 85.70 | 25.23 | 4.25 | 5.15 | 24.09 | 6.52 | 1.20 | 7.09 | 0.28 |
| mgm4736465.3 | 50-1 | 25 | <6000 | | | 0.011 | 0.191 | 0.789 | 129.70 | 29.13 | 0.86 | 6.32 | 29.70 | 8.04 | 0.20 | 6.67 | 0.23 |
| mgm4736468.3 | 50-2 | 50 | <6000 | | | 0.003 | 0.218 | 1.026 | 158.47 | 8.15 | 0.82 | 7.82 | 30.89 | 8.63 | 0.16 | 1.59 | 0.19 |
| mgm4736463.3 | 100-1 | 60 | 6000 | | | 0.012 | 0.226 | 0.646 | 87.48 | 6.30 | 0.50 | 2.61 | 27.29 | 5.00 | 0.16 | 1.97 | 0.31 |
| mgm4736464.3 | 100-2 | 85 | 6500 | | | 0.000 | 0.219 | 6.087 | 528.05 | 318.74 | 1.67 | 10.73 | 22.80 | 7.54 | 0.07 | 13.76 | 0.04 |
| mgm4736461.3 | 100-3 | 110 | 7000 | Warm | Hyposaline | 0.006 | 0.147 | 1.898 | 86.71 | 76.84 | 0.43 | 7.52 | 17.80 | 9.20 | 0.09 | 15.77 | 0.21 |
| mgm4736467.3 | 100-4 | 135 | 7700 | | | 0.000 | 0.215 | 6.813 | 146.09 | 270.21 | 0.60 | 18.47 | 14.52 | 10.95 | 0.06 | 26.85 | 0.10 |
| mgm4736469.3 | 200-1 | 160 | 8700 | | | 0.013 | 0.169 | 3.197 | 99.72 | 82.06 | 0.57 | 8.02 | 18.98 | 7.90 | 0.11 | 15.62 | 0.19 |
| mgm4736460.3 | 200-2 | 185 | 9600 | | | 0.004 | 0.164 | 1.360 | 148.14 | 68.76 | 1.12 | 11.40 | 24.12 | 12.97 | 0.18 | 11.20 | 0.16 |
| mgm4736466.3 | 200-3 | 210 | 11000 | Cold | Freshwater | 0.014 | 0.170 | 1.683 | 639.72 | 29.33 | 2.36 | 1.14 | 87.38 | 3.36 | 0.32 | 4.01 | 0.14 |
| mgm4736470.3 | 200-4 | 235 | 11500 | | | 0.010 | 0.144 | 0.249 | 678.79 | 23.36 | 2.20 | 0.96 | 85.39 | 3.44 | 0.28 | 2.94 | 0.13 |

[1] Metadata obtained from the twelve samples taken from a sediment sequence collected in Lake Chalco. MGRAST ID: ID of the MG-RAST server; Samples name; Depth (cm); Age (cal yr BP); Temp (diatom-inferred temperature; Salinity (diatom-inferred salinity) Geochemical variables (elements, element ratios, total organic carbon [TOC], and inorganic carbon [IC]). The units for elements, IC, TOC, and C/N are %.

Table S2: Taxonomy enriched genera in the Log2 fold change analysis between three established zones[1]*

| Zone | Phylum | Family | Genus | Description | Ref. |
|---|---|---|---|---|---|
| Freshwater **Zone 1** | Crenarchaeota | Pyrodictiaceae | *Pyrodictium* | Hydrogen-sulphur-autotrophs able to reduce molecular sulphur. Thermophiles. | 1 |
| | | Fervidicoccaceae | *Fervidicoccus* | Obligate anaerobes and thermophiles. Two [NiFe]-hydrogenases are encoded: a membrane-bound energy-converting hydrogenase and a cytoplasmic one. | 2 |
| | Euryarchaeota | Thermococcaceae | *Thermococcus* | Anaerobic heterotrophs that ferment peptides and sugars, their growth is stimulated through sulfur reduction. Hyperthermophiles. | 3 |
| | | Archaeoglobaceae | *Archaeoglobus* | Gram negative. Blue-green fluorescence at 420 nm. Thermophiles and strictly anaerobic. Sulfate or sulfite and thiosulfate used as electron acceptors. Chemolithoautotrophic growth in the presence of $H_2/CO_2$. | 4 |
| | | Methanobacteriaceae | *Methanoregula* | Strictly anaerobic $H_2/CO_2$-utilizing methanogens. Optimal growth conditions near pH 5.1 and 35 °C. | 5 |
| | Firmicutes | Thermoanaerobacteraceae | *Ammonifex* | Gram-negative, extremely thermophilic, strictly anaerobic and optimal 0–1.5% NaCl. Muramic acid present. Core lipids consist mainly of (non-phytanyl) ether lipids and fatty acid. | 6,7 |
| | | Peptococcaceae | *Anaerofustis* | Stain Gram-positive, strictly anaerobic and catalase-negative. Resistant to 20% bile. Some carbohydrates are fermented. | 8 |
| Hyposaline **Zone 2** | Chloroflexi | Dehalococcoides | *Dehalococcoides* | Cells stain Gram-indifferent and peptidoglycan in the cell wall are absent-motiles. Obtain energy via the oxidation of hydrogen and subsequent reductive dehalogenation of halogenated organic compounds in a mode of anaerobic respiration. | 9 |
| | Firmicutes | Thermoanaerobacteraceae | *Ammonifex* | Gram-negative, extremely thermophilic, strictly anaerobic. Core lipids consist mainly of (non-phytanyl) ether lipids and fatty acid methyl esters. | 6,7 |
| | Crenarchaeota | Fervidicoccaceae | *Fervidicoccus* | Obligately anaerobic, extreme thermophiles. Two [NiFe]-hydrogenases are encoded: a membrane-bound energy-converting hydrogenase and a cytoplasmic one. | 2 |
| | | Pyrodictiaceae | *Pyrodictium* | Thermophiles formed highly unusual networks of fibres. Hydrogen-sulphur-autotrophs, able to reduce molecular sulphur. | 1 |

[1] Zone 1: freshwater; Zone 2: hyposaline and Zone 3: subsaline

| | | | | | |
|---|---|---|---|---|---|
| | Euryarchaeota | Archaeoglobus | *Archaeoglobus* | Gram negative. Blue-green fluorescence at 420 nm. Strictly anaerobic. Sulfate or sulfite and thiosulfate used as electron acceptors. Chemolithoautotrophic growth in the presence of $H_2/CO_2$. | 4 |
| | | Thermococcaceae | *Thermococcus* | Strictly anaerobic. Extremely thermophilic. NaCl is required for growth, with optimal concentrations between 2 and 4%. Elemental sulfur significantly stimulates growth rates with production of $H_2S$. | 3 |
| | | Methanobacteriaceae | *Methanoregula* | Strictly anaerobic $H_2/CO_2$-utilizing methanogens. Optimal growth conditions near pH 5.1 and 35 °C. | 5 |
| | | Methanomicrobiaceae | *Methanoculleus* | Stain Gram-negative. Cell wall is protein (S-layer of glycoprotein subunits), sensitive to lysis by detergents. Thermophilic or mesophilic. Rapid growth at NaCl concentration of 0.1–0.2 M. Chemolithotrophic, strictly anaerobic, utilizing $H_2/CO_2$. | 10 |
| | | Thermococcaceae | *Pyrococcus* | Gram-negative, strictly anaerobic and heterotrophic growth on peptone, tryptone, yeast extract, meat extract, extracts of Bacteria and Archaea, casein, starch, maltose, and Casamino acids. | 11 |
| Subsaline **Zone 3** | Gemmatimonadetes | Gemmatimonadaceae | *Gemmatimonas* | Gram-negative, aerobic and mesophilic. Cells grow under aerobic conditions. The major respiratory quinone is MK-9. Polyphosphate-accumulating, isolated from wastewater. | 12 |
| | Planctomycetes | Planctomycetaceae | *Pirellula* | Gram-stain-negative. Chemoorganotrophic; obligately aerobic. The major respiratory lipoquinone present is MK-6. | 13 |
| | Proteobacteria | (Alphaproteobacteria) Rhodospirillaceae | *Azospirillum* | Gram-negative, microaerophilic, non-fermentative and nitrogen-fixing. Plant growth promoting rhizobacteria (PGPR). They display a versatile C- and N-metabolism, which makes them well adapted to establishing the competitive environment of the rhizosphere. | 14 |
| | | (Betaproteobacteria) Alcaligenaceae | *Bordetella* | Gram negative and strictly aerobic. Optimal temperature, 35–37°C. Respiratory metabolism chemoorganotrophic. Require nicotinamide, organic sulfur (e.g., cysteine), and organic nitrogen (amino acids). Utilize oxidatively glutamic acid, proline, alanine, aspartic acid, and serine, with production of ammonia and $CO_2$. Mammalian and avian parasite and pathogen. | 15 |
| | | (Alphaproteobacteria) Brucellaceae | *Brucella* | Gram negative and aerobic, possessing a respiratory type of metabolism and having a cytochrome-based electron transport system with oxygen or nitrate as the terminal electron acceptor. Chemoorganotrophic. Many species are facultatively intracellular pathogens of animals and/or humans with species and some biovars showing distinct host tropisms. | 16 |

| | | | | | |
|---|---|---|---|---|---|
| | | (Alphaproteobacterial) Phyllobacteriaceae | *Chelativorans* | The strains were Gram-negative, strictly, aerobic, asporogenous and non-motile rods that required biotin for growth. The strains were mesophilic and neutrophilic. | 17 |
| | | (Betaproteobacteria) Oxalobacteraceae | *Collimonas* | Cells are strictly aerobic, straight or slightly curved, Gram-negative rods. Maximal growth is observed between 20 and 30°C. Chitinolytic bacterial that were able to grow at the expense of intact, living hyphae of several soil fungi. The genus *Collimonas* is only known to occur in slightly acidic sandy dune soils. | 18 |
| | | (Gammaproteobacteria) Coxiellaceae | *Coxiella* | Gram negative strain. Is an obligate intracellular acidophile that is highly adapted for life within the eukaryotic phagolysosomethe. *Coxiella burnetii*, the etiological agent of "Q fever," is a category B bioterrorism agent that is highly infective to both humans and livestock. | 19 |
| | | (Betaproteobacteria) Neisseriaceae | *Chromobacterium* | Gram negative, usually oxidase positive. No resting stages known. Growth in nutrient broth produces a violet ring at the surface with a fragile pellicle. Chemoorganotrophs; most strains (80%) attack carbohydrates fermentatively, some (20%) oxidatively, producing small amounts of acid but usually no gas. Lactate is oxidized to $CO_2$. Soil and water organisms, occasionally causing infections of humans and other mammals. | 20 |
| | | (Betaproteobacteria) Azonexaceae | *Dechloromonas* | Gram-negative, facultatively anaerobic, and strictly respiratory metabolism. Chlorate and perchlorate reduced to chloride, organic electron donors. $O_2$ also serves as an electron acceptor; some strains use nitrate as an electron acceptor. | 21 |
| | | (Gammaproteobacteria) Halomonadaceae | *Halomonas* | Gram negative, possess a mainly respiratory type of metabolism with oxygen as the terminal electron acceptor. Some species are also capable of anaerobic growth in the presence of nitrate. Chemoorganotrophic. Carbohydrates, amino acids, polyols, and hydrocarbons can serve as sole carbon sources in mineral media. Ammonium sulfate can serve as a sole nitrogen source. | 22 |
| | | (Gammaproteobacteria) Ectothiorhodospiraceae | *Halorhodospira* | Gram negative, is among the most halophilic organisms known. It is an obligately photosynthetic and anaerobic purple sulfur bacterium that exhibits autotrophic growth up to saturated NaCl concentrations. Have the ability of photoautotrophic growth under extreme conditions, and its characteristic sulfur metabolism. | 23 |
| | | (Alphaproteobacteria) Rhodobacteraceae | *Labrenzia* | Gram-negative, aerobic, the major respiratory lipoquinone, nitrate reduction to nitrite or nitrogen, and chemoheterotrophic and non-fermentative growth under aerobic and anaerobic conditions. | 24 |

| | | | | | |
|---|---|---|---|---|---|
| | | (Betaproteobacteria) Comamonadaceae | *Methylibium* | Gram-negative, aerobic, possible intracellular poly-β-hydroxyalkanoate granules (white spots) and possible protein inclusion bodies (dark spots) can also be observed, mesophilic, optimum temperatures 28°C and carbon source is from MTBE; TAME; TBA; methanol; ethanol. The genus might have an important role in MTBE biodegradation | 25 |
| | | (Betaproteobacteria) Neisseriaceae | *Laribacter* | The cells were facultatively anaerobic, gram-negative, mesophile. The bacterium can grow in 1 or 2% NaCl. No enhancement of growth is observed with 5% $CO_2$. A bacterium was isolated from the blood and empyema of a cirrhotic patient. | 26 |
| | | (Betaproteobacteria) Burkholderiales | *Leptothrix* | Gram negative and aerobic oxidative and chemoorganoheterotrophic. Sheaths can become encrusted by the deposition of iron and manganese oxides. They occur in standing or slow-flowing, ferruginous, neutral to slightly acidic fresh waters with only low concentrations of organic matter. pathogen species. | 27 |
| | | (Betaproteobacteria) Nitrosomonadaceae | *Methylovorus* | Gram negative. No capsules formed, but slime may be produced by some strains. Restricted facultatively methylotrophic. Assimilate methanol via the ribulose monophosphate pathway. | 28 |
| | | (Alphaproteobacteria) Methylobacteriaceae | *Methylobacterium* | Gram-negative bacilli that have been reported to be opportunistic pathogens in immunocompromised patients. Its normal habitats are soil and water. Are strictly aerobic, facultatively methylotrophic, fastidious, slow growing. | 29 |
| | | (Betaproteobacteria) Nitrosomonadaceae | *Nitrosomonas* | Gram-negative cell wall. Depending on the growth conditions, cells are free-living or embedded in slimy aggregates. Urea can be used as a nitrogen source by many, but not all, species. | 30 |
| | | (Betaproteobacteria) Nitrosomonadaceae | *Nitrosospira* | Gram-negative, aerobic, mesophilic, free-living, Autotroph and lithotroph. It is an ammonia-oxidizing bacterium. | 31 |
| | | *(Betaproteobacteria) Oxalobacteraceae* | Oxalobacter | Gram-negative. Strictly anaerobic. Chemoorganotroph. Oxalate is used as the major carbon and energy source. Strains have been isolated from the rumens of cattle and sheep, from cecal and fecal samples from humans, guinea pigs, swine, domestic and wild rats, and from freshwater lake and marine sediments. | 32 |
| | | (Alphaproteobacteria) Phyllobacteriaceae | *Parvibaculum* | Gram-negative, aerobic, chemoorgano-heterotroph bacteria that can be motile by means of a polar flagellum. | 33 |
| | | (Alphaproteobacteria) Caulobacteraceae | *Phenylobacterium* | Capsule stains are negative, strictly aerobic, and weakly oxidase positive. Chemoorganotrophic, having a strictly respiratory type of metabolism. | 34 |

| | | | | | |
|---|---|---|---|---|---|
| | | | | Optimal temperature, 28–30°C. Can grow on chloridazon. | |
| | | (Betaproteobacteria) Ralstoniaceae | *Ralstonia* | Gram-negative asporogenous rods, aerobic, having a strictly respiratory type of metabolism with oxygen as the terminal electron acceptor. Oxidase and catalase positive. Cellular lipids of this genus contain two kinds of phosphatidylethanolamine, PE-1 and PE-2. The genus contains plant pathogens, human pathogens, knallgas bacteria, and metal-resistant bacteria. | 35 |
| | | (Alphaproteobacteria) Bradyrhizobiaceae | *Rhodopseudomonas* | Purple non-sulfur phototrophic organisms that can be found in many types of marine environments and soils. It lives by converting sunlight to cellular energy and by absorbing atmospheric carbon dioxide and converting it to biomass | 36 |
| | | (Alphaproteobacteria) Rhodospirillaceae | *Rhodospirillum* | Gram-negative, purple non-sulfur bacteria that can grow aerobically or anaerobically. Contain intracytoplasmic photosynthetic membranes as vesicles, lamellae, or stacks, but not as finger-like intrusions of the cytoplasmic membrane. | 37 |
| | | (Gammaproteobacteria) Ectothiorhodospiraceae | *Thioalkalivibrio* | Gram-negative, and carbon source $HCO_3^-$. Is an obligately chemolithoautotrophic, haloalkaliphilic sulfur-oxidizing bacterium (SOB), using $CO_2$ as a carbon source and reduced inorganic sulfur compounds as an energy source. This genus is characterized as being obligate haloalkaliphilic. | 38 |
| | | (Alphaproteobacterial) Xanthobacteraceae | *Xanthobacter* | A genus of gram-negative, aerobic, rod-shaped bacteria found in wet soil containing decaying organic material and in water. | 39 |
| | | (Gammaproteobacteria) Xanthomonadaceae | *Xanthomonas* | Gram negative, aerobic, oxidative, and catalase positive, relatively non- fastidious in nutritional requirements but usually not truly prototrophic; capable of utilizing a relatively narrow selection of carbon sources. Can cause diseases to plants. | 40 |
| | | (Betaproteobacteria) Burkholderiaceae | *Burkholderia* | Gram-negative, obligately aerobic. Are able to utilize poly-beta-hydroxybutyrate (PHB) for growth. The genus includes both animal and plant pathogens, as well as some environmentally important species. | 41 |
| | | (Alphaproteobacteria) Acetobacteraceae | *Granulibacter* | Gram-negative, aerobic. Up until now there is only one species of this genus known (*Granulibacter bethesdensis*). These bacteria were identified in a series of patients with chronic granulomatous disease (CGD). | 42 |
| | Actinobacteria | Pseudonocardiaceae | *Saccharomonospora* | Gram-positive stain, aerobic, and chemo-organotrophic. Mesophilic or thermophilic; growth occurs between 24 and 60°C, and at neutral pH. NaCl may be required for growth. | 43 |

References

1. Stetter, K. O., König, H. & Stackebrandt, E. Pyrodictium gen. nov., a New Genus of Submarine Disc-Shaped Sulphur Reducing Archaebacteria Growing Optimally at 105°C. *Syst. Appl. Microbiol.* (1983) doi:10.1016/S0723-2020(83)80011-3.
2. Lebedinsky, A. V. *et al.* Analysis of the complete genome of Fervidococcus fontis confirms the distinct phylogenetic position of the order Fervidicoccales and suggests its environmental function. *Extremophiles* (2014) doi:10.1007/s00792-013-0616-7.
3. Holden, J. F. *et al.* Diversity among three novel groups of hyperthermophilic deep-sea Thermococcus species from three sites in the northeastern Pacific Ocean. *FEMS Microbiol. Ecol.* (2001) doi:10.1016/S0168-6496(01)00118-0.
4. Huber, H. & Stetter, K. O. Archaeoglobales ord. nov. *Bergey's Manual of Systematics of Archaea and Bacteria* 1–1 (2015) doi:10.1002/9781118960608.obm00047.
5. Zinder, S. & Bräuer, S. Methanoregula. *Bergey's Manual of Systematics of Archaea and Bacteria* 1–8 (2016) doi:10.1002/9781118960608.gbm01368.
6. Miroshnichenko, M. L. *et al.* Caldicellulosiruptor kronotskyensis sp. nov. and Caldicellulosiruptor hydrothermalis sp. nov., two extremely thermophilic, cellulolytic, anaerobic bacteria from Kamchatka thermal springs. *Int. J. Syst. Evol. Microbiol.* (2008) doi:10.1099/ijs.0.65236-0.
7. Huber, R. Ammonifex. *Bergey's Manual of Systematics of Archaea and Bacteria* 1–4 (2015) doi:10.1002/9781118960608.gbm00744.
8. Lawson, P. A. Anaerofustis. Bergey's Manual of Systematics of Archaea and Bacteria. (2015).
9. Löffler, F. E. *et al.* Dehalococcoides mccartyi gen. nov., sp. nov., obligately organohalide-respiring anaerobic bacteria relevant to halogen cycling and bioremediation, belong to a novel bacterial class, Dehalococcoidia classis nov., order Dehalococcoidales ord. nov. and family Dehalococcoidaceae fam. nov., within the phylum Chloroflexi. *Int. J. Syst. Evol. Microbiol.* **63**, 625–635 (2013).
10. Chong, S. C. & Boone, D. R. Methanoculleus. *Bergey's Manual of Systematics of Archaea and Bacteria* 1–5 (2015) doi:10.1002/9781118960608.gbm00505.
11. Stetter, K. O. & Huber, H. Pyrococcus. *Bergey's Manual of Systematics of Archaea and Bacteria* 1–5 (2015) doi:10.1002/9781118960608.gbm00521.
12. Zhang, H. *et al.* Gemmatimonas aurantiaca gen. nov., sp. nov., a Gram-negative, aerobic, polyphosphate-accumulating micro-organism, the first cultured

representative of the new bacterial phylum Gemmatimonadetes phyl. nov. *Int. J. Syst. Evol. Microbiol.* (2003) doi:10.1099/ijs.0.02520-0.

13. Fuerst, J. A. & Butler, M. K. Pirellula. in *Bergey's Manual of Systematics of Archaea and Bacteria* (2015). doi:10.1002/9781118960608.gbm00785.
14. Steenhoudt, O. & Vanderleyden, J. Azospirillum, a free-living nitrogen-fixing bacterium closely associated with grasses: Genetic, biochemical and ecological aspects. *FEMS Microbiology Reviews* (2000) doi:10.1016/S0168-6445(00)00036-X.
15. Sanden, G. N. & Weyant, R. S. Bordetella Moreno-López 1952, 178 AL. in *Bergey's Manual® of Systematic Bacteriology* (2006). doi:10.1007/0-387-29298-5_154.
16. Liu, D. Brucella. in *Molecular Medical Microbiology* 1781–1788 (Elsevier, 2015).
17. Doronina, N. V. *et al.* Chelativorans multitrophicus gen. nov., sp. nov. and Chelativorans oligotrophicus sp. nov., aerobic EDTA-degrading bacteria. *Int. J. Syst. Evol. Microbiol.* (2010) doi:10.1099/ijs.0.003152-0.
18. de Boer, W. *et al.* Collimonas fungivorans gen. nov., sp. nov., a chitinolytic soil bacterium with the ability to grow on living fungal hyphae. *Int. J. Syst. Evol. Microbiol.* (2004) doi:10.1099/ijs.0.02920-0.
19. Seshadri, R. *et al.* Complete genome sequence of the Q-fever pathogen Coxiella burnetii. *Proc. Natl. Acad. Sci. U. S. A.* (2003) doi:10.1073/pnas.0931379100.
20. Gillis, M. & Logan, N. A. Chromobacterium. in *Bergey's Manual of Systematics of Archaea and Bacteria* (2015). doi:10.1002/9781118960608.gbm00975.
21. Dechloromonas. in *Bergey's Manual of Systematics of Archaea and Bacteria* (2015). doi:10.1002/9781118960608.gbm00998.
22. Vreeland, R. H. Halomonas. in *Bergey's Manual of Systematics of Archaea and Bacteria* (2015). doi:10.1002/9781118960608.gbm01190.
23. Challacombe, J. F. *et al.* Complete genome sequence of Halorhodospira halophila SL1. *Stand. Genomic Sci.* (2013) doi:10.4056/sigs.3677284.
24. Wang, Z. *et al.* Complete genome sequence of Labrenzia sp. strain CP4, isolated from a selfregenerating biocathode biofilm. *Genome Announc.* (2016) doi:10.1128/genomeA.00354-16.
25. Szabó, Z. *et al.* Draft genome sequence of Methylibium sp. strain T29, a novel fuel oxygenate-degrading bacterial isolate from Hungary. *Stand. Genomic Sci.* (2015) doi:10.1186/s40793-015-0023-z.

26. Yuen, K. Y. *et al.* Laribacter hongkongensis gen. nov., sp. nov., a novel gram-negative bacterium isolated from a cirrhotic patient with bacteremia and empyema. *J. Clin. Microbiol.* (2001) doi:10.1128/JCM.39.12.4227-4232.2001.

27. Spring, S. & Kämpfer, P. Leptothrix Kützing 1843, 184 AL. in *Bergey's Manual® of Systematic Bacteriology* (2006). doi:10.1007/0-387-29298-5_173.

28. Methylovorus. in *Bergey's Manual of Systematics of Archaea and Bacteria* (2015). doi:10.1002/9781118960608.gbm00972.

29. Kovaleva, J., Degener, J. E. & Van Der Mei, H. C. Methylobacterium and its role in health care-associated infection. *Journal of Clinical Microbiology* (2014) doi:10.1128/JCM.03561-13.

30. Koops, H.-P. & Pommerening-Röser, A. Nitrosomonas. in *Bergey's Manual of Systematics of Archaea and Bacteria* (2015). doi:10.1002/9781118960608.gbm00991.

31. Jiang, Q. Q. & Bakken, L. R. Comparison of Nitrosospira strains isolated from terrestrial environments. *FEMS Microbiol. Ecol.* **30**, 171–186 (1999).

32. Allison, M. J., MacGregor, B. J., Sharp, R. & Stahl, D. A. Oxalobacter. in *Bergey's Manual of Systematics of Archaea and Bacteria* (2015). doi:10.1002/9781118960608.gbm00966.

33. Schleheck, D., Dong, W., Denger, K., Heinzle, E. & Cook, A. M. An α-Proteobacterium Converts Linear Alkylbenzenesulfonate Surfactants into Sulfophenylcarboxylates and Linear Alkyldiphenyletherdisulfonate Surfactants into Sulfodiphenylethercarboxylates. *Applied and Environmental Microbiology* vol. 66 1911–1916 (2000).

34. Eberspächer, J. Phenylobacterium. in *Bergey's Manual of Systematics of Archaea and Bacteria* (2015). doi:10.1002/9781118960608.gbm00793.

35. Yabuuchi, E., Kawamura, Y. & Ezaki, T. Ralstonia. *Bergey's Manual of Systematics of Archaea and Bacteria* 1–21 (2015) doi:10.1002/9781118960608.gbm00941.

36. Larimer, F. W. *et al.* Complete genome sequence of the metabolically versatile photosynthetic bacterium Rhodopseudomonas palustris. *Nat. Biotechnol.* (2004) doi:10.1038/nbt923.

37. Imhoff, J. F., Truper, H. G. & Pfennig, N. Rearrangement of the species and genera of the phototrophic 'purple nonsulfur bacteria'. *Int. J. Syst. Bacteriol.*

(1984) doi:10.1099/00207713-34-3-340.

38. Muyzer, G. *et al.* Complete genome sequence of 'Thioalkalivibrio sulfidophilus' HL-EbGr7. *Stand. Genomic Sci.* (2011) doi:10.4056/sigs.1483693.

39. Garrity, G. M., Bell, J. A. & Lilburn, T. Acidithiobacillales ord. nov. in *Bergey's Manual® of Systematic Bacteriology* (2005). doi:10.1007/0-387-28022-7_2.

40. Starr, M. P. The Genus Xanthomonas. in *The Prokaryotes* (1981). doi:10.1007/978-3-662-13187-9_62.

41. Hotta, H. *et al.* Proposal of Burkholderia gen. Anov. And Transfer of Seven Species of the Genus Pseudomonas Homology Group II to the New Genus, with the Type Species Burkholderia cepacia (Palleroni and Holmes 1981) comb: Nov. *Microbiol. Immunol.* (1992) doi:10.1111/j.1348-0421.1992.tb02129.x.

42. Greenberg, D. E. *et al.* Granulibacter bethesdensis gen. nov., sp. nov., a distinctive pathogenic acetic acid bacterium in the family Acetobacteraceae. *Int. J. Syst. Evol. Microbiol.* (2006) doi:10.1099/ijs.0.64412-0.

43. Kim, S. B. Saccharomonospora. *Bergey's Manual of Systematics of Archaea and Bacteria* 1–11 (2015) doi:10.1002/9781118960608.gbm00185.

Table S3: Cyanobacteria species and proteins related across the sediment sequence by MetaProt analysis.

| Gene | Description | Species | Zone3 (9.39%) | Zone 2 (60%) | Zone 1 (30%) | Abundance |
|---|---|---|---|---|---|---|
| fig\|292563.3.peg.669 | Alpha-glucoside transport system permease protein AglG | *Cyanobacterium stanieri* PCC 7202 | **2** | **59** | **115** | **176** |
| fig\|292563.3.peg.670 | ABC alpha-glucoside transporter inner membrane subunit AglF | *Cyanobacterium stanieri* PCC 7202 | **24** | **59** | **35** | **118** |
| fig\|755178.3.peg.298 | Alkaline phosphatase EC 3.1.3.1 | *Cyanobacterium aponinum* PCC 10605 | **0** | **62** | **11** | **73** |
| fig\|292563.3.peg.672 | Alpha-glucosides-binding periplasmic protein AglE precursor | *Cyanobacterium stanieri* PCC 7202 | **0** | **40** | **14** | **54** |
| fig\|292563.3.peg.704 | Threonyl-tRNA synthetase EC 6.1.1.3 | *Cyanobacterium stanieri* PCC 7202 | **0** | **31** | **16** | **47** |
| fig\|755178.3.peg.840 | 3-oxoacyl-acyl-carrier-protein synthase KASII EC 2.3.1.41 | Cyanobacterium aponinum PCC 10605 | **0** | **21** | **4** | **25** |
| fig\|713887.3.peg.759 | 3-oxoacyl-acyl-carrier-protein reductase EC 1.1.1.100 | cyanobacterium UCYN-A | **0** | **23** | **0** | **23** |
| fig\|292563.3.peg.513 | Transporter | Cyanobacterium stanieri PCC 7202 | **0** | **15** | **6** | **21** |
| fig\|755178.3.peg.3556 | Porphobilinogen synthase EC 4.2.1.24 | Cyanobacterium aponinum PCC 10605 | **0** | **18** | **0** | **18** |
| fig\|180281.4.peg.2139 | Transaldolase EC 2.2.1.2 | Cyanobium sp. PCC 7001 | **5** | **12** | **0** | **17** |
| fig\|755178.3.peg.2451 | Aldehyde dehydrogenase EC 1.2.1.3 | Cyanobacterium aponinum PCC 10605 | **0** | **16** | **0** | **16** |
| fig\|292563.3.peg.1940 | Ketol-acid reductoisomerase EC 1.1.1.86 | Cyanobacterium stanieri PCC 7202 | **0** | **13** | **0** | **13** |
| fig\|292564.3.peg.1782 | L-diaminopimelate aminotransferase EC 2.6.1.83 | Cyanobium gracile PCC 6307 | **1** | **12** | **0** | **13** |
| fig\|321327.20.peg.2777 | Gamma-glutamyl phosphate reductase EC 1.2.1.41 | cyanobacterial subgroup Synechococcus sp. JA-3-3Ab | **0** | **13** | **0** | **13** |
| fig\|292563.3.peg.385 | 3-oxoacyl-acyl-carrier-protein synthase KASII EC 2.3.1.41 | Cyanobacterium stanieri PCC 7202 | **0** | **7** | **5** | **12** |
| fig\|755178.3.peg.2674 | Arsenical-resistance protein ACR3 | Cyanobacterium aponinum PCC 10605 | **0** | **12** | **0** | **12** |
| fig\|292563.3.peg.2674 | Acetyl-CoA synthetase ADP-forming alpha and beta chains putative | Cyanobacterium stanieri PCC 7202 | **11** | **0** | **0** | **11** |
| fig\|292564.3.peg.2080 | Similar to CDP-glucose 4 6-dehydratase EC 4.2.1.45 | Cyanobium gracile PCC 6307 | **0** | **10** | **0** | **10** |
| fig\|292563.3.peg.1780 | GTP-binding and nucleic acid-binding protein YchF | Cyanobacterium stanieri PCC 7202 | **0** | **0** | **9** | **9** |
| fig\|755178.3.peg.1775 | ATP-dependent Clp protease ATP-binding subunit ClpC / Negative regulator of genetic competence clcC/mecB | Cyanobacterium aponinum PCC 10605 | **0** | **9** | **0** | **9** |
| fig\|292563.3.peg.1217 | GDP-D-mannose dehydratase | Cyanobacterium stanieri PCC 7202 | **0** | **8** | **0** | **8** |
| fig\|316067.3.peg.3679 | Phytochrome two-component sensor histidine kinase EC 2.7.3.- | Cyanobacterial phytochrome B Geobacter sp. FRC-32 | **0** | **0** | **8** | **8** |
| fig\|755178.3.peg.1387 | Enolase EC 4.2.1.11 | Cyanobacterium aponinum PCC 10605 | **0** | **0** | **7** | **7** |
| fig\|755178.3.peg.3519 | NiFe hydrogenase metallocenter assembly protein HypF | *Cyanobacterium aponinum* PCC 10605 | **0** | **0** | **7** | **7** |
| fig\|292563.3.peg.2169 | dTDP-glucose 4 6-dehydratase EC 4.2.1.46 | *Cyanobacterium stanieri* PCC 7202 | **0** | **6** | **0** | **6** |

| | | | | | | |
|---|---|---|---|---|---|---|
| fig\|342108.5.peg.3106 | Phytochrome two-component sensor histidine kinase EC 2.7.3.- Cyanobacterial phytochrome B | *Magnetospirillum magneticum* AMB-1 | **0** | **6** | **0** | **6** |
| fig\|755178.3.peg.1456 | Zinc ABC transporter ATP-binding protein ZnuC | *Cyanobacterium aponinum* PCC 10605 | **0** | **6** | **0** | **6** |
| fig\|755178.3.peg.3012 | melibiose carrier protein | *Cyanobacterium aponinum* PCC 10605 | **0** | **6** | **0** | **6** |
| fig\|180281.4.peg.66 | Arylsulfatase EC 3.1.6.1 | *Cyanobium sp*. PCC 7001 | **5** | **0** | **0** | **5** |
| fig\|755178.3.peg.2506 | hypothetical protein | *Cyanobacterium aponinum* PCC 10605 | **0** | **5** | **0** | **5** |
| fig\|755178.3.peg.924 | Circadian clock protein KaiC | *Cyanobacterium aponinum* PCC 10605 | **0** | **5** | **0** | **5** |
| fig\|292563.3.peg.989 | YoeB toxin protein | *Cyanobacterium stanieri* PCC 7202 | **0** | **4** | **0** | **4** |
| fig\|292564.3.peg.48 | Acetolactate synthase large subunit EC 2.2.1.6 | *Cyanobium gracile* PCC 6307 | **0** | **2** | **2** | **4** |
| fig\|1170562.3.peg.5094 | Cyanobacteria-specific RpoD-like sigma factor type-2 @ Group 2 RNA polymerase sigma factor | *Calothrix sp*. PCC 6303 | **0** | **0** | **3** | **3** |
| fig\|180281.4.peg.2004 | Aspartyl-tRNAAsn amidotransferase subunit A EC 6.3.5.6 @ Glutamyl-tRNAGln amidotransferase subunit A EC 6.3.5.7 | *Cyanobium sp.* PCC 7001 | **3** | **0** | **0** | **3** |
| fig\|292563.3.peg.2826 | Translation elongation factor LepA | *Cyanobacterium stanieri* PCC 7202 | **0** | **3** | **0** | **3** |
| fig\|292563.3.peg.319 | Acetyl-coenzyme A synthetase EC 6.2.1.1 | *Cyanobacterium stanieri* PCC 7202 | **3** | **0** | **0** | **3** |
| fig\|292564.3.peg.2452 | Catalase EC 1.11.1.6 / Peroxidase EC 1.11.1.7 | *Cyanobium gracile* PCC 6307 | **0** | **3** | **0** | **3** |
| fig\|292564.3.peg.45 | Phosphoenolpyruvate synthase EC 2.7.9.2 | *Cyanobium gracile* PCC 6307 | **1** | **2** | **0** | **3** |
| fig\|755178.3.peg.146 | Glutamate-1-semialdehyde aminotransferase EC 5.4.3.8 | *Cyanobacterium aponinum* PCC 10605 | **0** | **0** | **3** | **3** |
| fig\|755178.3.peg.2833 | hypothetical protein | *Cyanobacterium aponinum* PCC 10605 | **0** | **3** | **0** | **3** |
| fig\|755178.3.peg.581 | Urease alpha subunit EC 3.5.1.5 | *Cyanobacterium aponinum* PCC 10605 | **3** | **0** | **0** | **3** |
| fig\|180281.4.peg.1750 | Threonine dehydratase biosynthetic EC 4.3.1.19 | *Cyanobium sp*. PCC 7001 | **2** | **0** | **0** | **2** |
| fig\|247490.3.peg.762 | Phytochrome two-component sensor histidine kinase EC 2.7.3.-Cyanobacterial phytochrome B | *Planctomycete* KSU-1 | **0** | **0** | **2** | **2** |
| fig\|292563.3.peg.2716 | Phosphoadenylyl-sulfate reductase thioredoxin EC 1.8.4.8 | *Cyanobacterium stanieri* PCC 7202 | **0** | **0** | **2** | **2** |
| fig\|292563.3.peg.3007 | Xaa-Pro aminopeptidase EC 3.4.11.9 | *Cyanobacterium stanieri* PCC 7202 | **2** | **0** | **0** | **2** |
| fig\|292564.3.peg.1837 | Type I restriction-modification system DNA-methyltransferase subunit M EC 2.1.1.72 | *Cyanobium gracile* PCC 6307 | **2** | **0** | **0** | **2** |
| fig\|292564.3.peg.3238 | Pyruvate-flavodoxin oxidoreductase EC 1.2.7.- | *Cyanobium gracile* PCC 6307 | **0** | **2** | **0** | **2** |
| fig\|316067.3.peg.1880 | Phytochrome two-component sensor histidine kinase EC 2.7.3.-Cyanobacterial phytochrome B | *Geobacter sp*. FRC-32 | **0** | **0** | **2** | **2** |
| fig\|388739.3.peg.770 | Folate carrier cyanobacterial type | *Roseobacter sp*. SK209-2-6 | **2** | **0** | **0** | **2** |
| fig\|713887.3.peg.813 | Nodulation protein nolO EC 2.1.3.- | *Cyanobacterium* UCYN-A | **2** | **0** | **0** | **2** |
| fig\|755178.3.peg.140 | Asparaginyl-tRNA synthetase EC 6.1.1.22 | *Cyanobacterium aponinum* PCC 10605 | **2** | **0** | **0** | **2** |

| | | | | | | |
|---|---|---|---|---|---|---|
| fig\|755178.3.peg.3699 | adenine specific DNA methyltransferase | *Cyanobacterium aponinum* PCC 10605 | **0** | **2** | **0** | **2** |
| fig\|755178.3.peg.658 | Transporter | *Cyanobacterium aponinum* PCC 10605 | **0** | **0** | **2** | **2** |
| fig\|180281.4.peg.1829 | Leucyl-tRNA synthetase EC 6.1.1.4 | *Cyanobium sp*. PCC 7001 | **0** | **1** | **0** | **1** |
| fig\|292563.3.peg.3002 | Glutamate synthase NADPH small chain EC 1.4.1.13 | *Cyanobacterium stanier*i PCC 7202 | **1** | **0** | **0** | **1** |
| fig\|292563.3.peg.638 | Glutamate synthase NADPH large chain EC 1.4.1.13 | *Cyanobacterium stanieri* PCC 7202 | **1** | **0** | **0** | **1** |
| fig\|292564.3.peg.1220 | Arylsulfatase EC 3.1.6.1 | *Cyanobium gracile* PCC 6307 | **1** | **0** | **0** | **1** |
| fig\|292564.3.peg.1433 | Putative oxidoreductase | *Cyanobium gracile* PCC 6307 | **0** | **1** | **0** | **1** |
| fig\|292564.3.peg.1839 | Type I restriction-modification system restriction subunit R EC 3.1.21.3 | *Cyanobium gracile* PCC 6307 | **1** | **0** | **0** | **1** |
| fig\|292564.3.peg.223 | Type II restriction enzyme methylase subunits | *Cyanobium gracile* PCC 6307 | **0** | **1** | **0** | **1** |
| fig\|292564.3.peg.2238 | hypothetical protein | *Cyanobium gracile* PCC 6307 | **1** | **0** | **0** | **1** |
| fig\|378806.7.peg.198 | Phytochrome two-component sensor histidine kinase EC 2.7.3.- Cyanobacterial phytochrome B | *Stigmatella aurantiaca* DW4/3-1 | **0** | **1** | **0** | **1** |
| fig\|522306.3.peg.2806 | Folate carrier cyanobacterial type | *Accumulibacter phosphat*is clade IIA str. UW-1 | **1** | **0** | **0** | **1** |
| fig\|697282.3.peg.2964 | Phytochrome two-component sensor histidine kinase EC 2.7.3.- Cyanobacterial phytochrome B | *Methylobacter tundripaludum* SV96 | **0** | **1** | **0** | **1** |
| fig\|755178.3.peg.1250 | Aminotransferase DegT/DnrJ/EryC1/StrS family EC 4.2.1.- | *Cyanobacterium aponinum* PCC 10605 | **1** | **0** | **0** | **1** |
| fig\|755178.3.peg.81 | Carbamoyl-phosphate synthase large chain EC 6.3.5.5 | *Cyanobacterium aponinum* PCC 10605 | **1** | **0** | **0** | **1** |
| | | **Total** | **78** | **500** | **253** | **831** |

Table S4: Protein enrichment genes in the Log2 fold change analysis among three established zones

| Zone | L1 | L2 | L3 | Function | Description | Ref |
|---|---|---|---|---|---|---|
| **Freshwater** | Metabolism_of_Aromatic_ Compounds | NULL | Aromatic_Amin_Catabolism | 4-hydroxyphenylacetate_3-monooxygenase__EC_1.14.13.3_ | Sequence analysis suggests that several aromatic compound-hydroxylating enzymes are FADH2-utilizing monooxygenases, involved in the bio-degradation of xenobiotic compounds or biosynthesis of antibiotics and siderophores. | 1 |
| **Hyposaline** | RNA_Metabolism | RNA_processing_and_ modification | tRNA_modification_Archaea | archaeosine_tRNA-ribosyltransferase__EC_2.4.2.-__PUA_domain | Also known as tRNA-guanine transglycosylase (TGT). In the biosynthesis of archaeosine the TGT catalyzes the replacement of guanine at position 15 in the D loop of most tRNAs by a free precursor base. The recognition of TGT is sufficiently limited and specific to allow the enzyme to recognize efficiently any tRNA species whose structure is not fully stabilized in an extremely high-temperature environment | 2 |
| | Membrane_Transport | NULL | Transport_of_Nickel_and_Cobalt | Nickel_responsive_regulator_NikR | The NikR protein is able to function both as a repressor and as an activator of gene transcription, depending on the position of the binding site. NikR was first identified in *Escherichia coli*, where it functions as a transcriptional repressor of the Nik nickel uptake system. NikR mediates its repressor function via nickel-dependent binding to a palindromic sequence in the promoter region of the nik operon. The net result of this regulation is expression of the Nik system only when nickel is scarce in the cell. | 3 |

| | | | | | | |
|---|---|---|---|---|---|---|
| | Cofactors_Vitamins_Prosthetic_Groups_Pigments | Folate_and_pterines | Folate_Biosynthesis | Thymidylate_synthase_thyX__EC_2.1.1 | This is an important enzyme, which is responsible for the reductive methylation of deoxyuridylic acid (dUMP, 7) to deoxythymidylic acid (dTMP, 8). The methylation of the uracil moiety (present in RNA) to 5-methyl uracil (thymine, present in DNA) requires participation of a folic acid coenzyme, N5, N10-methylenetetrahydrofolate as a methyl donor. The functioning of thymidylate synthetase is coupled with the activity of DHF reductase. That is why this biochemical target is usually referred to as thymidylate synthetase/ DHF reductase. | 4 |
| | Clustering-based_subsystems | NULL | CBSS-290633.1.peg.1906 | GTP-binding_protein_EngA | The first member of a putative bacterial GTP-binding protein family was identified in *Neisseria gonorrhoeae*. This protein was named EngA (essential neisserial GTP-binding proteinA). Interestingly, this protein contains two sets of GTP-binding motifs as defined by Bourne et al. (1991) which is in contrast to the other GTP-binding proteins which possess one set. This family contains exclusively gram positive eubacterial orthologs. | 5 |
| **Subsaline** | DNA_Metabolism | DNA_repair | DNA_Repair_Base_Excision | ATP-dependent_DNA_ligase__EC_6.5.1.1__clustered_with_Ku_protein_LigD | The bacterial LigD proteins are putative multifunctional DNA-modifying enzymes composed of an ATP-dependent DNA ligase catalytic domain fused to a putative nuclease domain and a putative polymerase domain. Although genetic analysis showed that the LigD protein is required for efficient NHEJ in mycobacterium. Indeed, it has not yet been demonstrated that bacterial LigD proteins actually have any catalytic functions other than DNA ligation. | 6 |

| | | | | | | |
|---|---|---|---|---|---|---|
| | Clustering-based_subsystems | proteosome_related | Cluster-based_Subsystem_ Grouping_Hypotheticals_- _perhaps_Proteosome_Related | Bacterial_proteasome-activating_AAA-ATPase__PAN_ | AAA proteins are functionally and organizationally diverse, and vary in activity, stability, and mechanism. Members of the AAA family are found in all organisms and they are essential for many cellular functions. They are involved in processes such as DNA replication, protein degradation, membrane fusion, microtubule severing, peroxisome biogenesis, signal transduction and the regulation of gene expression. | 7 |
| | Metabolism_of_Aromatic_Compounds | NULL | Phenylacetyl-CoA_catabolic_pathway__core_ | Phenylacetic_acid_degradation_protein_PaaN_ring-opening_aldehyde_dehydrogenase__EC_1.2.1.3_ | This enzyme is proposed to act in the ring-opening step of phenylacetic acid degradation which follows ligation of the acid with coenzyme A (by PaaF) and hydroxylation by a multicomponent non-heme iron hydroxylase complex. This enzyme is related to aldehyde dehydrogenase family. This family includes sequences from *Burkholderia, Bordetella, Streptomyces*. | 8,9 |
| | Clustering-based_subsystems | Sulfatases_and_ sulfatase_modifying_ factor_1__and_a_ hypothetical_ | Sulfatases_and_sulfatase_ modifying_factor_1 | FIG068086:_hypothetical_protein | Is a member of a gene family that has been highly conserved during evolution, from bacteria to humans. Sulfatase Modifying Factor 1 encodes the formylglycine-generating enzyme, which is responsible for the cysteine to Cα-formylglycine conversion. | 10 |

| | | | | | | |
|---|---|---|---|---|---|---|
| | Clustering-based_subsystems | Sulfatases_and_ sulfatase_modifying_ factor_1__and_a_ hypothetical_ | Sulfatases_and_sulfatase_ modifying_factor_1 | Arylsulfatase__EC_3.1.6.1_ | Much of the sulfur in surface soils is in the form of sulfate esters (organic sulfates) and it suggested that sulfatases may play an important role in the processes whereby organic soil sulfur is mineralized and made available for plant growth. The aryl sulfatases hydrolyze sulfate esters with an aromatic radical (phenolic esters of sulfuric acid) and were originally described as phenol sulfatases. | 11 |
| | Carbohydrates | Central_carbohydrate_ metabolism | TCA_Cycle | Dihydrolipoamide_succinyltransferase_ component__E2__of_2-oxoglutarate_ dehydrogenase_complex__EC_2.3.1.61 | The oxidative decarboxylation of pyruvate and 2-0x0- glutarate is catalysed by two analogous multi-enzyme com- plexes, the pyruvate and the 2-oxoglutarate dehydrogenase complexes. | 12 |
| | Clustering-based_subsystems | Cytochrome_biogenesis | CBSS-196164.1.peg.1690 | Heme_O_synthase_protoheme_IX_ farnesyltransferase__EC_2.5.1.-__ COX10-CtaB | CtaB is a heme O synthase (protoheme IX farnesyltransferase) and while CtaA is an integral membrane protein that converts heme O to heme A. The membrane protein CtaB in *S. aureus* is protoheme IX farnesyltransferase involved in the synthesis of the heme containing terminal oxidases of bacterial respiratory chain. | 13 |

| | | | | | | |
|---|---|---|---|---|---|---|
| | Phosphorus_Metabolism | NULL | Phosphate_metabolism | NAD_P__transhydrogenase_subunit_ beta__EC_1.6.1.2_ | Pyridine nucleotide transhydrogenase (EC 1.6.1.1) is a proton pump which catalyzes the reversible reaction where reduction of NADP+ by NADH is linked to an inward translocation of protons from the periplasm (in bacteria) or cytosol (mammalian cells) into the cytosol or mitochondrial matrix, respectively. In the presence of an electrochemical proton gradient generated by another proton pump, the apparent equilibrium of the reaction is strongly shifted toward formation of NADPH and NAD+, and the rate of reduction of NADP+ by NADH is increased 10-fold | 14 |
| | Amino_Acids_and_Derivatives | Glutamine_glutamate_ aspartate_asparagine | ammonia_assimilation | Aspartate_aminotransferase | Nitrogen incorporated into glutamine by glutamine synthetase and glutamate by glutamate dehydrogenase can be redistributed to other amino acids, mainly by the action of transaminase or amino transferase as aspartate aminotransferase (AAT) which catalyzes the reversible transfer of an amino group from glutamate to oxaloacetate to form aspartate and α-ketoglutarate. | 15 |
| | Clustering-based_subsystems | NULL | Glycogen_metabolism_cluster | Glycogen_debranching_enzyme__EC_3 .2.1.-_ | Glycogen debranching enzyme (GDE) is a bifunctional enzyme exhibiting both transferase (oligo-1,4→1,4-glucan transferase, EC2.4.1.25) and glucosidase (amylo-1,6-glucosidase, EC 3.2.1.33) activities. GDE along with phosphorylase ensures the complete degradation of glycogen and the release of glucose-1-phosphate and glucose. | 16 |

| | | | | | | |
|---|---|---|---|---|---|---|
| | Metabolism_of_Aromatic_ Compounds | Anaerobic_degradation _of_aromatic_ compounds | Anaerobic_benzoate_metabolism | Glutaryl-CoA_dehydrogenase__EC_1.3.99.7_ | Glutaryl-CoA dehydrogenase (GCD) in addition to the α,β-dehydrogenation reaction, common to all ACDs, GCD catalyzes decarboxylation of glutaryl-CoA to produce CO2 and crotonyl-Co | 17 |
| | Respiration | NULL | Biogenesis_of_c-type_ cytochromes | Cytochrome_c_heme_lyase_subunit_CcmF | The critical step of cytochrome c biogenesis is the covalent attachment of the heme cofactor to the polypeptide. Two thioether bonds between the heme vinyl groups and the cysteines of a conserved sequence motif within the polypeptide, CXXCH, are formed in a stereospecific reaction referred to as heme ligation, which is catalyzed by the enzyme cytochrome c heme lyase. CcmF is a key player in the late steps of cytochrome c maturation because it contains a tryptophan-rich motif. | 18 |
| | Amino_Acids_and_Derivatives | Alanine_serine_and_ glycine | Glycine_and_Serine_Utilization | Phosphoserine_aminotransferase__EC_2.6.1.52_ | Phosphoserine aminotransferase (PSAT; EC 2.6.1.52) catalyzes the reversible conversion of 3-phospho hydroxypyru- private to L-phosphoserine. This reaction is the second step of the major pathway of L-serine biosynthesis in many organisms, from bacterial cells to mammals | 19 |
| | Amino_Acids_and_Derivatives | Alanine_serine_and_ glycine | Glycine_and_Serine_Utilization | Glycine_dehydrogenase_ [decarboxylating]__glycine_cleavage_ system_P_protein___EC_1.4.4.2_ | Glycine decarboxylase (GDC, also named glycine-cleavage-system or glycine dehydrogenase) is a multi-protein complex that occurs in all organisms, prokaryotes and eukaryotes. GDC, together with serine hydroxymethyltransferase (SHMT), is responsible for the inter-conversion of glycine and serine, an essential and ubiquitous step of primary metabolism. | 20 |

| | | | | | | |
|---|---|---|---|---|---|---|
| | Protein_Metabolism | Protein_degradation | Aminopeptidases__EC_3.4.11.-_ | Membrane_alanine_aminopeptidase_N __EC_3.4.11.2_ | Aminopeptidase N (EC 3.4.11.2) is an enzyme preferentially releasing neutral amino acids from the N- terminal end of oligopeptides. It is a type II membrane glycoprotein with a zinc-dependent catalytic activity and is expressed in many tissues, being most abundant in the enterocytes of the small intestine and in the epithelium of kidney proximal tubules. | 21 |
| | Carbohydrates | NULL | Unknown_carbohydrate_utilization ___cluster_Yeg__ | Fructose-bisphosphate_aldolase_class_I__EC_4.1.2.13_ | The glycolytic enzyme fructose bisphosphate aldolase (EC 4.1.2.13) catalyzes the aldol cleavage of fructose 1,6-bisphosphate to dihydroxyacetone phosphate and glyceraldehyde 3-phosphate. It also catalyzes the reverse reaction. The hallmark of a class I aldolase is the presence of an active-site lysine residue which forms a Schiff base with the substrate | 22 |
| | Respiration | NULL | Quinone_oxidoreductase_family | Quinone_oxidoreductase__EC_1.6.5.5_ | NADH generated in catabolic pathways is fed into energy converting electron transfer chains via NADH:quinone oxidoreductases. It is found in many eubacteria where it is frequently termed NADH dehydrogenase-1 or NDH-1. Moreover, complex I is involved in bacterial photosynthetic electron transport | 23 |
| | Clustering-based_subsystems | Putrescine/GABA_ utilization_cluster-temporalto_add_to_SSs | GABA_and_putrescine_ metabolism_from_cluters | Omega-amino_acid--pyruvate_ aminotransferase__EC_2.6.1.18_ | The enzyme catalyzes the transamination between various ω-amino acids or amines and pyruvate which is the exclusive amino acceptor. α-Amino acids except L-α-alanine are inert as amino donor. | 24 |

| | | | | | | |
|---|---|---|---|---|---|---|
| | Nucleosides_and_Nucleotides | NULL | Ribonucleotide_reduction | Ribonucleotide_reductase_of_class_Ia_ _aerobic__alpha_subunit__EC_1.17.4.1 _ | Ribonucleotide reductase (RNR), converts all four ribonucleotides into deoxyribonucleotide precursors for DNA synthesis and repair. Eukaryotes and some aerobic prokaryotes employ class Ia RNRs, which utilize a diferric-tyrosyl radical cofactor for the reduction of ribonucleoside diphosphates (NDPs) | 25 |
| | Carbohydrates | Central_carbohydrate_ metabolism | Pyruvate_metabolism_II:_ acetyl-CoA_acetogenesis_from_ pyruvate | Pyruvate_dehydrogenase_E1_ component__EC_1.2.4.1_ | Catalyses the oxidative decarboxylation of pyruvate and the subsequent acetylation of coenzyme A, with the concomitant formation of NADH. | 26 |
| | Stress_Response | Oxidative_stress | Oxidative_stress | Manganese_superoxide_dismutase__ EC_1.15.1.1_ | Mn SODs occur in mitochondria and peroxisomes.Mn SODs carry only one metal atom per subunit. These enzymes cannot function without the Mn atom present at the active site. Even though Mn and Fe SODs have a high similarity in their primary, secondary, and tertiary structure, these enzymes have diverged sufficiently that Fe (II) could not restore the activity of Mn SOD and vice versa. Catalysis by Mn SODs is through the attraction of negatively charged | 27 |
| | Stress_Response | Acid_stress | Acid_resistance_mechanisms | Biosynthetic_arginine_decarboxylase__ EC_4.1.1.19_ | Arginine decarboxylase confers acid resistance by consuming hydrogen ions while removing the carboxyl group from arginine to produce agmatine and $H_2O$. At low concentrations, arginine induces acid resistance mainly by consuming hydrogen ions, at higher concentrations, arginine induces acid resistance by both decreasing intracellular hydrogen ions and lessening acidity-induced cell wall/membrane disruption. | 28 |

| | | | | | | |
|---|---|---|---|---|---|---|
| | Respiration | Electron_accepting_ reactions | Terminal_cytochrome_C_oxidases | Cytochrome_c_oxidase_polypeptide_I_ _EC_1.9.3.1_ | Catalyzes the rate-limiting terminal step of oxidative phosphorylation at the inner mitochondrial membrane. The copper in subunit 2 of the complex transfers electrons from cytochrome c to the copper and iron-containing center of the catalytic subunit 1. A vectorial Bohr mechanism moves reduced protons from the inner aqueous space through the mitochondrial membrane and the heme-copper oxidases then release them into the external space. This proton translocation builds up a proton gradient across the inner mitochondrial membrane that drives the synthesis of ATP. | 29 |
| | Phosphorus_Metabolism | NULL | High_affinity_phosphate_ transporter_and_control_of_PHO_ regulon | Polyphosphate_kinase__EC_2.7.4.1_ | The activities of PPK include, in addition to the synthesis of poly P from ATP, the conversion of ADP back to ATP in an apparent reversal of the reaction, and the conversion of other nucleotides, especially GDP to GTP | 30 |
| | Virulence_Disease_and_ Defense | NULL | Bacterial_cyanide_production_and _tolerance_mechanisms | Thiosulfate_sulfurtransferase_ rhodanese__EC_2.8.1.1_ | Rhodanese or thiosulfate sulfurtransferase (TST) is a mitochondrial matrix enzyme that plays roles in cyanide detoxification, the formation of iron–sulfur proteins and the modification of sulfur-containing enzymes. Transsulfuration reaction catalyzed by TST is also involved in H2S detoxification. This enzyme catalyses the transfer of a sulfur atom from thiosulfate to sulfur acceptors like cyanide and thiol compounds to produce less toxic molecules, thiocyanate and persulfides, respectively | 31 |

| | | | | | | |
|---|---|---|---|---|---|---|
| | Stress_Response | Oxidative_stress | Glutathione:_Biosynthesis_and_ gamma-glutamyl_cycle | 5-oxoprolinase__EC_3.5.2.9_ | GSH is synthesized in two steps by the successive action of γ- glutamylcysteine synthetase and glutathione synthetase. γ Glutamyl transpeptidase, γ-Glutamyl transferase, γ-Glutamylcyclotransferase and 5-Oxoprolinase catalyse the degradation of GSH to its constitutive amino acids | 32 |
| | Miscellaneous | Plant-Prokaryote_DOE_project | COG3533 | L-arabonate_dehydratase__EC_ 4.2.1.25_ | catalyzes the conversion of L-arabinonate to 2-dehydro-3-deoxy-L-arabinonate. The enzyme is a tetramer consisting of a dimer of dimers, where each monomer is composed of two domains. The active site contains a catalytically important [2Fe-2S] cluster and Mg2+ ion, and is buried between two domains, and also at the dimer interface. | 33 |
| | Carbohydrates | Central_carbohydrate_ metabolism | Dehydrogenase_complexes | 2-oxoglutarate_dehydrogenase_E1_ component__EC_1.2.4.2_ | The thiamine-dependent E1o component (EC 1.2.4.2) of the 2-oxoglutarate dehydrogenase complex catalyses a rate-limiting step of the tricarboxylic acid cycle (TCA) of aerobically respiring organisms. | 34 |
| | Photosynthesis | NULL | Proteorhodopsin | Beta-carotene_ketolase__EC_1.14.-.-_ | Ketocarotenoids are derived from β-carotene by 3-hydroxylation and 4-ketolation and both ionone end groups, catalyzed by β-carotene hydroxylase and β-carotene ketolase, respectively. They are rarely found in higher plants, because the genes encoding β-carotene ketolase are restricted to some bacteria, fungi and algae. | 35 |
| | Respiration | Electron_accepting_ reactions | Terminal_cytochrome_C_oxidases | Cytochrome_c_oxidase_subunit_CcoN_ _EC_1.9.3.1_ | The cytochrome c oxidase (Cco) family related to oxidation-reduction was also inhibited in response to iron limitation, which is comprised of four subunits, CcoN, CcoO, and CcoQ, which act as the terminal enzyme of the respiratory chain. | 36 |

| | | | | | | |
|---|---|---|---|---|---|---|
| | Metabolism_of_Aromatic_Compounds | Metabolism_of_central_aromatic_intermediates | Homogentisate_pathway_of_ aromatic_compound_degradation | Homogentisate_12-dioxygenase__EC_1.13.11.5_ | Homogentisate 1,2-dioxygenase (HGDO) uses a mononuclear nonheme $Fe^{2+}$ to catalyze the oxidative ring cleavage in the degradation of Tyr and Phe by producing maleylacetoacetate from homogentisate (2,5-dihydroxyphenyl acetate) | 37 |
| | Miscellaneous | Plant-Prokaryote_DOE_project | At3g50560 | Glutathione_S-transferase__EC_2.5.1.18_ | GlutathioneS-transferases (GSTs) play an important role in higher eukaryotes in the binding, transformation, and detoxi-fication of a wide variety of both endogenous and exogenous electrophilic compounds. While in some proteobacteria, glutathione itself and glutathione-dependent enzymes, among which are GSTs, are involved in a stunning variety of metabolic processes and afford protection against oxidative stress but also ensure the correct folding, synthesis, regulation, and degradation of enzymes and multienzyme complexes | 38 |
| | Respiration | Electron_donating_ reactions | CO_Dehydrogenase | carbon_monoxide_dehydrogenase_D _protein | The anaerobic enzyme has an iron-sulfur type spectrum, which is changed in the presence of the substrate, CO. In the presence of oxygen, which destroys the activity or $CO_2$, the spectrum is that of a typical iron-sulfur protein. | 39 |
| | Protein_Metabolism | Protein_processing_and_modification | G3E_family_of_P-loop_GTPases__metallocenter_biosynthesis_ | Urease_alpha_subunit__EC_3.5.1.5_ | Urea amidohydrolase (urease) is a nickel-containing enzyme that catalyzes the hydrolysis of urea to produce ammonia and carbon dioxide in the last step of nitrogen mineralization. The β-subunit contains the active site, while the α-subunit assists the catalytic activity. | 40 |

| | | | | | | |
|---|---|---|---|---|---|---|
| | Carbohydrates | CO2_fixation | Photorespiration__oxidative_C2_cycle_ | Malate_synthase__EC_2.3.3.9_ | In the glyoxylate cycle, enzymes of the tricarboxylic acid cycle together with the first key enzyme of the cycle, isocitrate lyase, oxidize acetyl-CoA to glyoxylate. Malate synthase, the second key enzyme, catalyzes a one-step condensation of acetyl-CoA and glyoxylate to malate, the final product of the glyoxylate cycle | 41 |
| | Fatty_Acids_Lipids_and_ Isoprenoids | NULL | Polyhydroxybutyrate_metabolism | Polyhydroxyalkanoic_acid_synthase | PHA synthases catalyze the polymerization of hydroxyacyl thioesters (hydroxyacyl coenzyme A [HACoA]) into PHA with the release of CoA. This key enzyme is required regardless of the pathway used by the organism to generate HACoA substrates. | 42 |
| | Carbohydrates | Central_carbohydrate_ metabolism | Glyoxylate_bypass | Isocitrate_lyase__EC_4.1.3.1_ | The glyoxylate cycle is an anaplerotic pathway of the tricarboxylic acid (TCA) cycle that allows growth on C2 compounds by bypassing the CO2-generating steps of the TCA cycle. The unique enzymes of this route are isocitrate lyase (ICL) and malate synthase (MS). ICL cleaves isocitrate to glyoxylate and succinate, and MS converts glyoxylate and acetyl-CoA to malate. | 43 |
| | Clustering-based_subsystems | NULL | CBSS-316057.3.peg.563 | Cytochrome_c_oxidase_polypeptide_III __EC_1.9.3.1_ | Mitochondrial cytochrome c oxidase and its bacterial homologs catalyze electron transfer and proton translocation reactions across membranes. Antibody and chemical-labelling experiments suggest that subunits I-III and most (but not all) of the nuclear-encoded subunits span the inner mitochondrial membrane. Subunit III and most of the nuclear subunits are essential for the assembly of a functional catalytic enzyme. | 44 |

| | | | | | | |
|---|---|---|---|---|---|---|
| | Cofactors_Vitamins_Prosthetic_Groups_Pigments | Tetrapyrroles | Heme_and_Siroheme_ Biosynthesis | Coproporphyrinogen_III_oxidase_ aerobic__EC_1.3.3.3_ | The aerobic coproporphyrinogen III oxidase requires molecular oxygen as an electron acceptor for the oxidative decarboxylation of coproporphyrinogen III, as described for animals, yeasts, and aerobically grown bacteria. | 45 |
| | Protein_Metabolism | Protein_processing_and_modification | Protein_Acetylation_and_ Deacetylation_in_Bacteria | Acetate_permease_ActP__ cation/acetate_symporter_ | The ActP transporter belongs to the Sodium:Solute Symporter Family and is highly specific for short chain aliphatic monocarboxylates, namely acetate, glycolate and propionate. | 46 |
| | Clustering-based_subsystems | Molybdopterin_ oxidoreductase | CBSS-269799.3.peg.2220 | Molybdopterin_oxidoreductase__EC_1.2.7.-_ | Finding suggests that MopABCD may not function as a hydrogenase or formate dehydrogenase but that, rather, defects in H2 and formate oxidation are more likely to be due to electron transfer chain interruption. | 47 |
| | Carbohydrates | Fermentation | Acetyl-CoA_fermentation_to_ Butyrate | Electron_transfer_flavoprotein- ubiquinone_oxidoreductase__EC_1.5.5.1_ | Electron transfer flavoprotein:ubiquinone oxidoreductase (ETF-QO) is a component of the mitochondrial respiratory chain that together with electron transfer flavoprotein (ETF) forms a short pathway that transfers electrons from 11 different mitochondrial flavoprotein dehydrogenases to the ubiquinone pool. | 48 |

| | | | | | | |
|---|---|---|---|---|---|---|
| | Respiration | Electron_donating_ reactions | CO_Dehydrogenase | Carbon_monoxide_dehydrogenase_ large_chain__EC_1.2.99.2_ | Carbon monoxide dehydrogenases (CODHs), operative in anaerobic and aerobic organisms, respectively. It has been estimated that each year 200–600 million tonnes of atmospheric CO are converted into CO2 by microbial activity. This process contributes to the global carbon cycle and helps maintain atmospheric CO levels below those toxic to respiring organisms, including humans. Acetogens also contribute to the carbon cycle and are estimated to produce over 10 billion tonnes of acetic acid annually. | 49 |
| | Virulence_Disease_and_Defen se | Resistance_to_ antibiotics_and_toxic_ compounds | Resistance_to_chromium_ compounds | Chromate_transport_protein_ChrA | The chrA gene of *Pseudomonas aeruginosa* plasmid pUM505 encodes the hydrophobic protein ChrA, which confers resistance to chromate by the energy-dependent efflux of chromate ions | 50 |
| | Stress_Response | Oxidative_stress | Glutathione:_Biosynthesis_and_ gamma-glutamyl_cycle | Glutamate--cysteine_ligase__EC_6.3.2.2_ | Glutathione synthesis occurs in two ATP-dependent steps. In the first reaction, glutamate-cysteine ligase (GCL) (EC 6.3.2.2) catalyzes the formation of the dipeptide γ-glutamylcysteine from cysteine and glutamate. Addition of glycine to the dipeptide occurs in a second reaction, catalyzed by glutathione synthetase. Of the two enzymes, GCL appears to be rate-limiting | 51 |

| | | | | | | |
|---|---|---|---|---|---|---|
| | Amino_Acids_and_Derivatives | Aromatic_amino_acids_ and_derivatives | Aromatic_amino_acid_i nterconversions_with_aryl_acids | Indolepyruvate_ferredoxin_ oxidoreductase_alpha_and_beta_ subunits | IOR oxidizes aryl pyruvates such as indolepyruvate, phenylpyruvate, and p-hydroxyphenylpyruvate, which originate from the aromatic amino acid tryptophan, phenylalanine and tyrosine, respectively, through the reaction of aromatic aminotransferases. The genes iorA and iorB which encode respective α and β subunits have been cloned and sequenced from Pyrococcus kodakaraensis KOD. | 52 |
| | Miscellaneous | Plant-Prokaryote_DOE_project | Niacin-Choline_transport_and_ metabolism | Choline_dehydrogenase__EC_1.1.99.1_ | Choline dehydrogenase (EC 1.1.99.1) catalyzes the four-electron oxidation of choline to glycine-betaine via a betaine-aldehyde intermediate. Such a reaction is of considerable interest for biotechnological applications in that transgenic plants engineered with bacterial glycine-betaine-synthesizing enzymes have been shown to have enhanced tolerance towards various environmental stresses, such as hypersalinity, freezing, and high temperatures | 53 |
| | Respiration | Electron_accepting_ reactions | Terminal_cytochrome_C_oxidases | Alternative_cytochrome_c_oxidase_ polypeptide_CoxN__EC_1.9.3.1_ | The CoxN gene encoded a polypeptide with about 40% sequence identity with subunit I representatives including the groups were strictly conserved. A B. japonicum coxN mutant exhibited no obvious defects in free-living, aerobic growth or in root nodule symbiosis with soybean. CoxN gene is not essential for respiration in the N2 fixing bacteroid. | 54 |

| | | | | | | |
|---|---|---|---|---|---|---|
| | Metabolism_of_Aromatic_ Compounds/ | NULL | Benzoate_transport_and_ degradation_cluster | Benzoyl-CoA_oxygenase_component_B | A novel aerobic benzoate pathway has recently been discovered in various bacteria in which benzoate is first converted to benzoyl CoA. Benzoyl-CoA is oxidized to 2,3-dihydro-2,3-dihydroxybenzoyl-CoA (benzoyl-CoA dihydrodiol) by benzoyl-CoA oxygenase/reductase BoxBA in the presence of molecular oxygen. | 55 |
| | Metabolism_of_Aromatic_ Compounds | NULL | Benzoate_transport_and_ degradation_cluster | benzoyl-CoA-dihydrodiol_lyase__BoxC | BoxC catalyses the reaction 2,3-dihydro 2,3 dihydroxybenzoyl-CoA + H2O →3,4 dehydroadipyl CoA semialdehyde + HCOOH. | 55 |

References


1. Louie, T. M., Xie, X. S. & Xun, L. Coordinated Production and Utilization of FADH2 by NAD(P)H−Flavin Oxidoreductase and 4-Hydroxyphenylacetate 3-Monooxygenase. *Biochemistry* **42**, 7509–7517 (2003).
2. Watanabe, M., Nameki, N., Matsuo-Takasaki, M., Nishimura, S. & Okada, N. tRNA recognition of tRNA-guanine transglycosylase from a hyperthermophilic archaeon, Pyrococcus horikoshii. *J. Biol. Chem.* **276**, 2387–2394 (2001).
3. Ernst, F. D. *et al.* The nickel-responsive regulator NikR controls activation and repression of gene transcription in Helicobacter pylori. *Infect. Immun.* **73**, 7252–7258 (2005).
4. Anand, N. & Sharma†, S. *Approaches to Design and Synthesis of Antiparasitic Drugs*. (Elsevier, 1997).
5. Mittenhuber, G. Comparative Genomics of Prokaryotic GTP-Binding Proteins (the Era, Obg, EngA, ThdF (TrmE), YchF and YihA Families) and their Relationship to Eukaryotic GTP Binding Proteins (the DRG, ARF, RAB, RAN, RAS and RHO Families). *J. Mol. Microbiol. Biotechnol.* **3**, 21–35 (2001).
6. Zhu, H. & Shuman, S. A primer-dependent polymerase function of pseudomonas aeruginosa ATP-dependent DNA ligase (LigD). *J. Biol. Chem.* **280**, 418–427 (2005).
7. Hanson, P. I. & Whiteheart, S. W. AAA+ proteins: have engine, will work. *Nat. Rev. Mol. Cell Biol.* **6**, 519–529 (2005).
8. Luengo, J. M., García, J. L. & Olivera, E. R. The phenylacetyl-CoA catabolon: a complex catabolic unit with broad biotechnological applications. *Mol. Microbiol.* **39**, 1434–1442 (2001).
9. Ferrández, A., García, J. L. & Díaz, E. Transcriptional Regulation of the Divergent paaCatabolic Operons for Phenylacetic Acid Degradation inEscherichia coli *. *J. Biol. Chem.* **275**, 12214–12222 (2000).

10. Sardiello, M., Annunziata, I., Roma, G. & Ballabio, A. Sulfatases and sulfatase modifying factors: an exclusive and promiscuous relationship. *Hum. Mol. Genet.* **14**, 3203–3217 (2005).

11. Tabatabai, M. A. & Bremner, J. M. Arylsulfatase activity of soils. *Soil Sci. Soc. Am. J.* **34**, 225–229 (1970).

12. Spencer, M. E., Darlison, M. G., Stephens, P. E., Duckenfield, I. K. & Guest, J. R. Nucleotide sequence of the sucB gene encoding the dihydrolipoamide succinyltransferase of Escherichia coli K12 and homology with the corresponding acetyltransferase. *European Journal of Biochemistry* vol. 141 361–374 (1984).

13. Xu, T. *et al.* Absence of Protoheme IX Farnesyltransferase CtaB Causes Virulence Attenuation but Enhances Pigment Production and Persister Survival in MRSA. *Frontiers in Microbiology* vol. 7 (2016).

14. Glavas, N. A., Hou, C. & Bragg, P. D. Involvement of histidine-91 of the beta subunit in proton translocation by the pyridine nucleotide transhydrogenase of Escherichia coli. *Biochemistry* **34**, 7694–7702 (1995).

15. Mrid, R. B. & Ben Mrid, R. Effect of Nitrogen Source and Concentration on Growth and Activity of Nitrogen Assimilation Enzymes in Roots of a Moroccan Sorghum Ecotype. *Plant* vol. 4 71 (2016).

16. Nakayama, A., Yamamoto, K. & Tabata, S. Identification of the catalytic residues of bifunctional glycogen debranching enzyme. *J. Biol. Chem.* **276**, 28824–28828 (2001).

17. Wang, M. *et al.* The crystal structure and mechanism of human glutaryl-CoA dehydrogenase. (2004) doi:10.2210/pdb1sir/pdb.

18. Ren, Q., Ahuja, U. & Thöny-Meyer, L. A Bacterial Cytochrome c Heme Lyase. *Journal of Biological Chemistry* vol. 277 7657–7663 (2002).

19. Dubnovitsky, A. P., Kapetaniou, E. G. & Papageorgiou, A. C. Enzyme adaptation to alkaline pH: Atomic resolution (1.08 Å) structure of phosphoserine aminotransferase from Bacillus alcalophilus. *Protein Sci.* **14**, 97–110 (2005).

20. Bauwe, H. Genetic manipulation of glycine decarboxylation. *Journal of Experimental Botany* vol. 54 1523–1535 (2003).

21. Sjöström, H., Norén, O. & Olsen, J. Structure and Function of Aminopeptidase N. in *Cellular Peptidases in Immune Functions and Diseases 2* (eds. Langner, J. & Ansorge, S.) 25–34 (Springer US, 2002). doi:10.1007/0-306-46826-3_2.

22. Seattle Structural Genomics Center for Infectious Disease. Crystal structure of fructose bisphosphate aldolase from Encephalitozoon cuniculi, bound to fructose 1,6-bisphosphate. (2010) doi:10.2210/pdb3mbf/pdb.

23. Brandt, U. Energy converting NADH:quinone oxidoreductase (complex I). *Annu. Rev. Biochem.* **75**, 69–92 (2006).

24. Yonaha, K., Toyama, S., Yasuda, M. & Soda, K. Properties of Crystalline ω-Amino Acid : Pyruvate Aminotransferase of Pseudomonas sp. F–126. *Agric. Biol. Chem.* **41**, 1701–1706 (1977).

25. Brignole, E. J., Ando, N., Zimanyi, C. M. & Drennan, C. L. The prototypic class Ia ribonucleotide reductase from Escherichia coli: still surprising after all these years. *Biochem. Soc. Trans.* **40**, 523–530 (2012).

26. Wallis, N. G. & Perham, R. N. Structural dependence of post-translational modification and reductive acetylation of the lipoyl domain of the pyruvate dehydrogenase multienzyme complex. *J. Mol. Biol.* **236**, 209–216 (1994).

27. Alscher, R. G., Erturk, N. & Heath, L. S. Role of superoxide dismutases (SODs) in controlling oxidative stress in plants. *J. Exp. Bot.* **53**, 1331–1341 (2002).

28. Gong, Z. *et al.* Arginine- and Polyamine-Induced Lactic Acid Resistance in Neisseria gonorrhoeae. *PLoS One* **11**, e0147637 (2016).

29. Kohlmeier, M. *Nutrient Metabolism: Structures, Functions, and Genes*. (Academic Press, 2015).

30. Kornberg, A., Rao, N. N. & Ault-Riché, D. Inorganic polyphosphate: a molecule of many functions. *Annu. Rev. Biochem.* **68**, 89–125 (1999).

31. Billaut-Laden, I. *et al.* Evidence for a functional genetic polymorphism of the human thiosulfate sulfurtransferase (Rhodanese), a cyanide and H2S detoxification enzyme. *Toxicology* **225**, 1–11 (2006).

32. Richie, J. P., Jr. The role of glutathione in aging and cancer. *Exp. Gerontol.* **27**, 615–626 (1992).

33. Rahman, M. M. *et al.* The Crystal Structure of a Bacterial l-Arabinonate Dehydratase Contains a [2Fe-2S] Cluster. *ACS Chem. Biol.* **12**, 1919–1927 (2017).

34. Frank, R. A. W., Price, A. J., Northrop, F. D., Perham, R. N. & Luisi, B. F. Crystal structure of the E1 component of the Escherichia coli 2-oxoglutarate dehydrogenase multienzyme complex. *J. Mol. Biol.* **368**, 639–651 (2007).

35. Sakuta, M. & Ohmiya, A. Pigment Biosynthesis II: Betacyanins and Carotenoids. *Plant Metabolism and Biotechnology* 343–371 (2011) doi:10.1002/9781119991311.ch13.

36. Zughaier, S. M. & Cornelis, P. Editorial: Role of Iron in Bacterial Pathogenesis. *Frontiers in Cellular and Infection Microbiology* vol. 8 (2018).

37. Jeoung, J. H., Bommer, M. & Lin, T. Y. Visualizing the substrate-, superoxo-, alkylperoxo-, and product-bound states at the nonheme Fe (II) site of homogentisate dioxygenase. *Proceedings of the* (2013).

38. Vuilleumier, S. Bacterial glutathione S-transferases: what are they good for? *J. Bacteriol.* **179**, 1431–1441 (1997).

39. Ragsdale, S. W., Clark, J. E., Ljungdahl, L. G., Lundie, L. L. & Drake, H. L. Properties of purified carbon monoxide dehydrogenase from Clostridium thermoaceticum, a nickel, iron-sulfur protein. *J. Biol. Chem.* **258**, 2364–2369 (1983).

40. Estiu, G. & Merz, K. M., Jr. Catalyzed decomposition of urea. Molecular dynamics simulations of the binding of urea to urease. *Biochemistry* **45**, 4429–4443 (2006).

41. Borjian, F. *et al.* Malate Synthase and β-Methylmalyl Coenzyme A Lyase Reactions in the Methylaspartate Cycle in Haloarcula hispanica. *J. Bacteriol.* **199**, (2017).

42. McCool, G. J. & Cannon, M. C. PhaC and PhaR are required for polyhydroxyalkanoic acid synthase activity in Bacillus megaterium. *J. Bacteriol.* **183**, 4235–4243 (2001).

43. Dunn, M. F., Ramírez-Trujillo, J. A. & Hernández-Lucas, I. Major roles of isocitrate lyase and malate synthase in bacterial and fungal pathogenesis. *Microbiology* **155**, 3166–3175 (2009).

44. Cooper, C. E., Nicholls, P. & Freedman, J. A. Cytochrome c oxidase: structure, function, and membrane topology of the polypeptide subunits. *Biochem. Cell Biol.* **69**, 586–607 (1991).

45. Troup, B., Jahn, M., Hungerer, C. & Jahn, D. Isolation of the hemF operon containing the gene for the Escherichia coli aerobic coproporphyrinogen III oxidase by in vivo complementation of a yeast HEM13 mutant. *J. Bacteriol.* **176**, 673–680 (1994).

46. Sá-Pessoa, J. *et al.* SATP (YaaH), a succinate–acetate transporter protein in Escherichia coli. *Biochem. J* **454**, 585–595 (2013).

47. Li, X. *et al.* A molybdopterin oxidoreductase is involved in H2 oxidation in Desulfovibrio desulfuricans G20. *J. Bacteriol.* **191**, 2675–2682 (2009).

48. Watmough, N. J. & Frerman, F. E. The electron transfer flavoprotein: ubiquinone oxidoreductases. *Biochim. Biophys. Acta* **1797**, 1910–1916 (2010).

49. Gourlay, C., Nielsen, D. J., Evans, D. J., White, J. M. & Young, C. G. Models for aerobic carbon monoxide dehydrogenase: synthesis, characterization and reactivity of paramagnetic Mo VO (μ-S) Cu I complexes. *Chem. Sci.* **9**, 876–888 (2018).

50. Aguilera, S. *et al.* Essential residues in the chromate transporter ChrA of Pseudomonas aeruginosa. *FEMS Microbiol. Lett.* **232**, 107–112 (2004).

51. Jez, J. M., Cahoon, R. E. & Chen, S. Arabidopsis thaliana glutamate-cysteine ligase: functional properties, kinetic mechanism, and regulation of activity. *J. Biol. Chem.* **279**, 33463–33470 (2004).

52. Siddiqui, M. A., Fujiwara, S., Takagi, M. & Imanaka, T. In vitro heat effect on heterooligomeric subunit assembly of thermostable indolepyruvate ferredoxin oxidoreductase. *FEBS Lett.* **434**, 372–376 (1998).

53. Gadda, G. & McAllister-Wilkins, E. E. Cloning, expression, and purification of choline dehydrogenase from the moderate halophile Halomonas elongata. *Appl. Environ. Microbiol.* **69**, 2126–2132 (2003).

54. Hennecke, H. The Role of Respiration in Symbiotic Nitrogen Fixation. *New Horizons in Nitrogen Fixation* 55–64 (1993) doi:10.1007/978-94-017-2416-6_6.

55. Gescher, J., Eisenreich, W., Wörth, J., Bacher, A. & Fuchs, G. Aerobic benzoyl-CoA catabolic pathway inAzoarcus evansii: studies on the non-oxygenolytic ring cleavage enzyme. *Molecular*

*Microbiology* vol. 56 1586–1600 (2005).

Table S5: Taxonomically unique genera identified by the Venn Diagram analysis for each zone

## (235 to 210 cm core depth): cool freshwater lake

| ID | Domain | Phylum | Class | Order | Family | Genus |
|---|---|---|---|---|---|---|
| sp579 | Archaea | Euryarchaeota | Halobacteria | Halobacteriales | Halobacteriaceae | *Natronomonas* |
| sp676 | Archaea | Euryarchaeota | Thermoplasmata | Thermoplasmatales | Picrophilaceae | *Picrophilus* |
| sp263 | Bacteria | Cyanobacteria | unclassified (derived from Cyanobacteria) | Nostocales | Nostocaceae | *Cylindrospermopsis* |
| sp423 | Bacteria | Firmicutes | Erysipelotrichi | Erysipelotrichales | Erysipelotrichaceae | *Holdemania* |
| sp429 | Bacteria | Aquificae | Aquificae (class) | Aquificales | Aquificaceae | *Hydrogenobaculum* |
| sp435 | Bacteria | Proteobacteria | Gammaproteobacteria | Alteromonadales | Idiomarinaceae | *Idiomarina* |
| sp439 | Bacteria | Fusobacteria | Fusobacteria (class) | Fusobacteriales | Fusobacteriaceae | *Ilyobacter* |
| sp443 | Bacteria | Actinobacteria | Actinobacteria (class) | Actinomycetales | Intrasporangiaceae | *Janibacter* |
| sp486 | Bacteria | Proteobacteria | Betaproteobacteria | Burkholderiales | Burkholderiaceae | *Limnobacter* |
| sp499 | Bacteria | Proteobacteria | Alphaproteobacteria | Rhodospirillales | Rhodospirillaceae | *Magnetospirillum* |
| sp515 | Bacteria | Tenericutes | Mollicutes | Entomoplasmatales | Entomoplasmataceae | *Mesoplasma* |
| sp647 | Bacteria | Bacteroidetes | Sphingobacteria | Sphingobacteriales | Sphingobacteriaceae | *Pedobacter* |
| sp671 | Bacteria | Proteobacteria | Alphaproteobacteria | Rhizobiales | Phyllobacteriaceae | *Phyllobacterium* |
| sp682 | Bacteria | Planctomycetes | Planctomycetacia | Planctomycetales | Planctomycetaceae | *Planctomyces* |
| sp685 | Bacteria | Proteobacteria | Deltaproteobacteria | Myxococcales | Nannocystaceae | *Plesiocystis* |
| sp771 | Bacteria | Proteobacteria | Gammaproteobacteria | Enterobacteriales | Enterobacteriaceae | *Salmonella* |
| sp831 | Bacteria | Proteobacteria | Epsilonproteobacteria | unclassified (derived from Epsilonproteobacteria) | unclassified (derived from Epsilonproteobacteria) | *Sulfurovum* |
| sp924 | Bacteria | Proteobacteria | Gammaproteobacteria | Enterobacteriales | Enterobacteriaceae | *Yersinia* |
| sp129 | Eukaryota | Nematoda | Chromadorea | Rhabditida | Rhabditidae | *Caenorhabditis* |
| sp168 | Eukaryota | Chordata | Mammalia | Carnivora | Canidae | *Canis* |
| sp178 | Eukaryota | Chlorophyta | Ulvophyceae | Bryopsidales | Caulerpaceae | *Caulerpa* |
| sp223 | Eukaryota | Streptophyta | Coleochaetophyceae | Coleochaetales | Coleochaetaceae | *Coleochaete* |
| sp255 | Eukaryota | unclassified (derived from Eukaryota) | Bangiophyceae | Cyanidiales | Cyanidiaceae | *Cyanidioschyzon* |
| sp329 | Eukaryota | Streptophyta | Gnetopsida | Ephedrales | Ephedraceae | *Ephedra* |
| sp571 | Eukaryota | Ascomycota | Sordariomycetes | Hypocreales | unclassified (derived from Hypocreales) | *Myrothecium* |
| sp697 | Eukaryota | unclassified (derived from Eukaryota) | Bangiophyceae | Porphyridiales | Porphyridiaceae | *Porphyridium* |

| | | | | | | |
|---|---|---|---|---|---|---|
| sp716 | Eukaryota | Chlorophyta | Prasinophyceae | Pseudoscourfieldiales | Pycnococcaceae | *Pseudoscourfieldia* |
| sp725 | Eukaryota | Phaeophyceae | unclassified (derived from Phaeophyceae) | Ectocarpales | Acinetosporaceae | *Pylaiella* |
| sp884 | Eukaryota | Euglenida | unclassified (derived from Euglenida) | Euglenales | unclassified (derived from Euglenales) | *Trachelomonas* |
| sp896 | Eukaryota | Nemertea | Anopla | Paleonemertea | Tubulanidae | *Tubulanus* |
| sp763 | Viruses | unclassified (derived from Viruses) | unclassified (derived from Viruses) | Caudovirales | Siphoviridae | *SPbeta-like viruses* |
| sp952 | Viruses | unclassified (derived from Viruses) | unclassified (derived from Viruses) | Caudovirales | Myoviridae | *unclassified (derived from Myoviridae)* |

## (185 to 60 cm core depth): warm hyposaline lake

| ID | Domain | Phylum | Class | Order | Family | Genus |
|---|---|---|---|---|---|---|
| sp157 | Archaea | Euryarchaeota | unclassified (derived from Euryarchaeota) | unclassified (derived from Euryarchaeota) | unclassified (derived from Euryarchaeota) | *Candidatus Parvarchaeum* |
| sp396 | Archaea | Euryarchaeota | Halobacteria | Halobacteriales | Halobacteriaceae | *Halobacterium* |
| sp397 | Archaea | Euryarchaeota | Halobacteria | Halobacteriales | Halobacteriaceae | *Haloferax* |
| sp127 | Bacteria | Firmicutes | Clostridia | Clostridiales | Lachnospiraceae | *Butyrivibrio* |
| sp177 | Bacteria | Firmicutes | Clostridia | Clostridiales | Lachnospiraceae | *Catonella* |
| sp231 | Bacteria | Firmicutes | Erysipelotrichi | Erysipelotrichales | Erysipelotrichaceae | *Coprobacillus* |
| sp241 | Bacteria | Chlamydiae | Chlamydiae (class) | Chlamydiales | Criblamydiaceae | *Criblamydia* |
| sp268 | Bacteria | Bacteroidetes | Cytophagia | Cytophagales | Cytophagaceae | *Cytophaga* |
| sp311 | Bacteria | Bacteroidetes | Cytophagia | Cytophagales | Cytophagaceae | *Dyadobacter* |
| sp327 | Bacteria | Tenericutes | Mollicutes | Entomoplasmatales | Entomoplasmataceae | *Entomoplasma* |
| sp355 | Bacteria | Firmicutes | Clostridia | Clostridiales | Clostridiales Family XI. Incertae Sedis | *Finegoldia* |
| sp364 | Bacteria | Proteobacteria | Alphaproteobacteria | Rhizobiales | Aurantimonadaceae | *Fulvimarina* |
| sp366 | Bacteria | Fusobacteria | Fusobacteria (class) | Fusobacteriales | Fusobacteriaceae | *Fusobacterium* |
| sp378 | Bacteria | Proteobacteria | Gammaproteobacteria | Alteromonadales | Alteromonadaceae | *Glaciecola* |
| sp394 | Bacteria | Proteobacteria | Deltaproteobacteria | Myxococcales | Haliangiaceae | *Haliangium* |
| sp412 | Bacteria | Firmicutes | Clostridia | Clostridiales | Heliobacteriaceae | *Heliobacterium* |
| sp433 | Bacteria | Proteobacteria | Alphaproteobacteria | Rhodobacterales | Hyphomonadaceae | *Hyphomonas* |
| sp455 | Bacteria | Bacteroidetes | Flavobacteria | Flavobacteriales | Flavobacteriaceae | *Kordia* |
| sp457 | Bacteria | Proteobacteria | Alphaproteobacteria | Rhodospirillales | Acetobacteraceae | *Kozakia* |
| sp493 | Bacteria | Proteobacteria | Betaproteobacteria | Neisseriales | Neisseriaceae | *Lutiella* |

| | | | | | | |
|---|---|---|---|---|---|---|
| sp498 | Bacteria | Proteobacteria | unclassified (derived from Proteobacteria) | unclassified (derived from Proteobacteria) | unclassified (derived from Proteobacteria) | *Magnetococcus* |
| sp511 | Bacteria | Deinococcus-Thermus | Deinococci | Thermales | Thermaceae | *Meiothermus* |
| sp545 | Bacteria | Proteobacteria | Betaproteobacteria | Methylophilales | Methylophilaceae | *Methylotenera* |
| sp554 | Bacteria | Actinobacteria | Actinobacteria (class) | Actinomycetales | Micromonosporaceae | *Micromonospora* |
| sp557 | Bacteria | Firmicutes | Negativicutes | Selenomonadales | Veillonellaceae | *Mitsuokella* |
| sp584 | Bacteria | Proteobacteria | Alphaproteobacteria | Rhodospirillales | Acetobacteraceae | *Neoasaia* |
| sp611 | Bacteria | Proteobacteria | Alphaproteobacteria | Rhodobacterales | Rhodobacteraceae | *Octadecabacter* |
| sp635 | Bacteria | Proteobacteria | Alphaproteobacteria | Rhodobacterales | Rhodobacteraceae | *Paracoccus* |
| sp638 | Bacteria | Proteobacteria | Alphaproteobacteria | Rhizobiales | Phyllobacteriaceae | *Parvibaculum* |
| sp639 | Bacteria | Proteobacteria | Gammaproteobacteria | Pasteurellales | Pasteurellaceae | *Pasteurella* |
| sp654 | Bacteria | Chlorobi | Chlorobia | Chlorobiales | Chlorobiaceae | *Pelodictyon* |
| sp679 | Bacteria | Planctomycetes | Planctomycetacia | Planctomycetales | Planctomycetaceae | *Pirellula* |
| sp689 | Bacteria | Proteobacteria | Betaproteobacteria | Burkholderiales | Comamonadaceae | *Polaromonas* |
| sp698 | Bacteria | Bacteroidetes | Bacteroidia | Bacteroidales | Porphyromonadaceae | *Porphyromonas* |
| sp734 | Bacteria | Proteobacteria | Gammaproteobacteria | unclassified (derived from Gammaproteobacteria) | unclassified (derived from Gammaproteobacteria) | *Reinekea* |
| sp754 | Bacteria | Proteobacteria | Alphaproteobacteria | Rhodospirillales | Acetobacteraceae | *Roseomonas* |
| sp759 | Bacteria | Actinobacteria | Actinobacteria (class) | Rubrobacterales | Rubrobacteraceae | *Rubrobacter* |
| sp776 | Bacteria | Actinobacteria | Actinobacteria (class) | Bifidobacteriales | Bifidobacteriaceae | *Scardovia* |
| sp798 | Bacteria | Proteobacteria | Deltaproteobacteria | Myxococcales | Polyangiaceae | *Sorangium* |
| sp822 | Bacteria | Actinobacteria | Actinobacteria (class) | Actinomycetales | Streptosporangiaceae | *Streptosporangium* |
| sp829 | Bacteria | Proteobacteria | Epsilonproteobacteria | Campylobacterales | Helicobacteraceae | *Sulfurimonas* |
| sp833 | Bacteria | Firmicutes | Clostridia | Clostridiales | Clostridiales Family XVIII. Incertae Sedis | *Symbiobacterium* |
| sp876 | Bacteria | Proteobacteria | Betaproteobacteria | Hydrogenophilales | Hydrogenophilaceae | *Thiobacillus* |
| sp877 | Bacteria | Proteobacteria | Gammaproteobacteria | Thiotrichales | Piscirickettsiaceae | *Thiomicrospira* |
| sp878 | Bacteria | Proteobacteria | Betaproteobacteria | Burkholderiales | unclassified (derived from Burkholderiales) | *Thiomonas* |
| sp886 | Bacteria | Spirochaetes | Spirochaetes (class) | Spirochaetales | Spirochaetaceae | *Treponema* |
| sp894 | Bacteria | Actinobacteria | Actinobacteria (class) | Actinomycetales | Tsukamurellaceae | *Tsukamurella* |
| sp918 | Bacteria | Proteobacteria | Alphaproteobacteria | Rhizobiales | Xanthobacteraceae | *Xanthobacter* |
| sp926 | Bacteria | Bacteroidetes | Flavobacteria | Flavobacteriales | Flavobacteriaceae | *Zunongwangia* |
| sp936 | Bacteria | unclassified (derived from Bacteria) | unclassified (derived from Bacteria) | unclassified (derived from Bacteria) | unclassified (derived from Bacteria) | unclassified (derived from Bacteria) |
| sp949 | Bacteria | Proteobacteria | Gammaproteobacteria | unclassified (derived from Gammaproteobacteria) | unclassified (derived from Gammaproteobacteria) | unclassified (derived from Gammaproteobacteria) |

| | | | | | | |
|---|---|---|---|---|---|---|
| sp961 | Bacteria | Firmicutes | Clostridia | Clostridiales | Ruminococcaceae | unclassified (derived from Ruminococcaceae) |
| sp962 | Bacteria | Chlamydiae | Chlamydiae (class) | Chlamydiales | Simkaniaceae | unclassified (derived from Simkaniaceae) |
| sp136 | Eukaryota | Streptophyta | unclassified (derived from Streptophyta) | Laurales | Calycanthaceae | *Calycanthus* |
| sp139 | Eukaryota | Ascomycota | Saccharomycetes | Saccharomycetales | unclassified (derived from Saccharomycetales) | *Candida* |
| sp184 | Eukaryota | Streptophyta | unclassified (derived from Streptophyta) | Ceratophyllales | Ceratophyllaceae | *Ceratophyllum* |
| sp199 | Eukaryota | Chlorophyta | Chlorophyceae | Chlamydomonadales | Haematococcaceae | *Chlorogonium* |
| sp212 | Eukaryota | Chordata | Ascidiacea | Enterogona | Cionidae | *Ciona* |
| sp219 | Eukaryota | Streptophyta | Zygnemophyceae | Desmidiales | Closteriaceae | *Closterium* |
| sp236 | Eukaryota | Ascomycota | Sordariomycetes | Hypocreales | Cordycipitaceae | *Cordyceps* |
| sp245 | Eukaryota | Streptophyta | Liliopsida | Pandanales | Stemonaceae | *Croomia* |
| sp254 | Eukaryota | Streptophyta | unclassified (derived from Streptophyta) | Solanales | Convolvulaceae | *Cuscuta* |
| sp259 | Eukaryota | unclassified (derived from Eukaryota) | Glaucocystophyceae | unclassified (derived from Glaucocystophyceae) | unclassified (derived from Glaucocystophyceae) | *Cyanoptyche* |
| sp261 | Eukaryota | Nematoda | Chromadorea | Rhabditida | Strongylidae | *Cylicocyclus* |
| sp279 | Eukaryota | Arthropoda | Arachnida | Ixodida | Ixodidae | *Dermacentor* |
| sp299 | Eukaryota | unclassified (derived from Eukaryota) | unclassified (derived from Eukaryota) | Dictyosteliida | unclassified (derived from Dictyosteliida) | *Dictyostelium* |
| sp328 | Eukaryota | Streptophyta | Zygnemophyceae | Zygnematales | Zygnemataceae | *Entransia* |
| sp341 | Eukaryota | Streptophyta | unclassified (derived from Streptophyta) | Celastrales | Celastraceae | *Euonymus* |
| sp342 | Eukaryota | Annelida | Polychaeta | Eunicida | Euphrosinidae | *Euphrosine* |
| sp616 | Eukaryota | Chytridiomycota | Chytridiomycetes | Spizellomycetales | Olpidiaceae | *Olpidium* |
| sp618 | Eukaryota | Chlorophyta | Trebouxiophyceae | Chlorellales | Oocystaceae | *Oocystis* |
| sp637 | Eukaryota | Streptophyta | unclassified (derived from Streptophyta) | Asterales | Asteraceae | *Parthenium* |
| sp649 | Eukaryota | Streptophyta | unclassified (derived from Streptophyta) | Geraniales | Geraniaceae | *Pelargonium* |
| sp651 | Eukaryota | Streptophyta | Jungermanniopsida | Pelliales | Pelliaceae | *Pellia* |
| sp673 | Eukaryota | unclassified (derived from Eukaryota) | unclassified (derived from Eukaryota) | Physariida | unclassified (derived from Physariida) | *Physarum* |
| sp675 | Eukaryota | Chlorophyta | Trebouxiophyceae | unclassified (derived from Trebouxiophyceae) | unclassified (derived from Trebouxiophyceae) | *Picochlorum* |
| sp723 | Eukaryota | Chlorophyta | Prasinophyceae | Pyramimonadales | unclassified (derived from Pyramimonadales) | *Pterosperma* |

| | | | | | | |
|---|---|---|---|---|---|---|
| sp774 | Eukaryota | Streptophyta | unclassified (derived from Streptophyta) | Asterales | Goodeniaceae | *Scaevola* |
| sp775 | Eukaryota | Streptophyta | Jungermanniopsida | Jungermanniales | Scapaniaceae | *Scapania* |
| sp779 | Eukaryota | Streptophyta | Jungermanniopsida | Jungermanniales | Schistochilaceae | *Schistochila* |
| sp781 | Eukaryota | Streptophyta | Coniferopsida | Coniferales | Sciadopityaceae | *Sciadopitys* |
| sp834 | Eukaryota | Streptophyta | Jungermanniopsida | Pallaviciniales | Pallaviciniaceae | *Symphyogyna* |
| sp843 | Eukaryota | Arthropoda | Insecta | Diptera | Tipulidae | *Tanyptera* |
| sp881 | Eukaryota | unclassified (derived from Eukaryota) | Florideophyceae | Nemaliales | Liagoraceae | *Titanophycus* |
| sp885 | Eukaryota | Chlorophyta | Trebouxiophyceae | Microthamniales | unclassified (derived from Microthamniales) | *Trebouxia* |
| sp892 | Eukaryota | Streptophyta | Liliopsida | Poales | Poaceae | *Trisetum* |
| sp899 | Eukaryota | Phaeophyceae | unclassified (derived from Phaeophyceae) | Laminariales | Alariaceae | *Undaria* |

## (50 to 0 cm core depth): temperate subsaline lake

| ID | Domain | Phylum | Class | Order | Family | Genus |
|---|---|---|---|---|---|---|
| sp156 | Archaea | Euryarchaeota | unclassified (derived from Euryarchaeota) | unclassified (derived from Euryarchaeota) | unclassified (derived from Euryarchaeota) | *Candidatus Micrarchaeum* |
| sp395 | Archaea | Euryarchaeota | Halobacteria | Halobacteriales | Halobacteriaceae | *Haloarcula* |
| sp437 | Archaea | Crenarchaeota | Thermoprotei | Desulfurococcales | Desulfurococcaceae | *Ignicoccus* |
| sp252 | Bacteria | Actinobacteria | Actinobacteria (class) | Actinomycetales | Microbacteriaceae | *Curtobacterium* |
| sp253 | Bacteria | Proteobacteria | Betaproteobacteria | Burkholderiales | Comamonadaceae | *Curvibacter* |
| sp294 | Bacteria | Firmicutes | Negativicutes | Selenomonadales | Veillonellaceae | *Dialister* |
| sp318 | Bacteria | Elusimicrobia | Elusimicrobia (class) | Elusimicrobiales | Elusimicrobiaceae | *Elusimicrobium* |
| sp389 | Bacteria | Firmicutes | Bacilli | Lactobacillales | Carnobacteriaceae | *Granulicatella* |
| sp416 | Bacteria | Chloroflexi | Chloroflexi (class) | Herpetosiphonales | Herpetosiphonaceae | *Herpetosiphon* |
| sp444 | Bacteria | Proteobacteria | Betaproteobacteria | Burkholderiales | Oxalobacteraceae | *Janthinobacterium* |
| sp483 | Bacteria | Firmicutes | Bacilli | Lactobacillales | Leuconostocaceae | *Leuconostoc* |
| sp489 | Bacteria | Proteobacteria | Alphaproteobacteria | Rhodobacterales | Rhodobacteraceae | *Loktanella* |
| sp512 | Bacteria | Firmicutes | Bacilli | Lactobacillales | Enterococcaceae | *Melissococcus* |
| sp569 | Bacteria | Tenericutes | Mollicutes | Mycoplasmatales | Mycoplasmataceae | *Mycoplasma* |
| sp614 | Bacteria | Firmicutes | Bacilli | Lactobacillales | Leuconostocaceae | *Oenococcus* |
| sp615 | Bacteria | Proteobacteria | Alphaproteobacteria | Rhizobiales | Bradyrhizobiaceae | *Oligotropha* |
| sp632 | Bacteria | Bacteroidetes | Bacteroidia | Bacteroidales | Porphyromonadaceae | *Parabacteroides* |
| sp648 | Bacteria | Proteobacteria | Alphaproteobacteria | Rhodobacterales | Rhodobacteraceae | *Pelagibaca* |

| | | | | | | |
|---|---|---|---|---|---|---|
| sp658 | Bacteria | Firmicutes | Clostridia | Clostridiales | Clostridiales Family XI. Incertae Sedis | *Peptoniphilus* |
| sp662 | Bacteria | Thermotogae | Thermotogae (class) | Thermotogales | Thermotogaceae | *Petrotoga* |
| sp664 | Bacteria | Proteobacteria | Alphaproteobacteria | Rhodobacterales | Rhodobacteraceae | *Phaeobacter* |
| sp713 | Bacteria | Proteobacteria | Gammaproteobacteria | Alteromonadales | Pseudoalteromonadaceae | *Pseudoalteromonas* |
| sp735 | Bacteria | Actinobacteria | Actinobacteria (class) | Actinomycetales | Micrococcaceae | *Renibacterium* |
| sp749 | Bacteria | Bacteroidetes | Flavobacteria | Flavobacteriales | Flavobacteriaceae | *Riemerella* |
| sp761 | Bacteria | Proteobacteria | Gammaproteobacteria | Aeromonadales | Succinivibrionaceae | *Ruminobacter* |
| sp765 | Bacteria | Actinobacteria | Actinobacteria (class) | Actinomycetales | Pseudonocardiaceae | *Saccharomonospora* |
| sp772 | Bacteria | Actinobacteria | Actinobacteria (class) | Actinomycetales | Sanguibacteraceae | *Sanguibacter* |
| sp791 | Bacteria | Proteobacteria | Betaproteobacteria | Neisseriales | Neisseriaceae | *Simonsiella* |
| sp813 | Bacteria | Actinobacteria | Actinobacteria (class) | Actinomycetales | Glycomycetaceae | *Stackebrandtia* |
| sp838 | Bacteria | Proteobacteria | Deltaproteobacteria | Syntrophobacterales | Syntrophobacteraceae | *Syntrophobacter* |
| sp845 | Bacteria | Proteobacteria | Gammaproteobacteria | Alteromonadales | unclassified (derived from Alteromonadales) | *Teredinibacter* |
| sp847 | Bacteria | Firmicutes | Bacilli | Lactobacillales | Enterococcaceae | *Tetragenococcus* |
| sp871 | Bacteria | Cyanobacteria | unclassified (derived from Cyanobacteria) | Chroococcales | unclassified (derived from Chroococcales) | *Thermosynechococcus* |
| sp888 | Bacteria | Cyanobacteria | unclassified (derived from Cyanobacteria) | Oscillatoriales | unclassified (derived from Oscillatoriales) | *Trichodesmium* |
| sp893 | Bacteria | Actinobacteria | Actinobacteria (class) | Actinomycetales | unclassified (derived from Actinomycetales) | *Tropheryma* |
| sp932 | Bacteria | Actinobacteria | Actinobacteria (class) | unclassified (derived from Actinobacteria (class)) | unclassified (derived from Actinobacteria (class)) | unclassified (derived from Actinobacteria (class)) |
| sp947 | Bacteria | Bacteroidetes | Flavobacteria | Flavobacteriales | Flavobacteriaceae | unclassified (derived from Flavobacteriaceae) |
| sp113 | Eukaryota | Streptophyta | Liliopsida | Poales | Poaceae | *Brachypodium* |
| sp122 | Eukaryota | Chlorophyta | Ulvophyceae | Bryopsidales | Bryopsidaceae | *Bryopsis* |
| sp123 | Eukaryota | Streptophyta | Bryopsida | Bryoxiphiales | Bryoxiphiaceae | *Bryoxiphium* |
| sp134 | Eukaryota | Basidiomycota | Dacrymycetes | Dacrymycetales | Dacrymycetaceae | *Calocera* |
| sp135 | Eukaryota | Ascomycota | Sordariomycetes | Hypocreales | Nectriaceae | *Calonectria* |
| sp183 | Eukaryota | Streptophyta | Liliopsida | Poales | Poaceae | *Centropodia* |
| sp185 | Eukaryota | Ascomycota | Sordariomycetes | Sordariales | Chaetomiaceae | *Chaetomium* |
| sp211 | Eukaryota | Streptophyta | unclassified (derived from Streptophyta) | Canellales | Canellaceae | *Cinnamodendron* |
| sp216 | Eukaryota | Chytridiomycota | Chytridiomycetes | Cladochytriales | Cladochytriaceae | *Cladochytrium* |
| sp222 | Eukaryota | Streptophyta | unclassified (derived from Streptophyta) | Gentianales | Rubiaceae | *Coffea* |

| | | | | | | |
|---|---|---|---|---|---|---|
| sp237 | Eukaryota | Streptophyta | Liliopsida | Poales | Poaceae | *Cortaderia* |
| sp265 | Eukaryota | Streptophyta | Liliopsida | Poales | Poaceae | *Cynodon* |
| sp269 | Eukaryota | Basidiomycota | Dacrymycetes | Dacrymycetales | Dacrymycetaceae | *Dacrymyces* |
| sp271 | Eukaryota | Arthropoda | Branchiopoda | Diplostraca | Daphniidae | *Daphnia* |
| sp343 | Eukaryota | unclassified (derived from Eukaryota) | Spirotrichea | Euplotida | Euplotidae | *Euplotes* |
| sp363 | Eukaryota | Phaeophyceae | unclassified (derived from Phaeophyceae) | Fucales | Fucaceae | *Fucus* |
| sp376 | Eukaryota | unclassified (derived from Eukaryota) | unclassified (derived from Eukaryota) | unclassified (derived from Eukaryota) | Hexamitidae | *Giardia* |
| sp377 | Eukaryota | Ascomycota | Sordariomycetes | Hypocreales | Nectriaceae | *Gibberella* |
| sp488 | Eukaryota | Chlorophyta | Chlorophyceae | Chlamydomonadales | Chlamydomonadaceae | *Lobochlamys* |
| sp566 | Eukaryota | Streptophyta | Zygnemophyceae | Zygnematales | Zygnemataceae | *Mougeotia* |
| sp678 | Eukaryota | Streptophyta | Coniferopsida | Coniferales | Pinaceae | *Pinus* |
| sp722 | Eukaryota | Streptophyta | Polypodiopsida | Polypodiales | Dennstaedtiaceae | *Pteridium* |
| sp731 | Eukaryota | Streptophyta | Jungermanniopsida | Porellales | Radulaceae | *Radula* |
| sp777 | Eukaryota | Chlorophyta | Chlorophyceae | Sphaeropleales | Scenedesmaceae | *Scenedesmus* |
| sp778 | Eukaryota | Chlorophyta | Prasinophyceae | Chlorodendrales | Chlorodendraceae | *Scherffelia* |
| sp794 | Eukaryota | Bacillariophyta | Coscinodiscophyceae | unclassified (derived from Coscinodiscophyceae) | Skeletonemataceae | *Skeletonema* |
| sp889 | Eukaryota | Basidiomycota | Agaricomycetes | Agaricales | Tricholomataceae | *Tricholoma* |
| sp895 | Eukaryota | Basidiomycota | Agaricomycetes | Agaricales | Strophariaceae | *Tubaria* |
| sp925 | Eukaryota | Streptophyta | Liliopsida | Poales | Poaceae | *Zea* |
| sp927 | Eukaryota | Streptophyta | Zygnemophyceae | Zygnematales | Zygnemataceae | *Zygnema* |
